# Long-term push–pull cropping system shifts soil and maize-root microbiome diversity paving way to resilient farming system

**DOI:** 10.1186/s12866-024-03238-z

**Published:** 2024-03-18

**Authors:** Abdul A. Jalloh, Fathiya Mbarak Khamis, Abdullahi Ahmed Yusuf, Sevgan Subramanian, Daniel Munyao Mutyambai

**Affiliations:** 1 International Centre of Insect Physiology and Ecology, P.O. Box 30772-00100, Nairobi, Kenya; 2 Department of Zoology and Entomology, University of Pretoria, Private Bag x20 Hatfield, Pretoria, South Africa; 3 Forestry and Agricultural Biotechnology Institute, University of Pretoria, Private Bag x20 Hatfield, Pretoria, South Africa; 4 Department of Life Sciences, South Eastern Kenya University, P.O. Box 170-90200, Kitui, Kenya

**Keywords:** Agroecosystem sustainability, Amplicon sequencing, Cropping system, Ecosystem services, Soil and maize-root microbiomes, Soil health

## Abstract

**Background:**

The soil biota consists of a complex assembly of microbial communities and other organisms that vary significantly across farming systems, impacting soil health and plant productivity. Despite its importance, there has been limited exploration of how different cropping systems influence soil and plant root microbiomes. In this study, we investigated soil physicochemical properties, along with soil and maize-root microbiomes, in an agroecological cereal-legume companion cropping system known as push–pull technology (PPT). This system has been used in agriculture for over two decades for insect-pest management, soil health improvement, and weed control in sub-Saharan Africa. We compared the results with those obtained from maize-monoculture (Mono) cropping system.

**Results:**

The PPT cropping system changed the composition and diversity of soil and maize-root microbial communities, and led to notable improvements in soil physicochemical characteristics compared to that of the Mono cropping system. Distinct bacterial and fungal genera played a crucial role in influencing the variation in microbial diversity within these cropping systems. The relative abundance of fungal genera *Trichoderma*, *Mortierella*, and *Bionectria* and bacterial genera *Streptomyces*, *RB41*, and *Nitrospira* were more enriched in PPT. These microbial communities are associated with essential ecosystem services such as plant protection, decomposition, carbon utilization, bioinsecticides production, nitrogen fixation, nematode suppression, phytohormone production, and bioremediation. Conversely, pathogenic associated bacterial genus including *Bryobacter* were more enriched in Mono-root. Additionally, the Mono system exhibited a high relative abundance of fungal genera such as *Gibberella*, *Neocosmospora*, and *Aspergillus*, which are linked to plant diseases and food contamination. Significant differences were observed in the relative abundance of the inferred metabiome functional protein pathways including syringate degradation, L-methionine biosynthesis I, and inosine 5'-phosphate degradation.

**Conclusion:**

Push–pull cropping system positively influences soil and maize-root microbiomes and enhances soil physicochemical properties. This highlights its potential for agricultural and environmental sustainability. These findings contribute to our understanding of the diverse ecosystem services offered by this cropping system where it is practiced regarding the system's resilience and functional redundancy. Future research should focus on whether PPT affects the soil and maize-root microbial communities through the release of plant metabolites from the intercrop root exudates or through the alteration of the soil's nutritional status, which affects microbial enzymatic activities.

**Supplementary Information:**

The online version contains supplementary material available at 10.1186/s12866-024-03238-z.

## Introduction

To feed the growing world population, agricultural intensification in staple food crop production such as maize, wheat, and rice has been increasing, leading to increased food security [1, 2]. However, this intensification has also had negative environmental consequences, including increased greenhouse gas emissions, nutrient leaching, soil erosion, and a decline in biodiversity [3, 4]. Ecological diversification that prioritizes environmental quality and preserves beneficial organisms is needed to mitigate these impacts [5–8]. Diversification involves agronomic practices that improve productivity while maintaining long-term stability and resilience and supporting ecosystem services [9]. Intercropping, where farmers grow two or more crops together in an agricultural field, is one such diversification strategy that has been shown to restore ecosystem services and revitalize soil and its associated biodiversity while improving crop yields [2, 10–13].

One such intercropping system that has gained traction in sub-Saharan Africa (SSA) is the push–pull technology (PPT), that has been adopted by thousands of smallholder farmers in East and Southern Africa [14, 15]. Push–pull is an agroecological companion cropping system where the main crop (maize or sorghum) is intercropped with a leguminous plant (*Desmodium* spp.) which serves as an insect-repellent (push), while a grass (Napier or *Briachiaria*) is planted as a border crop to attract stemborers and other herbivores away from the main crop (pull) [16–18]. The push–pull cropping system being a perennial legume-maize intercrop is likely to impact the soil and maize-root microbial communities strongly. The PPT cropping system utilizes volatile chemical mediated tri-trophic interactions where volatile signals emitted by the leguminous plant create an unfavorable environment for oviposition by insect-pests such as *Busseola fusca*, *Chilo partellus*, and more recently *Spodoptera frugiperda* [18–20]. *Desmodium* spp. volatiles are also known to recruit the pests’ natural enemies into the cropping system [14, 20]. The trap crop suppresses the larval development of the insect pest upon hatching from the oviposited eggs [13, 14, 16]. Additional ecological benefits of using PPT include reducing the use of synthetic chemical pesticides and controlling the parasitic weed (*Striga hermonthica*) through the allelopathic effects of the root exudates of the *Desmodium* spp. [19]. Moreover, the leguminous *Desmodium* spp. improves soil health by fixing nitrogen, facilitating carbon sequestration, solubilizing phosphorus, organic matter deposition, and reduction of mycotoxins and other plant pathogens in both soil and maize [3, 21–24]. These changes in soil properties have had positive plant-soil feedback, contributing to increased crop yield on farms practicing the PPT. Thus, this cropping system has been shown to provide diverse ecosystem services, some of which are immediate and well-pronounced, such as insect-pest reduction and crop yield improvement, and long-term effects like the positive plant-soil feedbacks [7, 24].

Soil microbial communities contribute to plant health and yield through root-mediated mechanisms [25]. Plant-associated microbial communities improve productivity and overall plant health by ensuring nutrient availability, stress tolerance, disease resistance, and biodiversity enhancement [25, 26]. The genotype of a plant determines its root-associated microbiota, and the plant, in turn, can shape the belowground microbiome by supporting or suppressing local microbial populations [2, 5]. Plants use root exudates to actively modify soil microbial populations favoring beneficial microbes [6, 27]. Legume-based intercropping can improve soil health and optimize the soil's physicochemical properties by increasing relative microbial abundance and organic matter, controlling soil erosion, and improvement of soil structure [26, 28, 29]. In addition, intercropping with legumes positively affects soil's chemical properties, including soil organic carbon (OC) concentration, nutrient content, and cation exchange capacity (CEC) [2, 22]. Legumes can also change the soil pH, thereby affecting soil microbial activity [26]. Previous studies have shown that cereal-legume intercropping systems result in increased soil microbial biomass and enhanced nutrient availability, particularly nitrogen, phosphorus, and carbon [2, 28]. Wheat (*Triticum aestivum*) or maize (Zea *mays* L.)-faba bean (*Vicia faba*), [30] and durum wheat (*Triticum turgidum durum*)-chickpea (*Cicer arietinum*) or lentil (*Lens culinaris*), [5] maize-peanut (*Arachis hypogaea*), [31] intercropping has been shown to increase overall microbial diversity. The presence of pathogenic microbial communities of genera *Aspergillus*, *Gibberalla*, and *Bryobacter* in higher abundances in monoculture cropping systems, further supports the hypothesis that diversified cropping systems harbor more beneficial microorganisms. This, in turn, enhances soil nutrients, plant development, and disease management [32–34].

In this study, we assessed the impact of the PPT cropping system on the soil and maize-root microbiome and soil physicochemical properties in smallholder farmers’ fields (SHFF) where there are variable climatic and edaphic factors. We hypothesized that the PPT cropping system influences soil physicochemical parameters and shifts soil and maize-root microbiome in favor of ecologically essential groups, as compared to the maize-monoculture (Mono) cropping system. While the components and aboveground multitrophic interactions of the PPT cropping system and the underlying mechanisms, have been studied to a greater extent, the belowground multitrophic interactions, including their impact and interactions with soil and root microbiome, have not yet received similar attention. Therefore, it is crucial to investigate the impact of this functional cropping system on the soil and maize-root microbiome and the subsequent cascading effects on the aboveground tri-trophic interactions.

## Materials and methods

### Description of sampling site

Soil and maize-root samples were collected from three Counties in western Kenya– Vihiga (S 0° 1′ 53.06'' E 34° 34′ 0.05''; N 0° 0′ 42.85'' E 34° 35′ 29.6''), Siaya (N 0° 2′ 26.8'' E 34° 18′ 19.5''; S 0° 0′ 0.23'' E 34° 16′ 13.23''), and Bungoma (N 0° 37′ 12.8'' E 34° 33′ 55.2''; N 0° 35′ 01.3'' E 34° 36′ 14.4'') (Additional file 1: Fig. S1). Different maize cropping systems, including push–pull and Mono farm fields, are already established in these areas for over 6 to 20 years [14, 24]. The samples were collected from 18 SHFF (9 each with PPT and Mono cropping systems) which shared comparable agronomic management practices without the use of pesticides or synthetic chemical fertilizers and had minimal cultivation for weed management. The Mono and PPT farms had a similar ground cover, except that *Desmodium* spp. dominated the PPT farms. The three counties in the study area experience bimodal rainfall, with a long rainy season from March to August and a short rainy season from October to December. The western Kenya region has a hot and humid climate, abundant sunshine throughout the year, and an average daily temperature of around 25 ± 2 °C. There were altitudinal and rainfall variations among the selected counties: Vihiga at 1594.28 m above sea level (masl), with a rainfall range of 1800–2000 mm per annum (p.a); Siaya at 1140 masl, with a rainfall range of 1200–1800 mm p.a; and Bungoma at 1385 masl, with a rainfall range of 1102–1800 mm p.a. [13, 17].

### Sample collection

Soil and maize-root samples were collected from 9 farms each of PPT and Mono cropping systems in different SHFF when the maize plants were in the late vegetative development stage (V5-6), about five to six weeks old. Soil sampling was done randomly (10 sampling points) between rows and pooled. Ten samples were collected per SHFF (about 5–20 cm depth) using a soil auger after cleaning the surface organic matter around the rows of the plants. The collection was halfway between the *Desmodium* spp. and maize rows on PPT farms and the same distance between maize rows in Mono farms. The pooled soil samples were placed in a 20 mL centrifuge tube (Thermo Fisher Scientific Inc., California, USA), put in a cool box with ice packs, and immediately transported to the laboratory at the International Centre of Insect Physiology and Ecology (*icipe*), Nairobi, Kenya where they were stored at -80 °C until needed for DNA extraction. A subset of soil samples was kept in brown Khaki paper bags (Paper Bags Ltd., Nairobi, Kenya) for 48 h under room temperature, and used later for soil physicochemical parameters analysis. Maize-root samples from ten plants located next to the soil cores were collected from the root systems to a depth of approximately 10 cm in each sampled plot, and then pooled. The maize-roots were rinsed to remove soil debris, surface sterilized by immersing them in 70% ethanol and 1% sodium hypochlorite for 1 min each, followed by washing six times in sterilized distilled water. To confirm sterility, 0.1 mL of the final rinsing water was spread plate on yeast extract mannitol agar (YEMA) with Congo red (0.025% w/v) and lysogeny broth (LB) and incubated at 33 ± 2 ºC for 48 h for bacteria growth. Similarly, for fungi, 0.1 mL of the final rinsing water was spread plate on potato dextrose agar (PDA) and sabouraud dextrose agar (SDA), and incubated at 25 ± 2 ºC for 72 h. After sterilization, the maize-root samples were air-dried on sterile blotting paper in a sterilized laminar flow cabinet (Class II Type A/B3, Biological Safety Cabinet, The Bakey Company, Sanford, Maine, USA), and then stored at -80 °C until DNA extraction commenced. Soil physicochemical parameters were determined following standard protocols detailed by Chen et al. [35] and Okalebo et al. [36].

### Genomic DNA extraction

Based on a method used by Howard et al. [37], high-throughput amplicon sequencing of the V1-V4 region of the 16S rDNA gene regions and internal transcribed spacer (ITS; ITS1-ITS2) gene regions were used for bacterial and fungal metagenomics sequencing. PureLink™ Microbiome DNA purification kit (Thermo Fisher Scientific Inc., California, USA) was utilized for the extraction of soil total genomic deoxyribonucleic acid (gDNA), following the manufacturer’s instructions. Each sample was extracted twice, and the supernatants were mixed to obtain more DNA. Power Bead Tubes were filled with 0.25 g of soil and vortexed for 10 s. The lysis buffer was mixed using a vortex mixer, and the bead tube was then heated for 10 min at 65 °C, followed by homogenization through vortexing at 14,000 rpm for 5 min. The supernatant was processed using spin columns, and the DNA was extracted using Tris–HCL. The quality of the extracted DNA was analyzed using a Thermo Scientific Nanodrop 2000 (UV–Vis spectrophotometer) and gel electrophoresis. The DNA samples were stored at -80 °C until further processing [7].

For maize-root samples, lyophilization was carried out for 48 h, and the maize-root were ball-milled into a fine powder for gDNA extraction. After that, a plant DNA extraction kit ((Isolate II) Thermo Fisher Scientific Inc., California, USA) was used to extract DNA from 0.25 g of maize-root (dry weight). Briefly, 0.25 g of powdered maize-root sample was put into lysis buffer-filled bead tubes and vortexed for 10 s. The mixture was centrifuged for two minutes at 14,000 rpm. After discarding the pellet, the supernatant was centrifuged on a spin column to extract DNA following the manufacturer’s instructions. A Thermo Scientific NanoDrop™ 2000 (UV–Vis spectrophotometer) and gel electrophoresis were used to assess the quality and size of the extracted DNA. The DNA samples were stored at -80 °C until further processing [38].

### PCR, library preparation, and sequencing

Amplicon sequencing targeting V1-V4 regions of 16S rDNA gene and ITS1-ITS2 regions of ITS gene was carried out at Macrogen Europe in the Netherlands using the MiSeq (Illumina) instrument, following the manufacturers instructions. For the 16S rRNA (bacterial) gene amplicon library, PCR primers were used to target both the V1-V2 regions (27F` GAGTTTGATCMTGGCTCAG, 338R` GCTGCCTCCCGTAGGAGT) and the V3-V4 regions (341F` CCTACGGGNGGWGCAG and 805R` -GACTACHVGGGTATCTAATCC). For the ITS gene (fungal), PCR primer pairs targeted the ITS1 and ITS2 regions (ITS1F` CTTGGTCATTTAGAGGAAGTAA, ITS2R` -GCTGCGTTCTTCATCGATGC) and the ITS3-ITS4 regions (ITS3F` GCATCGATGAAGAACGCAGC ITS4R` -TCCTCCGCTTATTGATAGC) [6, 8, 12]. The libraries were sequenced using a sequencing mode which was set to paired-end two 300-cycle sequencings, and a 600-cycle v3 sequencing kit was used.

### Statistical analyses

The data for soil physicochemical characteristics were tested for normality using the Shapiro–Wilk test. Because the data was normally distributed, parametric statistical tests were used. One-way analysis of variance (ANOVA) was used to examine the soil characteristics based on cropping systems. Where differences existed, a Tukey HSD test was used to analyze the differences in soil characteristics [35]. A Pearson correlation coefficient analysis was used to detect the relationships between soil conditions and different cropping systems. The profile of soil physicochemical properties was visualized using non-metric multidimensional scaling (NMDS) for both cropping systems, PPT and Mono. To compare differences between these cropping systems, a one-way ANOSIM test was conducted using the Bray–Curtis dissimilarity matrix. All statistical analyses were carried out using R software (v4.1.2) [39].

### Bioinformatics

Raw reads were processed using nf-core/ampliseq (v2.4.0) (https://github.com/nf-core/ampliseq) metagenomic amplicon pipeline deployed using nextflow (v21.10.3) and singularity (v3.6.3) [40]. Sequence quality control was assessed using FastQC (v0.11.6). Poor reads and primers were trimmed using Cutadapt (v.4.1) [6]. The Divisive Amplicon Denoising Algorithm 2 (DADA2) analysis workflow option of the nf-core/ampliseq pipeline was used to assess soil and maize-root prokaryotic and mycobiome communities. The DADA2 (v1.26.0) analysis workflow was used to denoise, preprocess, infer amplicon sequence variants (ASVs), and assign taxonomy of the clean 16S rRNA and ITS sequence reads [41]. The DADA2 functions parameters used to perform further trimming included truncLen = 180, trunc_qmin = 25, trunc_rmin = 0.75, max_ee = 2, min_len = 50, and maxN = 0. After preprocessing, the reads were dereplicated using the “derepFastq” and “dada” functions, inferring amplicon sequence variants (ASVs) and their counts. The “concatenate_reads,” “sample_inference,” and “removeChimeraDenovo” functions were applied to remove spurious and chimeric ASVs. The “dada_ref_taxonomy” function utilized the “silva = 138” database to assign taxonomy to 16S bacterial ASV [42] and “unite-fungi = 8.3” to assign taxonomy to ITS fungal ASVs [43]. The ASV relative abundance tables and ASV taxonomic classification output files obtained from the DADA2 pipeline were used for downstream data exploration, statistical analysis, and visualization in R (v4.2.1). The Basic Rapid Ribosomal RNA Predictor (Barrnap) (v0.9) [44] utilized the ASV nucleotide base sequences to classify them into various categories, which include eukaryotes, archaea, bacteria, chloroplast, and mitochondria [45]. The ASV filtering for bacteria was done to exclude all archaea, eukaryotic, chloroplast and mitochondria, while for fungi, we excluded only archaea, chloroplast, and mitochondria. The soil and maize-root microbial communities were determined by merging the ASV relative abundance matrix and taxonomy table generated by the DADA2 analysis workflow with the metadata file containing descriptions, treatments, and conditions for each sample collected, forming a phyloseq object. The phyloseq (v1.41) package was used to determine the differential abundance of microbes under different conditions of alpha and beta diversity [40]. The metagMisc (v0.0.4) [46] package was utilized to manipulate the phyloseq object for visualization of the microbiome relative abundances and percentages in the sample types (soil and maize-root), cropping systems (PPT and Mono), and sampling locations (Bungoma, Siaya and Vihiga).

### Alpha and beta diversity

The Microbiota Process (v1.9.3) function was used to analyze the species richness and diversity of soil and maize-root prokaryotic and mycobiome communities by performing rarefaction analysis on the ASV read counts [47]. The analysis employed alpha diversity metrics, including Chao*1*, *Evenness*, and *Shannon* indices. A Principal Coordinate Analysis (PCoA) was used to identify the microbial contributors and their variation in beta diversity [48]. Venn diagrams were generated using Venn Counts function from the LIMMA (linear models for microarray data) package to display the shared microbial communities among the sample types, cropping systems, and sampling locations [49]. Before conducting Permutational Multivariate Analysis of Variance (PERMANOVA), we utilized betadisper (using Vegan package) with each farm treated as stratum to compare microbial populations across the different sample types, cropping systems, and locations [48, 50]. The diff_analysis and ggdiffclade functions of the Microbiota Process utilized the phyloseq object to determine biomarkers and visualize the microbes (fungal and bacterial) at the genus level.

#### Differential expression of soil and maize-root bacterial protein functions in push–pull and maize-monoculture cropping systems

The BIOM (biological observation matrix) file was utilized for the phylogenetic investigation of communities by reconstructing unobserved states (PICRUSt2) (v2.5.0) tool to predict microbial functions in the soil and maize-root. The PICRUSt2 pipeline (v2.4.1) was utilized to predict functional abundances based on ASV marker gene sequences. The resulting bacterial abundances were classified using the enzyme commission classification and statistically compared across cropping systems, sample types, and sampling locations. Phylogenetic heat maps of pathways were visualized using the STAMP software (v2.1.3) for further analysis [51, 52].

## Results

### Soil physicochemical properties from push–pull and maize-monoculture cropping systems

Soil pH, organic carbon, nitrogen, and potassium from PPT soil were significantly different compared to that of Mono cropping system soil (ANOVA; *P* < 0.001; Table 1). However, there was no significant difference between PPT and Mono cropping systems among the other soil parameters. The PPT soil texture ranged from sandy loam to sandy clay loam, with characteristics including sand (21.77–74.19%), silt (10.92–34.38%), and clay (14.89–43.85%). In contrast, soil texture for the Mono cropping system was loamy sand, sandy loam to clay loam, with soil characteristics including sand (36.78–86.53%), silt (8.48–39.23%), and clay (3.99–38.43%).

**Table 1:** Comparison of soil physicochemical properties between push–pull and maize-monoculture cropping systems in smallholder farmer fields

Soil properties	Cropping systems		*F* value	*P* value
	**PPT**	**Mono**	^**(1,18)**^	
**pH** (H_2_O)	5.673 ± 0.071^a^	4.938 ± 0.099^b^	36.580	**< 0.001**
**EC** (mmhos/cm)	0.081 ± 0.016^a^	0.051 ± 0.011^a^	2.329	> 0.144
**P** (mg/kg)	22.999 ± 5.024^a^	16.647 ± 2.233^b^	1.335	**< 0.001**
**K** (mg/kg)	91.981 ± 7.631^a^	83.324 ± 11.124^a^	0.412	> 0.412
**Na** (mg/kg)	9.699 ± 0.187^a^	9.691 ± 0.253546^a^	0.001	> 0.980
**Ca** (mg/kg)	808.180 ± 123.026^a^	802.239 ± 130.529^a^	0.001	> 0.974
**Mg** (mg/kg)	137.299 ± 18.709^a^	136.470 ± 19.357^a^	0.001	> 0.976
**Fe** (mg/kg)	110.892 ± 8.929^a^	104.926 ± 13.713^a^	0.133	> 0.721
**Mn** (mg/kg)	208.095 ± 33.248^a^	180.074 ± 29.832^a^	0.393	> 0.538
**Cu** (mg/kg)	2.552 ± 0.429^a^	1.978 ± 0.253^a^	1.330	> 1.332
**Mo** (mg/kg)	0.020 ± 0.001^a^	0.020 ± 0.001^a^	2.250	> 0.151
**Zn** (mg/kg)	3.585 ± 0.649^a^	3.665 ± 1.121^a^	0.004	> 0.954
**B** (mg/kg)	1.425 ± 0.012^b^	1.458 ± 0.010^a^	4.453	**< 0.010**
**S** (mg/kg)	11.325 ± 1.376^a^	8.810 ± 1.069^a^	2.083	> 0.166
**N** (%)	0.146 ± 0.009^a^	0.068 ± 0.013^b^	23.400	**< 0.001**
**OC** (%)	1.319 ± 0.132^a^	0.693 ± 0.146^b^	10.170	**< 0.001**
**EA** (meq/100 g)	1.180 ± 0.131^a^	1.060 ± 0.109^a^	0.498	> 0.489
**ESP** (%)	0.972 ± 0.166^a^	0.927 ± 0.126^a^	0.046	> 0.832

*PPT* Push–pull technology, *Mono* Maize-monoculture cropping system, *pH* Potential of hydrogen, *EC* Electrical conductivity, *P* Phosphorus, *K* Potassium, *Na* Sodium, *Ca* Calcium, *Mg* Magnesium, *Fe* Iron, *Mn* Manganese, *Cu* Copper, *Zn* Zinc, B Boron, *Mo* Molybdenum, S Sulphur, *N* Nitrogen, *OC* Organic carbon, *EA* Exchangeable acidity, *ESP* Exchangeable sodium percentage, *mmhos/cm* Millimhos per centimeter, *mg/kg* Milligrams per kilogram, *%* Percentage, *meq/100 g* Millequivalents per 100 g of soil

The different letter indicates a significant difference between the two cropping systems (Significant effects at *P* < 0.05 are shown in bold, Tukeys honest significance test (HSD)

**Fig. 1:**
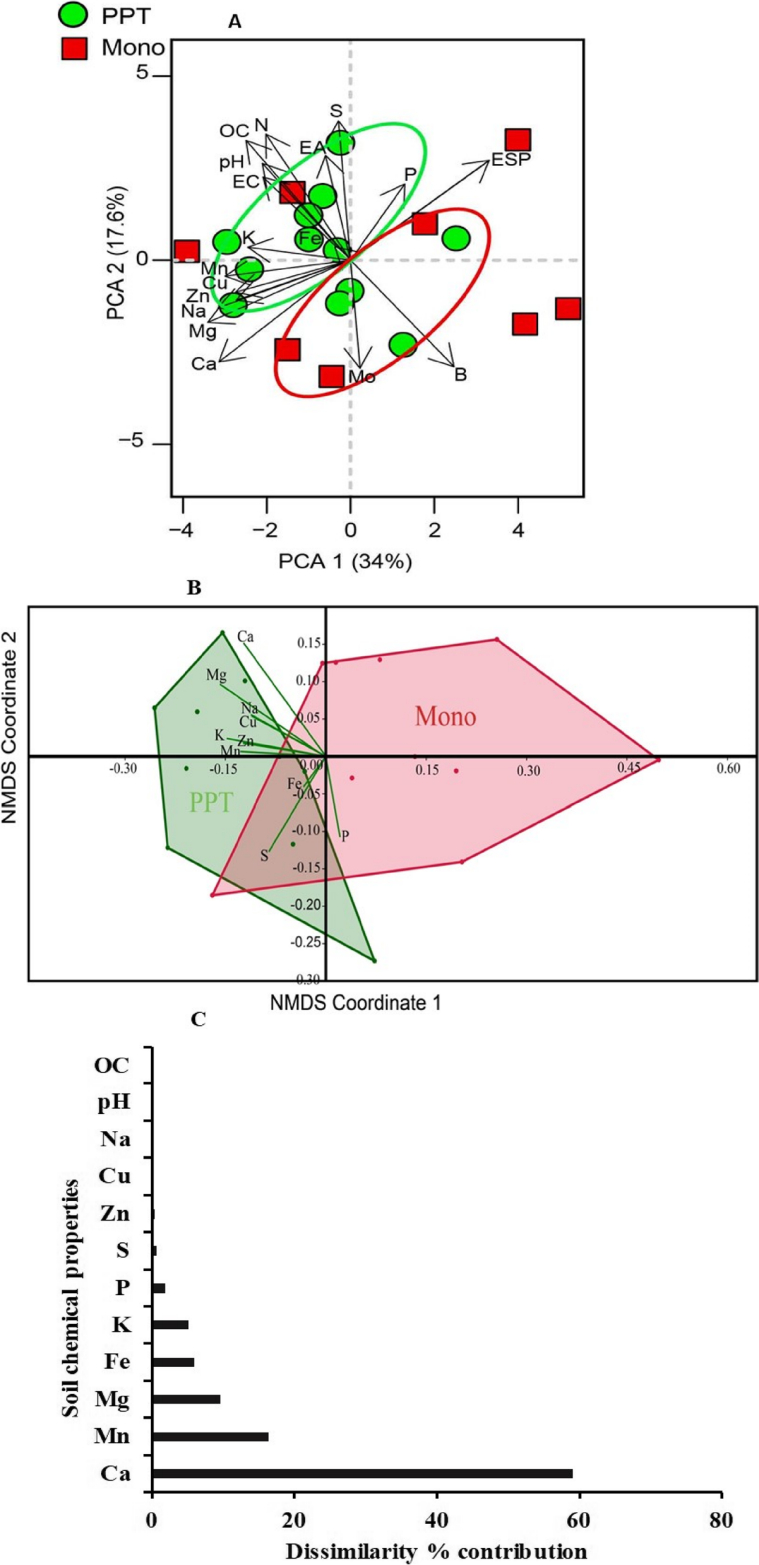
Impact of push–pull cropping system on soil physicochemical properties: **A** Principal component biplot depicting the relationship between soil physicochemical properties and their association with cropping systems. 1 = Dim1 for principal component and 2 = Dim2 for principal component; **B** Non-metric multidimensional scaling (NMDs; distance Bray–Curtis) showing the clustering relationships between the different soil physicochemical properties; **C** Histogram showing the % predominant contribution of the soil chemical properties based on similarities. PPT, push–pull technology; Mono, maize-monoculture cropping system; pH, potential of hydrogen; EC, electrical conductivity; P, phosphorus; K, potassium; Na, sodium; Ca, calcium; Mg, magnesium; Fe, iron; Mn, manganese; Cu, copper; Zn, zinc; B, boron; Mo, molybdenum; S, sulphur; N, nitrogen; OC, organic carbon; EA, exchangeable acidity; ESP, exchangeable sodium percentage

Within the PPT field, several soil properties such as sulphur, nitrogen, organic carbon, exchangeable acidity, pH, electrical conductivity, potassium, and iron revealed positive association, showcasing distinct characteristics within this cropping system. In contrast, the Mono cropping system exhibited no discernible impact on these soil properties. However, soil properties such as boron, molybdenum, calcium, and exchangeable sodium percentage were more abundant in Mono cropping system (Fig. 1A). Notably, PC1 and PC2, which accounted for 34% and 17.6% of the total variance, respectively, played a vital role in elucidating the interactions between the selected soil properties. The Correlograms (Additional file 1: Fig. S2) showed the relationships and correlations between the different soil physicochemical properties in the PPT and Mono cropping systems.

The non-metric multidimensional scaling (NMDS) plot, using the Bray–Curtis similarity index, revealed no significant difference between the soil physicochemical properties tested in PPT and Mono cropping systems (One-way ANOSIM: *P* = 0.9591, *R* = -0.078; *Stress value* = 0.1894) (Fig. 1B). However, the analysis of similarities (ANOSIM) showed that certain soil physicochemical properties contributed most of the differences between the PPT and Mono cropping systems. Interestingly, the properties that contributed positively to the differences in the PPT cropping system compared to Mono are as follows: calcium (59.20%); manganese (16.34%); magnesium (9.59%); iron (6.02%); potassium (5.18%); phosphorus (1.87%); sulphur (0.69%); zinc (0.40%); copper (0.16%); sodium (0.11%); pH, (0.11%); electrical conductivity (0.11%); and organic carbon (0.11%) (Fig. 1C). These properties collectively accounted for the observed differences between the two cropping systems (Fig. 1A-C).

#### Relative abundance of soil and maize-root microbiome in push–pull and maize-monoculture cropping system fields

Profiling the bacterial community yielded 5,714,532 high-quality sequences, averaging 150,382.4 per sample with a range of 237,684 to 80,956. For fungal sequences, a total of 5,466,660 high-quality sequences were obtained, with a mean of 165,656.4 per sample, and each sample generated a read between 233,381 and 123,338. However, after the rarefaction of the sequences (Additional file 1: Fig. S3) and removal of non-fungal and non-bacterial sequences, we found 3,953 fungal and 7,556 bacterial amplicon sequence variants (ASVs) in all soil and maize-root samples.

### Taxonomic profiles of belowground fungal and bacterial communities

**Fig. 2:**
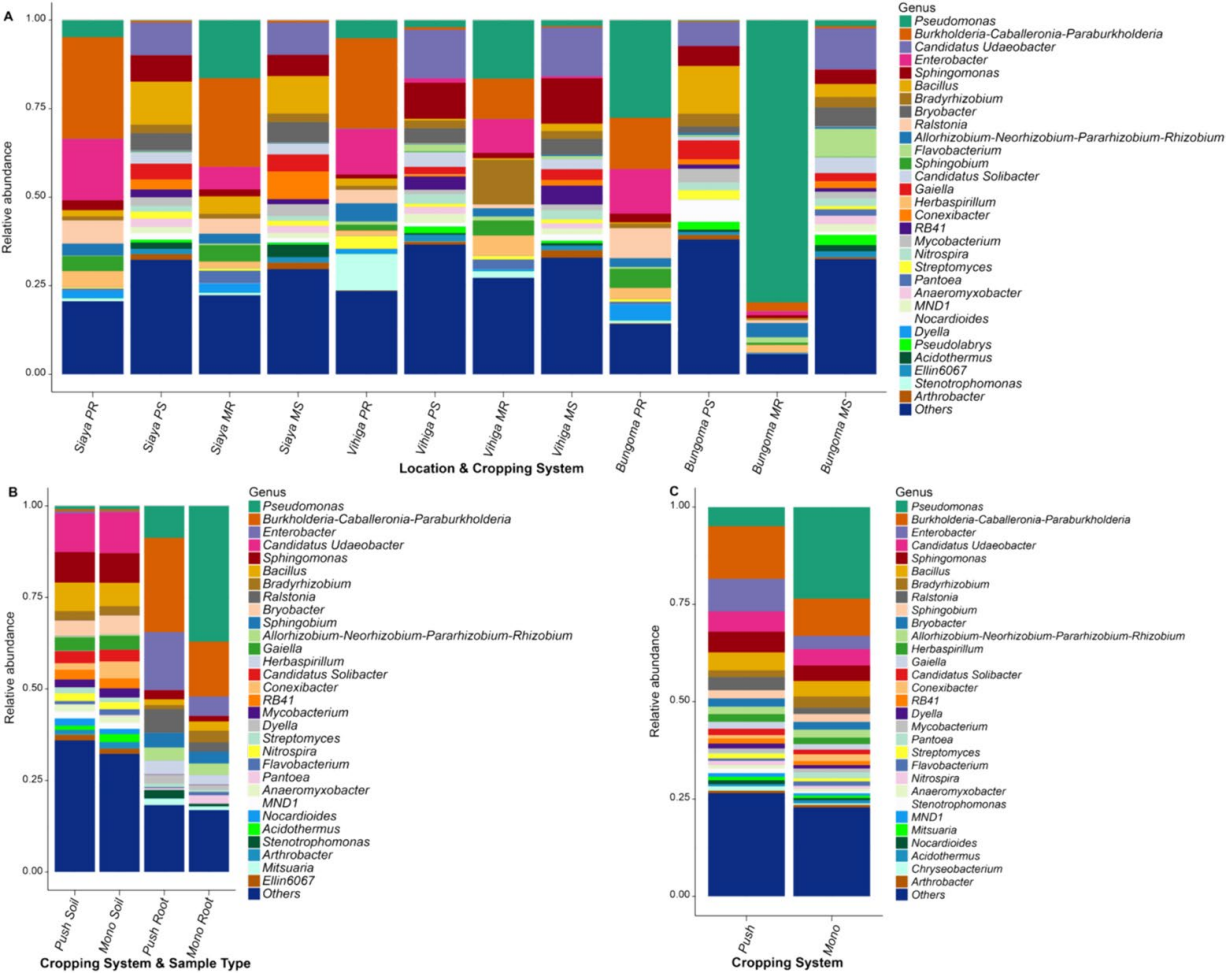
Relative abundance of bacterial genera found in push–pull and maize-monoculture cropping systems by; **A** Location, sample type, and cropping system; PR, push–pull root; PS, push–pull soil; MR, maize-monoculture root; MS, maize-monoculture soil; **B** Cropping system and sample type; Push soil (push–pull soil); Mono soil (maize-monoculture soil); Push root (push–pull root); Mono root (maize-monoculture root); **(C)** Cropping systems; Push (push–pull technology (soil + maize-root)); Mono (maize-monoculture cropping system (soil + maize-root)). Bacterial genera with lower than 1% relative abundances were grouped as 'Others'

The differences between the maize-root and soil bacterial and fungal genera were evident in the ASVs across all samples. We found the 30 most relatively abundant bacterial genera communities in PPT and Mono cropping systems (Additional file 1: Figs. S4 and S5). The order of relative abundance in PPT (soil and maize-root) compared to the Mono (soil and maize-root) cropping system was as follows: *Enterobacter* > , *Sphingomonas* > , *Candidatus Udaeobacter* > , *Sphingobium* > , *RB41* > , *Stenotrophomonas* > , *Streptomyces* > , *Nitrospira* > , and *Mitsuaria* (Fig. 2; Additional file 1: Table S1). *Pseudomonas* > , *Bryobacter* > , *Conexibacter* > , *Acidothermus* > , and *Pantoea* were the most relatively abundant genera in the Mono cropping system compared to that of PPT. However, the interaction between sample type (soil and maize-root), and PPT and Mono cropping system (PPT soil and PPT maize-root, and Mono soil and Mono maize-root) had varying effects on the impact of the bacterial genera communities (Fig. 2; Additional file 1: Table S2). In regard to study sites, the most enriched bacterial genera in Bungoma in the order of relative abundance were *Pseudomonas* > , *Flavobacterium* > , and *Nocardioides* compared to Siaya and Vihiga. *Streptomyces* > , *Bacillus* > , *Sphingobium* > , and *RB41* were the most enriched genera in Siaya compared to the other two counties. *Candidatus Udaeobacter* > , *Bradyrhizobium* > , *Enterobacter* > , *Sphingomonas* > , *Nitrospira* > , and *Stenotrophomonas* were more relatively abundant in Vihiga compared to Bungoma and Siaya counties (Additional file 1: Fig. S6; Table S3). However, PPT positively impacted bacterial genera in different sample types between the cropping system and study locations. *Burkholderia-Caballeronia-Paraburkholderia* were more relatively abundant in Siaya PPT maize-root compared to the other cropping system, sample types, and study locations. *Acidothermus* > *,* and *Pantoea* were more abundant in Siaya Mono maize-root, and *Conexibacter* enriched in Siaya Mono soil (Fig. 2; Additional file 1: Table S4**)**. The relative abundance of *Allorhizobium-Neorhizobium-Pararhizobium-Rhizobium* was higher in Vihiga PPT maize-root compared to the other cropping systems, sample types, and study locations, while *Enterobacter* > , and *Streptomyces* exhibited higher relative abundance in Vihiga PPT soil. *Bradyrhizobium* > , and *Stenotrophomonas* were more relatively abundant in Vihiga Mono maize-root. *Arthrobacter* > , and *Nitrospira* were more enriched in Vihiga Mono soil. *Dyella* > , and *Ralstonia* were more relatively dominant in Bungoma PPT maize-root compared to the other cropping system, sample types, and study locations. *Gaiella* > , *Nocardioides* > , and *Sphingobium* were more relatively abundant in Bungoma PPT soil. *Bryobacter* > , and *Sphingomonas* were more enriched in Bungoma Mono soil. There was an increase of *Pseudomonas* bacteria in Bungoma Mono maize-root compared to the other cropping systems, sample types, and study locations.

The most highly enriched species in PPT (soil and maize-root) compared to that of Mono (soil and maize-root) cropping system in the order of relative abundance were *Rhizobium phaseoli* > , *Bacillus flexus* > , *Bradyrhizobium elkanii* > , *Paraburkholderia vietnamiensis* > , *Dyella marensis* > , *Enterobacter hormaechei* > , *Herbaspirillum seropedicae* > , *Pseudomonas nitroreducens* > , *Ralstonia pickettii* > , *Sphingomonas paucimobilis* > , *Stenotrophomonas maltophilia* > , and *Variovorax paradoxus*.

**Fig. 3:**
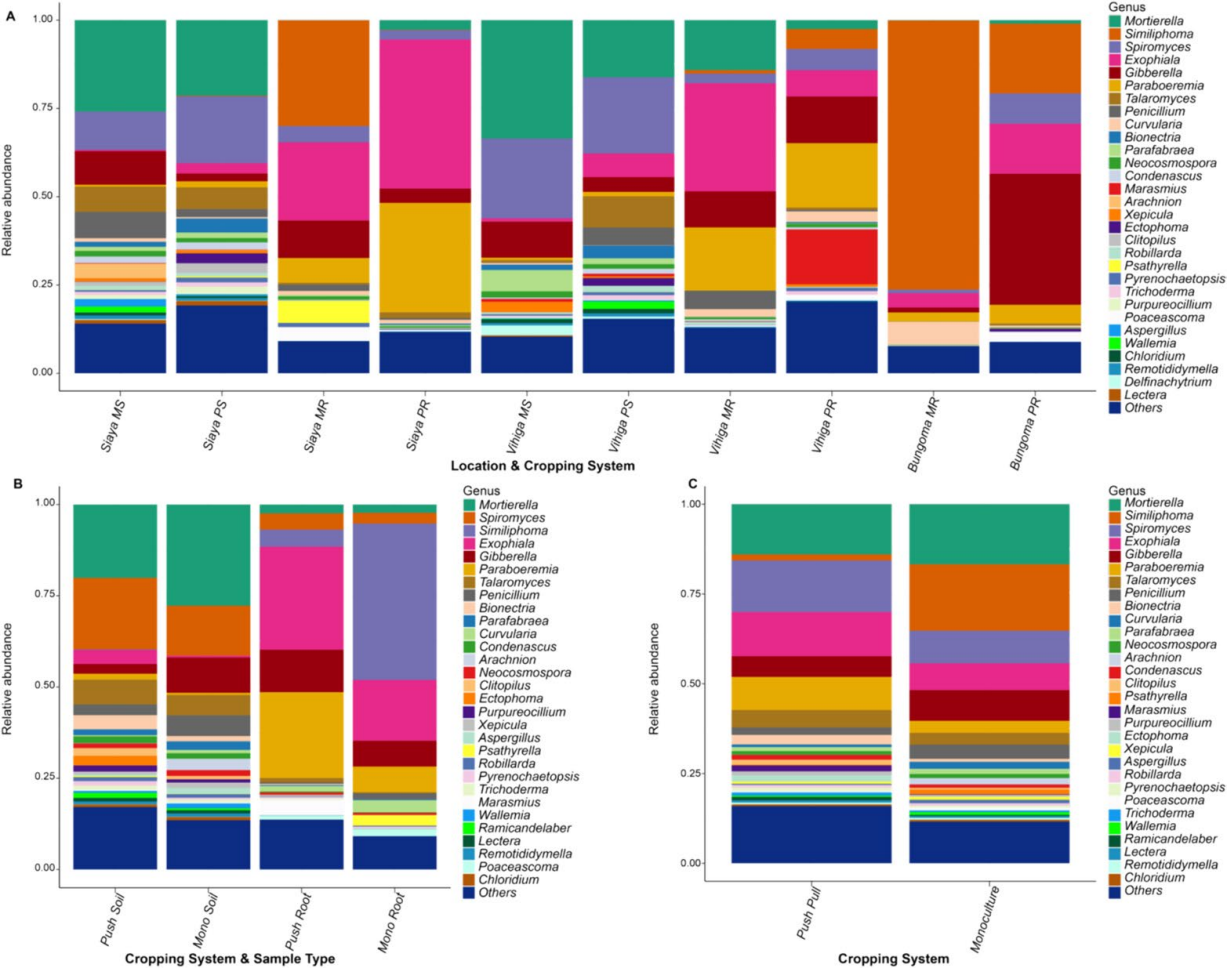
Relative abundance of fungal genera found in push–pull and maize-monoculture cropping systems by; **A** Location, sample type, and cropping systems; PR, push–pull root; PS, push–pull soil; MR, maize-monoculture root; MS, maize-monoculture soil; **B** Cropping & sample type; Push soil, push–pull soil; Mono soil, maize-monoculture soil; Push root, push–pull root; Mono-root, maize-monoculture root; **C** Cropping systems; Push pull (soil + maize-root); monoculture (soil + maize-root). Fungal genera with relative abundances lower than 1% were grouped as 'Others'

In fungal genera communities, the predominant genera in PPT (soil and maize-root) in the order of relative abundance were *Mortierella* > , *Spiromyces* > , *Bionectria* > , *Clitopilus* > , *Marasmius* > , *Trichoderma* > , and *Ramicandelaber* compared to that of Mono (soil and maize-root) cropping system. *Gibberella* > , *Similiphoma* > , *Neocosmospora* > , *Aspergillus* > , and *Psathyrella* were more relatively abundant in the Mono cropping system compared to that of PPT (Fig. 3; Additional file 1: Table S5). *Candenascus*, *Xepicula*, and *Chloridium* had the same relative abundance in PPT and Mono cropping systems. However, the interaction between cropping systems (PPT and Mono) and sample types (PPT soil and PPT maize-root, and Mono soil and Mono maize-root) had varying effects on the impact of the fungal genera communities. *Arachnion* > , *Bionectria* > , *Spiromyces* > , and *Trichoderma* were more enriched in PPT soil, while *Exophiala* > , *Marasmius* > , and *Poaceascoma* were the most relatively abundant in PPT maize-root compared to Mono soil and Mono maize-root (Fig. 3; Additional file 1: Table S6). However, *Aspergillus* > , *Condenasus* > , *Neocosmosphora* > , *Parafabraea* > , and *Xepicula* were more abundant in Mono soil. At the same time, *Curvularia* > , *Psathyrella* > , and *Similiphoma* were more relatively abundant in Mono maize-root compared to PPT soil and PPT maize-root. The three study counties had varying effects on the impact of fungal genera communities (Additional file 1: Fig. S7; Table S7). However, the impact of the fungal genera was felt differently when PPT and Mono cropping systems interacted with the sample types, study locations, and cropping systems (Fig. 3; Additional file 1: Table S8).

**Fig. 4:**
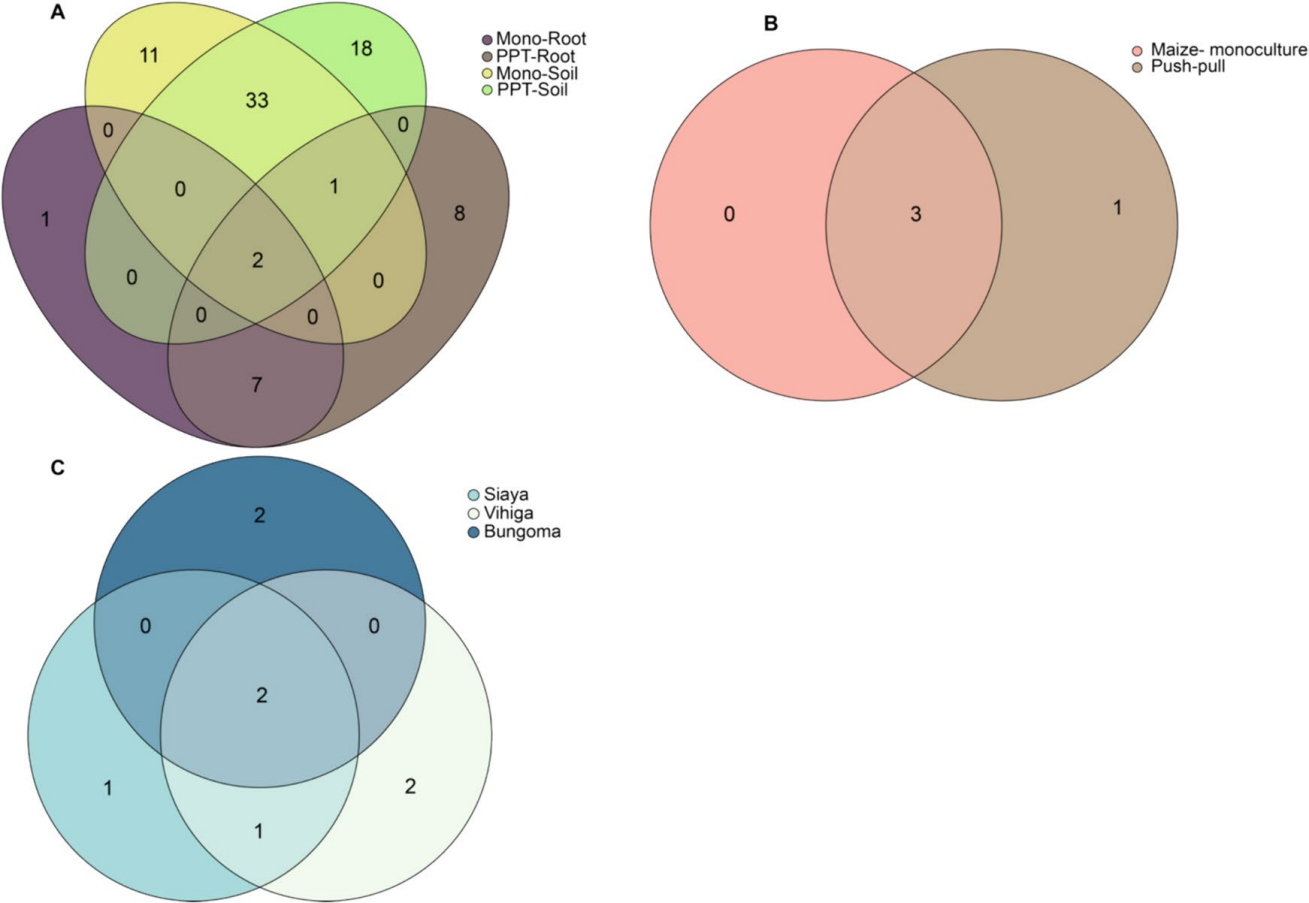
Bacterial genera shared between **A** Cropping systems and sample types; Mono-Root, maize-monoculture root; PPT-root, push–pull maize-root; Mono-soil, maize-monoculture soil; PPT-soil, push–pull soil; **B** Cropping systems; Push–pull, push–pull technology (soil + maize-root); Maize-monoculture (soil + maize-root); **C** Locations

**Fig. 5:**
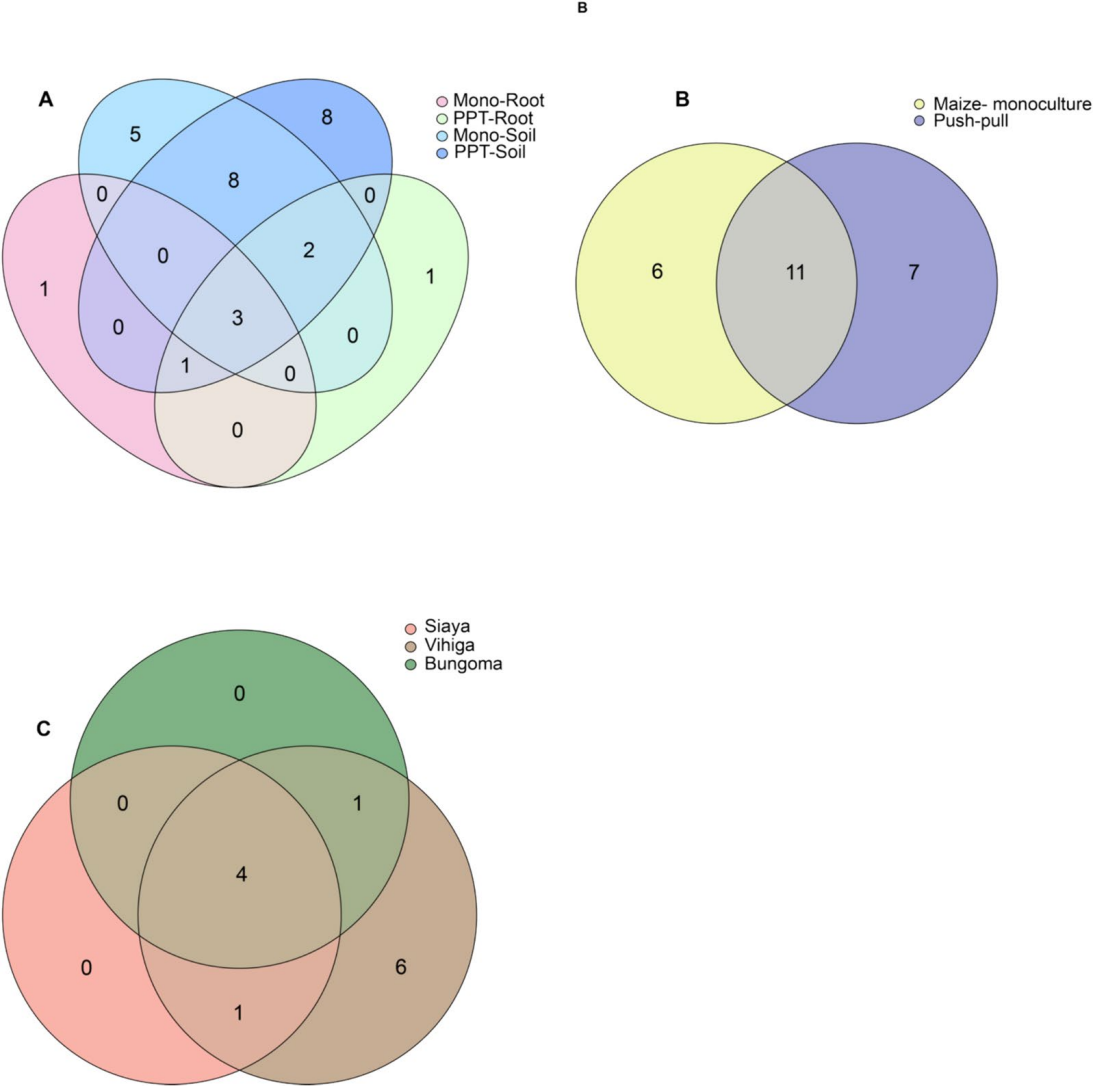
Fungal genera shared between **A** Cropping systems and sample types; Mono-Root, maize-monoculture root; PPT-root, push–pull maize-root; Mono-soil, maize-monoculture soil; PPT-soil, push–pull soil; **B** Cropping systems; Push–pull, push–pull technology (soil + maize-root); Maize-monoculture (soil + maize-root); **C** Locations

In 90% of the sample ASVs, the most prevalent and distinguishing genera were present, each with a 75% prevalence in every ASV. A Venn diagram showed that PPT had one unique bacterial genus compared to the Mono cropping system, with three overlapping bacterial genera shared between the two cropping systems. When we compared cropping systems, and sample types interaction, PPT soil and PPT maize-root, we found 18 and eight unique bacteria, respectively, in comparison to Mono soil and Mono maize-root, which had 11 and one bacterium taxon (Fig. 4). In terms of the studied counties, Bungoma and Vihiga each harbored two distinct bacterial genera. In contrast, Siaya had only one unique bacteria genus. The PPT had seven individual taxa in fungal genera communities, while the Mono cropping system had six. Push–pull and Mono cropping systems shared 11 overlapping fungal genera. Between sample and soil type, and cropping systems interaction, PPT soil and PPT maize-root had eight and one unique genus, respectively, compared to Mono soil and Mono maize-root, which had five and one genus (Fig. 5). In terms of study locations, Vihiga had six unique fungal genera compared to Bungoma and Siaya.

#### Alpha diversity of soil and maize-root microbiomes in push–pull and maize-monoculture cropping system fields

**Fig. 6:**
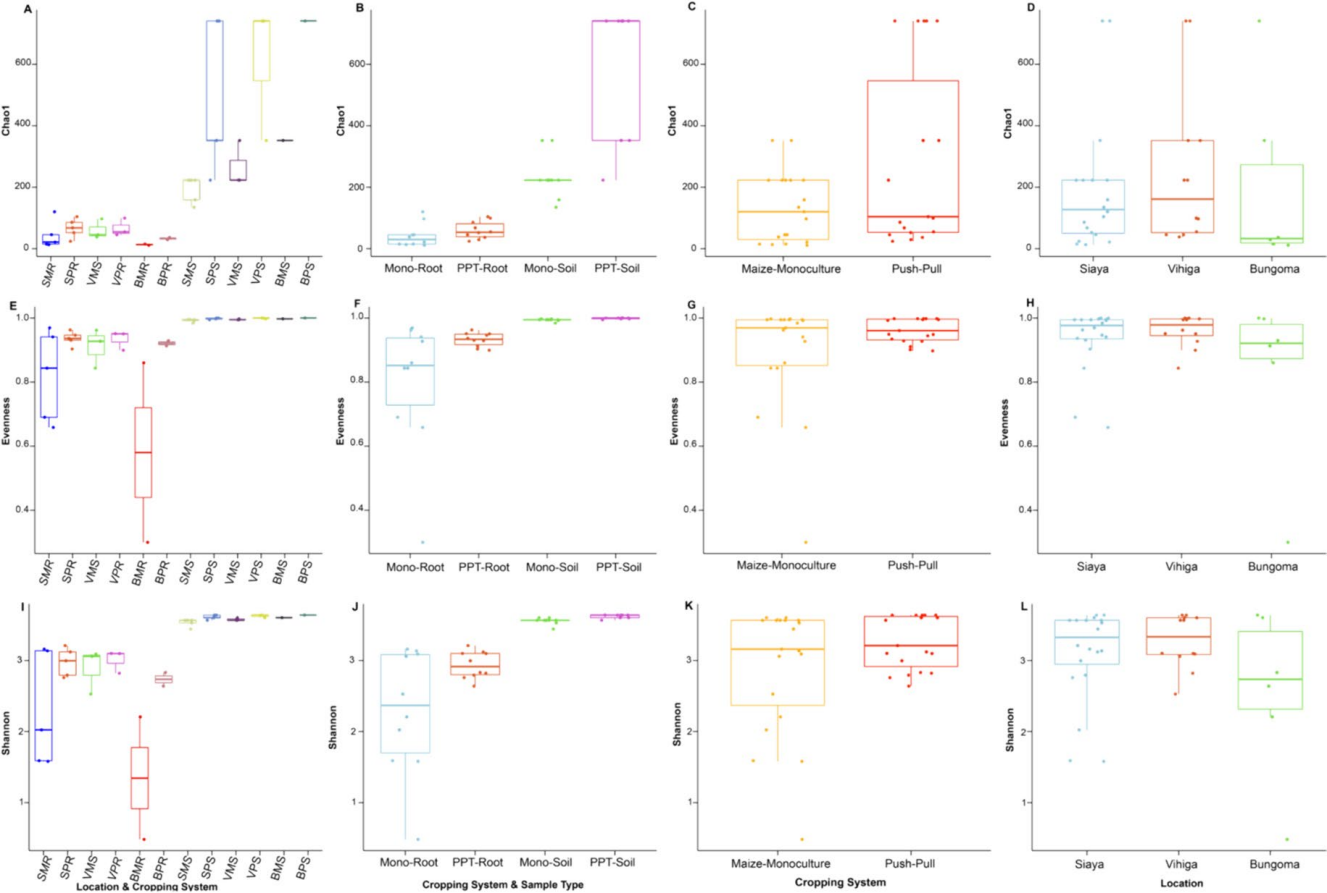
Alpha diversity of bacterial communities at the genus level; **A, E, I** Location, sample type, and cropping systems; BPR, Bungoma push–pull root; BPS, Bungoma push–pull soil; BMR, Bungoma maize-monoculture root; BMS, Bungoma maize-monoculture soil; SPR, Siaya push–pull maize-root; SPS, Siaya push–pull soil; SMR, Siaya maize-monoculture root; SMS, Siaya monoculture soil; VPR, Vihiga push–pull root; VPS, Vihiga push–pull soil; VMR, Vihiga maize-monoculture root; VMS, Vihiga maize-monoculture soil; **B, F, J** Cropping systems and sample type; PPT-soil, push–pull soil; Mono-soil, maize-monoculture soil; PPT-root, push–pull root; Mono-root, maize-monoculture root; **C, G, K** Cropping systems; Push–Pull (soil + maize-root); Maize-monoculture (soil + maize-root); **D, H, L** Locations

There was no significant difference in bacterial communities between PPT (soil + maize-root) and Mono (soil + maize-root) cropping systems in richness (*Chao1 estimator*, *P* = 0.820) and evenness (*Shannon index*, *P* = 0.390) (Fig. 6). However, a significant difference was found in richness and evenness between cropping system, sample, and soil type interactions (*Chao1 estimator*, PPT-soil vs Mono-root *P* < 0.001; PPT-soil vs PPT-root *P* < 0.001; Mono-soil vs Mono-root *P* < 0.001; *Shannon index*, PPT-soil vs Mono-root *P* < 0.001; PPT-soil vs PPT-root *P* < 0.001; Mono-soil vs Mono-root *P* < 0.001). There was no significant difference in richness and evenness between PPT and Mono-soil (*Chao1 estimator*, *P* = 0.730; *Shannon index*, *P* = 0.140). We observed no significant difference between the studied locations in richness and evenness (*Chao1 estimator*, Bungoma vs Vihiga *P* = 0.340; Bungoma vs Siaya *P* = 0.490; Vihiga vs Siaya *P* = 0.690; *Shannon index*, Bungoma vs Vihiga *P* = 0.180; Bungoma vs Siaya *P* = 0.570; Vihiga vs Siaya *P* = 0.370).

**Fig. 7:**
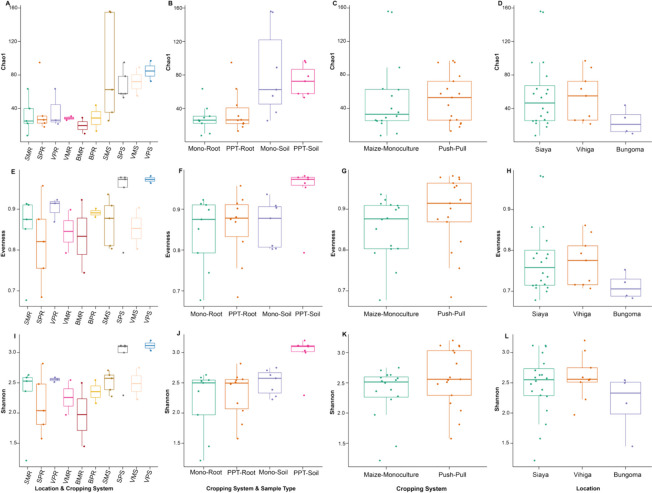
Alpha diversity of fungal communities at the genus level; **A, E, I** Location, sample type, and cropping systems; BPR, Bungoma push–pull maize-root; BPS, Bungoma push–pull soil; BMR, Bungoma maize-monoculture root; BMS, Bungoma maize-monoculture soil; SPR, Siaya push–pull maize-root; SPS, Siaya push–pull soil; SMR, Siaya maize-monoculture root; SMS, Siaya maize-monoculture soil; VPR, Vihiga push–pull maize-root; VPS, Vihiga push–pull soil; VMR, Vihiga maize-monoculture root; VMS, Vihiga maize-monoculture soil; **B, F, J** Cropping systems and sample type; PPT-soil, push–pull soil; Mono-soil, maize-monoculture soil; PPT-root, push–pull root; Mono-root, maize-monoculture root; **C, G, K** Cropping systems; Push–Pull (soil + maize-root); Maize-Monoculture (soil + maize-root); **D, H, L** Locations

The fungal communities did not differ in richness (*Chao1 estimator*, *P* = 0.930) and evenness (*Shannon index*, *P* = 0.710) between PPT (soil + maize-root) and Mono (soil + maize-root) cropping systems (Fig. 7). However, there was a significant difference in the fungal community in richness and evenness between cropping systems, sample, and soil type interaction (*Chao1 estimator*, PPT-soil vs PPT-root *P* < 0.001; PPT-soil vs Mono-root *P* < 0.001; Mono-soil vs Mono-root *P* < 0.001; *Shannon index*, PPT-soil vs PPT-root *P* < 0.001; PPT-soil vs Mono-root *P* < 0.002; Mono-soil vs Mono-root *P* < 0.003). There was no significant difference between the cropping system, sample, and soil type interaction in richness (*Chao1 estimator*, PPT-soil vs Mono-soil *P* = 0.160 and *Shannon index*, PPT-soil vs Mono-soil *P* = 0.073). There was no significant effect in richness between studied locations (*Chao1 estimator*, Bungoma vs Siaya *P* = 0.180; Vihiga vs Siaya *P* = 0.560; except Bungoma vs Vihiga *P* = 0.034). There was a significant effect in evenness between studied locations (*Shannon index*, Bungoma vs Vihiga *P* = 0.056; Bungoma vs Siaya *P* = 0.050; except Vihiga vs Siaya *P* = 0.940).

#### Beta diversity and the influence of push–pull and maize-monoculture cropping systems on soil and maize-root microbial communities

**Fig. 8:**
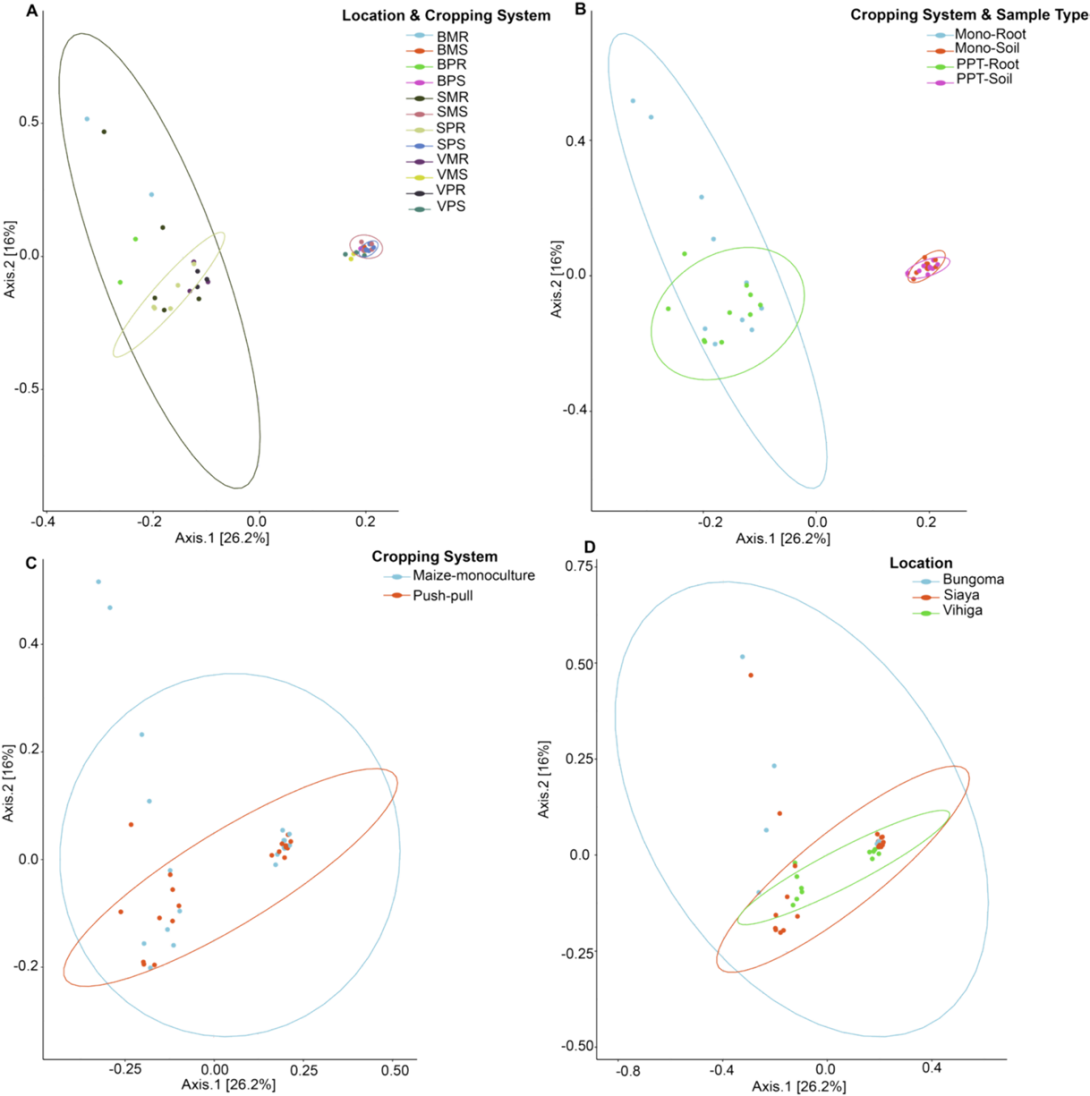
Beta diversity of bacterial communities at the genus level; **A** Location, sample type, and cropping systems; BPR, Bungoma push–pull root; BPS, Bungoma push–pull soil; BMR, Bungoma maize-monoculture root; BMS, Bungoma maize-monoculture soil; SPR, Siaya push–pull root; SPS, Siaya push–pull soil; SMR, Siaya maize-monoculture root; SMS, Siaya maize-monoculture soil; VPR, Vihiga push–pull root; VPS, Vihiga push–pull soil; VMR, Vihiga maize-monoculture root; VMS, Vihiga maize-monoculture soil; **B** Cropping systems and sample type; PPT-soil, push–pull soil; Mono-soil, maize-monoculture soil; PPT-root, push–pull root; Mono-root, maize-monoculture root; **C** Cropping systems; Push–Pull (soil + maize-root); Maize-Monoculture (soil + maize-root); **D** Locations

**Fig. 9:**
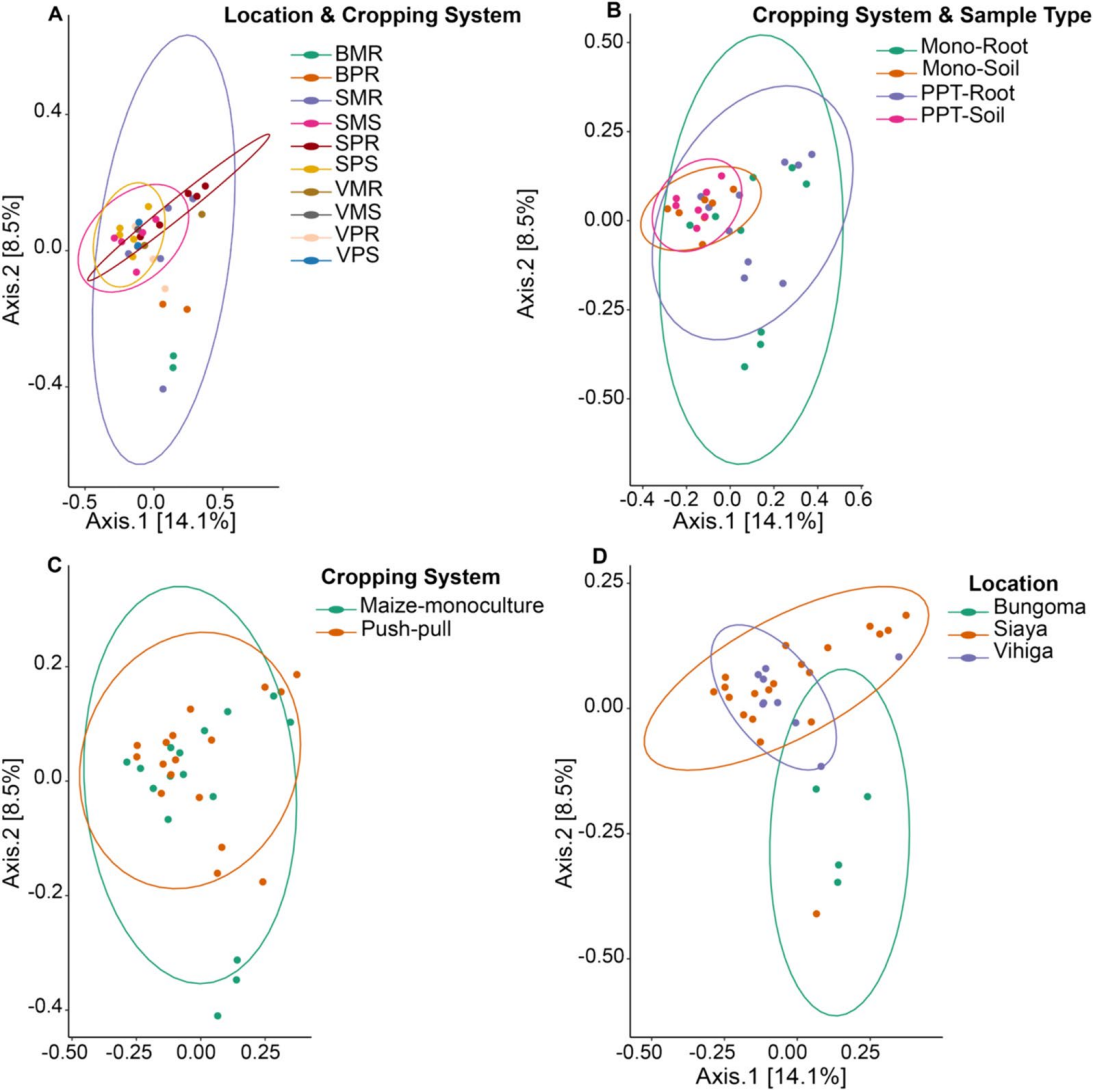
Beta diversity of fungal communities at the genus level; **A** Location, sample type, and cropping system; BPR, Bungoma push–pull root; BPS, Bungoma push–pull soil; BMR, Bungoma maize-monoculture root; BMS, Bungoma maize-monoculture soil; SPR, Siaya push–pull root; SPS, Siaya push–pull soil; SMR, Siaya maize-monoculture root; SMS, Siaya maize-monoculture soil; VPR, Vihiga push–pull maize-root; VPS, Vihiga push–pull soil; VMR, Vihiga maize-monoculture root; VMS, Vihiga maize-monoculture soil; **B** Cropping systems and sample type; PPT-soil, push–pull soil; Mono-soil, maize-monoculture soil; PPT-root, push–pull root; Mono-root, maize-monoculture root; **C** Cropping systems; Push–Pull (soil + maize-root); Maize-Monoculture (soil + maize-root); **D** Locations

The β-diversity of fungal and bacterial communities was compared between PPT and Mono cropping systems through visualization and quantification of soil and maize-root microbiomes community (bacterial and fungal) clustered by cropping systems (Figs. 8 and 9). Betadisper analyses revealed no significant differences in bacterial and fungal community structures across different cropping systems (*P* = 0.211 and *P* = 0.966, respectively), an indication of true biological difference within-group dispersion. Betadisper analyses of microbial communities across different studied locations revealed significant difference only in fungal communities (bacteria,* P* = 0.581; fungi,* P* = 0.001, respectively). However, when comparing sample types, betadisper results demonstrated significant differences in bacterial and fungal community dispersion (*P* = 0.001; *P* = 0.024, respectively). PERMANOVA analyses found significant differences in microbial composition between the different sample types and studied locations that were conditioned by the two cropping systems (sample types and cropping systems of bacteria, *R*^2^ = 0.337, *P* < 0.001 and fungi *R*^2^ = 0.160, *P* < 0.001; locations, sample types and cropping systems of bacteria *R*^2^ = 0.524, *P* < 0.001 and fungi *R*^2^ = 0.352, *P* < 0.001; study locations of bacteria *R*^2^ = 0.071, *P* > 0.05 and fungi *R*^2^ = 0.991, *P* < 0.001; Additional file 1: Tables S9 (bacteria) and S10 (fungi)). However, there was no significant difference in the PPT and Mono cropping systems (bacteria *R*^2^ = 0.018, *P* > 0.05 and fungi *R*^2^ = 0.032, *P* > 0.05; Additional file 1: Tables S9 (bacteria) and S10 (fungi)). The soil and maize-root bacterial communities of the sample type and cropping systems were distinctly separated along axis 1, and we observed a subtle clustering by PPT and Mono cropping systems and sample type along axis 2. The soil bacterial communities tended to group themselves through the intensity of sample type interaction (Fig. 8). A comparable trend was observed in the soil and maize-root fungal communities (Fig. 9).

#### Differential expression of bacterial protein functions in soil and maize-root

**Fig. 10:**
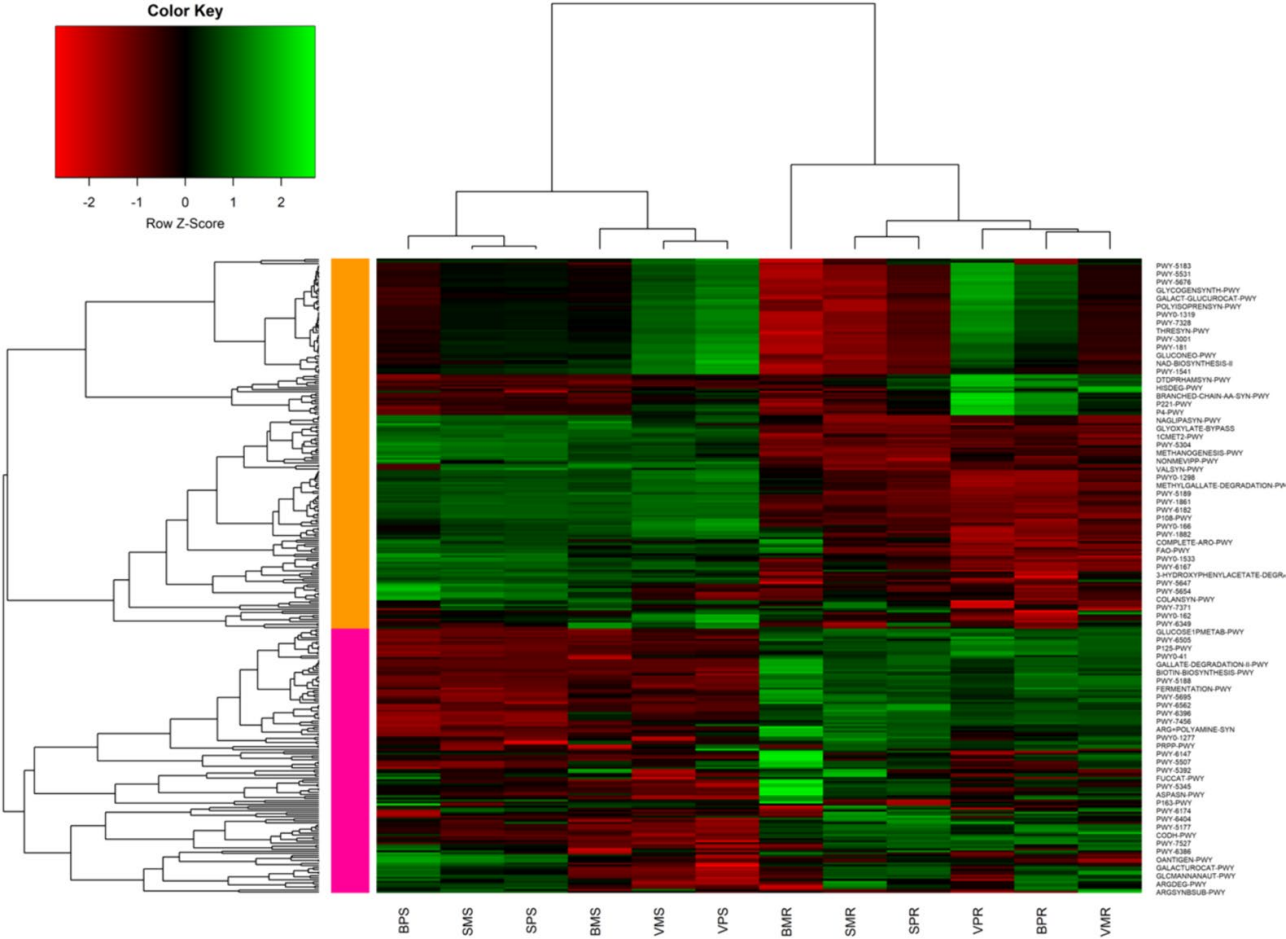
The normalized relative abundance of predicted functional categories in bacterial communities across various cropping systems, sample types, and locations: BPS, Bungoma push–pull soil; SMS, Siaya maize-monoculture soil; SPS, Siaya push–pull soil; BMS, Bungoma maize-monoculture soil; VMS, Vihiga maize-monoculture soil; VPS, Vihiga push–pull soil; BMR, Bungoma maize-monoculture root; SMR, Siaya maize-monoculture root; SPR, Siaya push–pull maize-root; VPR, Vihiga push–pull maize-root; BPR, Bungoma push–pull maize-root; VMR, Vihiga maize-monoculture root. Pathways correlation is indicated within the range of -2, and + 2 in different cropping systems. The colors red, black, and green represent negative, zero, and positive correlations

The prediction of sequences associated with significant functional metabolic pathways in PPT and Mono cropping systems across different sample types and locations revealed the following six most abundant pathways: PWY-5695 (inosine 5-phosphate degradation), theocat-PWY (superpathway of L-threonine metabolism), gallate-degradation-I-PWY (gallate degradation II), P221-PWY (octane oxidation), 3-hydroxyphenyllacetate-degradation, and biotin-biosynthesis PWY. Based on hierarchical clustering, the different cropping systems, sample types, and locations were grouped into two main clades. In the first clade, Bungoma push–pull soil (BPS), Siaya maize-monoculture soil (SMS), and Siaya push–pull soil (SPS) clustered together. Similarly, Bungoma maize-monoculture soil (BMS), Vihiga maize-monoculture soil (VMS), and Vihiga push–pull soil (VPS) formed another cluster. In the second clade, Bungoma maize-monoculture root (BMR) clustered separately, while Siaya maize-monoculture root (SMR) and Siaya push–pull root (SPR) clustered together. Vihiga push–pull root (VPR), Bungoma push–pull root (BPR), and Vihiga maize-monoculture root (VMR) were also grouped (Fig. 10).

**Fig. 11:**
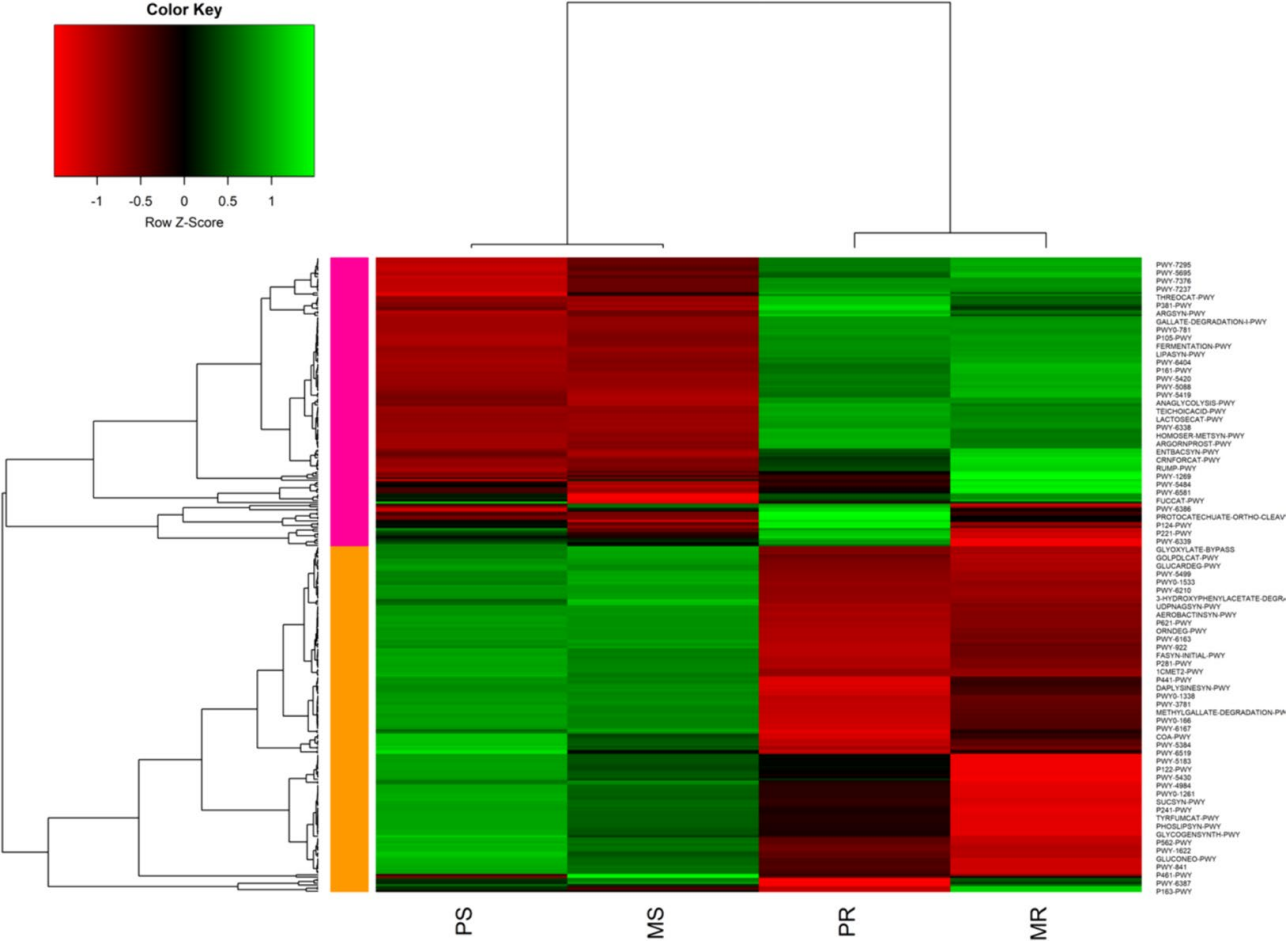
Normalized relative abundance of predicted functional categories in bacterial communities across various cropping systems and sample types: PS, push–pull soil; MS, maize-monoculture soil; PR, push–pull maize-root; and MR, maize-monoculture root. Pathways correlation is indicated within the range of -2, and + 2 in different cropping systems. The colors red, black, and green represent negative, zero, and positive correlations

Based on the prediction of sequences associated with major functional metabolic pathways, the ten most abundant pathways were identified in the two cropping systems: PWY-5695 (inosine 5'-phosphate degradation), theocat-PWY (superpathway of L-threonine metabolism), gallate-degradation-I-PWY (gallate degradation II), lipasyn-PWY (phospholipases), P161-PWY (acetylene degradation (anaerobic)), PWY-5088 (L-glutamate degradation VIII), lactosecat-PWY (lactose degradation I), homoser-metsyn-PWR (L-methionine biosynthesis I), PWY-6386 (syringate degradation), and P221-PWY (octane oxidation). Regarding the cropping systems, two hierarchical clustering clades were observed. The first clade consisted of push–pull soil (PS) and maize-monoculture soil (MS), while the second clade consisted of push–pull root (PR) and maize-monoculture root (MR) (Fig. 11).

## Discussion

The use of push–pull cropping system by small-scale farmers positively impacted soil physicochemical properties, as well as soil and maize-root microbial communities. The positive association of PPT was demonstrated by the enhancement of soil OC, pH, P, N, and B. Additionally, the presence of ecologically important belowground microbial groups involved in soil fertility improvement, decomposition, siderophore production, high carbon sequestration, nutrient cycling, and plant protection in comparison to the Mono cropping system further supported this positive association. These findings can be linked to agroecosystem functions and other ecosystem services, including soil health and maize yield. This section explores the contribution of PPT on belowground ecosystem services provision including soil physicochemical properties, microbiome relative abundance and diversity, shedding light on the system’s role and function in promoting sustainable maize production.

### Effect of push–pull cropping system on soil physicochemical properties

Previously, multiple cropping systems have been shown to influence soil characteristics such as pH, organic carbon, and nitrogen compared to Mono [35, 53]. In this study, we found higher levels of pH, OC, N, and P in soil conditioned by PPT in comparison to the Mono cropping system. A pH below 5.5 can negatively affect plants and pose a significantly threat to the agroecosystem [54]. Given that the pH in the PPT was higher than in Mono, we infer that PPT positively contributes to enhancing soil physicochemical properties and the overall soil health [3, 22]. Frac et al. [55] reported that biotic and abiotic factors, such as soil pH, structure, and nutrient levels, influence the diversity and activity of soil microbes. Our findings regarding the possible influence of cropping systems on both above and belowground abiotic and biotic factors align with the predictions by Drinkwater et al. [3, 5, 54, 56, 57] that crop diversification significantly impacts belowground microbiomes, plant, and soil health, as well as overall productivity.

### Impact of push–pull cropping system on soil and maize-root bacterial community

There is growing evidence suggesting that plant diversification impact belowground microbiomes [6, 12, 58]. This study found that PPT cropping systems led to a higher diversity of soil bacterial communities compared to that of the Mono cropping system soil. Notably, the high relative abundances of beneficial bacterial genera such as *Sphingomonas*, *Bacillus*, *Enterobacter*, *RB41*, *Herbaspirillum*, *Nocardioides*, *Mitsuaria*, *Gaiella*, *Nitrospira*, *Burkholderia-Caballeronia-Paraburkholderia*, *Dyella*, *Enterobacter*, and *Conexibacter* in both the soil and maize-root of PPT systems indicate that PPT favors the proliferation of beneficial bacteria, which improve crop performance and possibly contribute to pest management. *Herbaspirillum* is a nitrogen-fixing endophytic bacterium that colonizes plant roots and has been shown to positively impact plant growth, and crop yield [59]. Additionally, *Bacillus* and *Enterobacter* bacterial genera are potential biofertilizer agents due to their ability to solubilize inorganic phosphate, fix nitrogen, act as biological control agents, carry out bioremediation, and promote plant growth [60, 61]. *Sphingomonas*, *Gaiella*, and *Dyella* play a vital role in promoting plant growth by producing phytohormones and/or inducing changes in phytohormone signalling through volatile organic compound (VOCs), decomposition of lignocellulose, bioremediation of hydrocarbon-contaminated soil, and nutrient cycling in agroecosystem fields [62–64]. Additionally, *Sphingomonas* possesses distinctive capabilities, including the degradation of persistent contaminants, acting as bacterial antagonists to phytopathogenic fungi, and secreting highly beneficial gellan exopolysaccharides [65]. *RB41* plays a critical role in regulating the soil carbon cycle and is involved in processing the metabolism of both organic and inorganic nitrogen sources [30, 35, 61]. Furthermore, according to Huang et al. [66], *Burkholderia* and *Mitsuaria* genera have a beneficial impact on drought resistance in plants. These bacteria accomplish this by reducing the levels of ethylene, a plant hormone, and producing 1-aminocyclopropane-1-carboxylic acid. Brewer et al. [67] stated that *Candidatus Udaeobacter* contributes to global hydrogen cycling by utilizing H_2_. Lazcano et al. [68] found that *Nocardioides* spp. can act as biocontrol agents for bacterial leaf spots and promote plant growth.

Soil and maize-root from PPT had a greater relative abundance of *Streptomyces* and *Stenotrophomonas*, which possess broad biotechnological potential, such as the ability to promote plant growth, production of bioactive secondary metabolites, VOCs, and are promising candidates for biocontrol of phytopathogenic microbes [69]. These characteristics may be attributed to their multiplication rate, ability to produce antibiotics and siderophores, controlled gene expression quorum detection, and synthesis of lipase, chitinase, cellulases, phytohormones, β-1,3-glucanase, and amino acids [70]. *Streptomyces* spp. can colonize plant root surfaces, survive in various soil types, and produce spores that allow them to persist in extreme conditions. *Stenotrophomonas* is a potential biocontrol agent against *Ralstonia* [71, 72]. The presence of these bacterial genera in PPT soils and maize-roots implies that PPT positively influences belowground microbial populations compared to that of Mono. Similar findings have been observed in various other cropping systems, including long-term intercropping systems, push–pull experimental plots, multiple cropping systems, crop rotation, and cover cropping [7, 8, 31, 58]. *Nitrospira*, which are capable of carrying out nitrification through oxidation of ammonia involving a single organism as opposed to other nitrifying bacteria, which require two different organisms to complete the process [3, 7, 73] were enriched in PPT. These findings imply that PPT influences maize-root microbial populations compared to Mono-root and affects maize-root and soil microbial communities. We also found that the presence of companion crops in a push–pull cropping system had a greater impact on PPT maize-root microbiota such as *Streptomyces*, *Herbasoirillum*, *Stenotrophomonas*, *Sphingomonas*, *Allorhizobium-Neorhizobium-Pararhizobium-Rhizobium*, and *Dyella* compared to Mono-root cropping system. The presence of these beneficial bacteria in the push–pull maize-root may positively contribute to an increase in nitrogen nutrients, carbon sequestration, and biocontrol agent against plant pathogens. This, in turn, can results in improved plant growth due to plant growth-promoting rhizobacterial (PGPR) and siderophores availability, which facilitates iron content in soil and plants from the PPT field. This may lead to higher crop yields in PPT fields compared to that of Mono fields. To better understand the role of different bacterial and fungal species, including those within the same genus, in this cropping system, it is necessary to perform species-level characterization. The finding that *Bryobacter,* a disease-causing bacterial genus, was more abundant in Mono than in the PPT cropping system shows that Mono cropping systems potentially predispose crops to disease-causing agents.

The high relative abundance of beneficial bacterial species, including *Rhizobium phaseoli*, *Bacillus flexus*, *Bradyrhizobium elkanii*, *Paraburkholderia vietnamiensis*, *Dyella marensis*, *Enterobacter hormaechei*, *Herbaspirillum seropedicae*, *Pseudomonas nitroreducens*, *Ralstonia pickettii*, *Sphingomonas paucimobilis*, *Stenotrophomonas maltophilia*, and *Variovorax paradoxus*, in both the soil and maize-roots within the push–pull cropping systems indicates that this system promotes the proliferation of bacteria that enhance crop performance, improve soil health, water purification, and plant growth, and potentially contribute to insect-pests and disease management. Interestingly, *Bacillus flexus* possesses the ability to solubilize tricalcium phosphate and hydroxyapatite, making it valuable for biodegradation processes [74, 75]. *Bradyrhizobium elkanii* produces rhizobitoxine, which acts as a defense mechanism against stress-induced ethylene and plays a significant role in nitrogen fixation [76]. *Variovorax paradoxus* and *Pseudomonas aeruginosa* can degrade and/or metabolize N-acyl-homoserine lactones (AHLs) as a carbon source [77]. Chen et al. [78] demonstrated the importance of the complete ethylene signal transduction pathway in enhancing *Arabidopsis thaliana* growth through the PGPR, *Variovorax paradoxus*, underscoring the significance of ethylene signalling PGPR activity. *Stenotrophomonas maltophilia* contributes to bioremediation and nitrogen fixation processes. Interestingly, it contributes to the sulfur cycle and promotes plant growth and health in ecosystems [72, 79]. *Sphingomonas paucimobilis* enhances antioxidant activity, promotes plant growth, and exhibits biodegradation capabilities [80, 81]. *Ralstonia pickettii* demonstrates biodegradative abilities through siderophore production, while *Pseudomonas nitroreducens* produce biosurfactants and solubilizes phosphate [82–84]. *Herbaspirillum seropedicae*, an endophytic diazotrophic PGPR, colonizes various crops (rice, maize, sorghum, and sugarcane) and exhibits beneficial traits such as solubilization of minerals, production of phytohormones, and atmospheric nitrogen fixation [85, 86]. *Enterobacter hormaechei* has been identified as a potassium solubilizing microbe, showing potential for plant growth and controlling harmful algal blooms [87–90]. *Dyella marensis* produces biosurfactants and siderophores, while *Paraburkholderia vietnamiensis* and *Rhizobium phaseoli* have shown promise as nitrogen-fixing fertilizers for plant growth [91–93].

### Impact of push–pull cropping system on soil and maize-root mycobiome

Push–pull cropping system decreased the number of harmful fungal genera. Contrarily, it increased the presence and relative abundance of beneficial belowground fungal genera, such as *Mortieralla*, *Exophiala*, *Paraboeremia*, *Bionectria*, *Clitopilus*, *Marasmius*, *Pyrenochaetopsis*, and *Trichoderma* compared to the Mono cropping system. These findings align with previous studies which have demonstrated crop diversification enhance beneficial fungi with a positive impact on agroecosystem productivity [3, 6, 94, 95]. For example, *Mortierella* spp. has been shown to solubilize phosphate, improve nutrient uptake, and influence soil microbiota, synthesize phytohormones that support plant growth and defense mechanisms [74]. Enriched in PPT, *Mortierella* and *Pyrenochaetopsis* spp. are important indicators of soil-root microbiome continuum, enhancing crop yield, disease resistance, and salinity tolerance in tomatoes [90]. *Exophiala* spp. which was enriched in PPT, has been observed to produce phytohormones and enzymes, promoting plant shoot growth under drought and salinity conditions [15]. *Paraboeremia* spp. has been demonstrated to increase plant biomass and glycyrrhizin content in Liquorice plants [96], and it can parasitize eggs of the rice root-knot nematode, *Meloidogyne graminicola*, in in-vitro assays [27, 97]. *Bionectria* spp. has been shown to decompose plant debris, improve soil health, and act as biological control agents against insect-pests [98]. The volatile antimicrobial compounds produced by this fungus suppress plant pathogens and could be used as an effective biofumigant [99, 100]. *Clitopilus* spp. produces pleuromutilin, a biologically active compound with potent antimicrobial activity and the ability to increase plant growth through facilitative potassium uptake [101, 102]. *Trichoderma* spp. found enriched in PPT, is associated with colonizing the rhizoplane, rhizosphere, and plant roots, and produces metabolites with antimicrobial (volatile and non-volatile compounds, cellulose/lignin/cell wall degrading enzymes and antibiotics) and biostimulating properties (phytohormones and phytoregulators) [98, 103]. This fungus has direct and indirect biocontrol potential against soil phytopathogens, increases nutrient solubility, and contributes to plant protection, crop yield, and biofertilization production [104, 105]. Fungal spp. belonging to *Ramicandelaber* and *Robillarda* have been reported as decomposers, with *Robillarda* producing β-1,3/1,4-glucans that contribute to disease resistance in plants [106, 107]. While harmful fungal genera such as *Aspergillus*, *Gibberalla*, *Neocosmopora*, and *Curvularia* were found to be more enriched in the Mono cropping system compared to that of PPT, it is important to note that not all species within these genera are harmful. Some species within these genera also exist as endophytes. However, some produce toxins; for example, Zearalenone, an estrogenic mycotoxin that is produced by *Gibberella* spp. causes *Gibberella* ear rot (GER) in crops like maize, oats, wheat, sorghum, rice, and barley [33, 94, 108]. *Neocosmospora*, identified as a phytopathogen causing stem rot, adversely affects potato growth and yields, leading to economic losses due to stunted growth, leaf yellowing, and grayish-black stems [109]. Fungal spp. belonging to *Curvularia*, poses a threat to cereal crops, causing economically burdensome *Curvularia* leaf spots in maize [110, 111]. Similarly, mycotoxin producing species like *Aspergillus*, infect various fruits, cereal, and vegetable plants, causing several disorders, reducing seed germination, and impairing root and shoot elongation [32, 112].

#### Diversity of soil and maize-root microbiome in push–pull and maize-monoculture cropping systems

While annual legume intercropping may temporarily affect belowground microbiome profiles, the impact of perennial companion intercrop, such as *Desmodium* spp. is expected to be stronger and more resilient, contributing to increased soil and maize-root microbial diversity [3, 7, 113]. Hence, we argue that the higher beta diversity of microbial communities in long-term push–pull compared to Mono cropping systems, in both soil and maize-root bacterial and fungal populations, could result from the baseline differences between the two cropping systems. These differences include factors such as a companion crop like *Desmodium* spp. which likely contributes to a more diverse and resilient microbial community. This underscores the potential benefits of incorporating companion crops in agroecosystems to enhance belowground microbial communities. Similar trends have been reported in other studies investigating cereal and legume intercropping systems, such as wheat-soybean, millet-mung bean, and maize/wheat-faba bean; push–pull on-station experimental plots [7, 11, 57, 114]. Crop diversification, aimed at improving food security, soil fertility, and/or controlling insect-pests through push–pull strategies or maize-legume intercropping systems [3, 5, 10, 17], has demonstrated additional benefits, including the suppression of parasitic weeds like *Striga* spp. increased soil nitrogen and carbon content, and reduction of mycotoxin incidence in maize [21–23]. The current study contributes to these benefits by highlighting diversification of soil and maize-root microbial communities, particularly emphasizing a significant positive shift in ecologically important bacterial and fungal genera. Ecosystem diversity is generally recognized to enhance stability, resilience, and productivity, primarily due to resource complementarity and functional redundancy. These findings underscore the importance of promoting crop diversification, like push–pull, to cultivate a balanced and resilient beneficial microbiome in agricultural ecosystems, mitigating risks associated with Mono cropping.

### Microbiome functional protein pathways

The study focused on differential expression of microbial protein function in push–pull cropping systems. It identified crucial pathways, such as inosine 5'-phosphate degradation (PWY-5695), theocat-PWY (L-threonine metabolism), gallate-degradation-I-PWY (gallate degradation II), lipasyn-PWY (phospholipases), P161-PWY (acetylene degradation, anaerobic), PWY-5088 (L-glutamate degradation VIII), lactosecat-PWY (lactose degradation I), homoser-metsy n-PWR (L-methionine biosynthesis I), PWY-6386 (syringate degradation), catechol degradation II (meta-cleavage pathway), and P221-PWY (octane oxidation) most of which were enhanced in PPT. These pathways contribute to soil–plant biochemical processes, plant growth, nitrogen fixation, stress, and disease resistance, climate change effects, and root architecture modulation [59, 115–117].

Nitrogenase is crucial for converting atmospheric nitrogen into ammonia, meeting the plant's nitrogen requirements, and promoting plant growth. Inosine 5'-phosphate degradation pathways are involved in nitrogen fixation, where bacteria generate ammonia, essential for purine synthesis during nitrogen fixation in plant roots [118, 119]. Zahid et al. [60] reported that aerobic bacteria utilize acetylene for plant growth and nitrogen fixation. Gallate degradation II pathways contribute to the breakdown of plant lignin and tannins in the carbon cycle [117]. Phospholipases act as crucial second messengers in plant signal transduction during growth, development, and stress responses [120]. The meta-cleavage pathway is essential for the degradation of aromatic compounds and has been observed in bacterial genera like *Azotobacter*, *Ralstonia*, and *Pseudomonas* [115, 116]. *Pseudomonas simiae* WCS417r induce resistance against pathogens [121, 122], while *Bacillus subtilis* S499 provides ISR-mediated protection to tomato plants against *Botrytis cinerea* [123]*.* L-glutamate degradation VIII pathway plays a role in nutrient foraging and shaping root architecture in soil environments, like the plant growth regulator auxin (indole-3-acetic acid, IAA) [123, 124]. Notably, L-methionine biosynthesis positively influences maize and tomato plant growth [125, 126]. The syringate degradation pathway enables microbes to utilize lignin-derived compounds. Lignin is broken down into biaryl and monoaryl compounds such as Biphenyl, ferulate, vanillate, and syringate. Microbes like *Sphingomonas* spp. SYK6 can use guaiacyl and syringyl moieties derived from lignin to degrade them into vanillate and syringate [127]. Further research is necessary to investigate the influence of soil microorganisms on soil physicochemical properties, plant mineral nutrients, and bacterial protein activities, especially in perennial intercropping scenarios.

## Conclusion

The study demonstrated that the PPT cropping system greatly influenced soil characteristics such as pH, P, N, and soil organic carbon content compared to the Mono cropping system. It further revealed that the PPT cropping system shifts belowground soil and maize-root microbiome composition compared to the Mono cropping system. The microbial communities enriched in both soil and maize-root by the PPT belong to genera and species associated with essential ecosystem services such as soil fertility enhancement, organic matter decomposition, carbon sequestration, plant protection, and human safety. This enrichment contributes to the diversification of ecosystem services provided by the cropping system in farmer fields, enhancing the system’s resilience and functional redundancy. Further research is needed to assess which specific soil and maize-root microorganisms are strongly impacted by the cropping system, their role in aboveground tri-trophic interactions, and their influence on enzymatic activity and nutrient accessibility in farmer fields where this cropping system is practiced. Additionally, exploring the impact of *Desmodium* root exudates on the belowground microbiome and their interactions with maize-root and soil organisms are crucial for gaining a comprehensive understanding of the ecological dynamics within the cropping system.

### Supplementary Information

**Supplementary Material 1.**

**Supplementary Material 2.**

**Supplementary Material 3.**

**Supplementary Material 4.**

## Data Availability

The unprocessed sequencing datasets generated during the current study have been deposited in GenBank, NCBI under BioProject PRJNA1015669. The 16S (V1-V2, V3-V4) metagenome data were registered as Biosamples SAMN37384180 – SAMN37384217 and the sequences assigned SRA accessions SRR26087688 – SRR26087719. The ITS (ITS1-ITS2) metagenome data were registered as Biosamples SAMN37384218 – SAMN37384250 and the sequences assigned SRA accessions SRR26087651 – SRR26087687. We also provide the R scripts for data analysis along with all the necessary input files as Additional file 2A and B. The data for soil physicochemical properties and GPS coordinates were provided as Additional files 3 and 4.
