# Supplementary material for: Long-term push–pull cropping system shifts soil and maize-root microbiome diversity paving way to resilient farming system
Source: BMC Microbiol. 2024 Mar 18;24:92. doi: 10.1186/s12866-024-03238-z (PMC10946131; doi:10.1186/s12866-024-03238-z)
Supplement: Supplementary file 1 — Supplementary Material 1. [file 12866_2024_3238_MOESM1_ESM.doc]

**Additional File**

**Long-term Push-pull Cropping System Shifts Soil and Maize-root Microbiome Diversity Paving Way To Resilient Farming System**

Abdul A. Jalloh^1,2^, Fathiya Khamis^1^, Abdullahi Ahmed Yusuf ^2,3^, Sevgan Subramanian^1^, Daniel Munyao Mutyambai^1,4*^

*1. International Centre of Insect Physiology and Ecology, P.O. Box 30772-00100 Nairobi, Kenya.*

*2. Department of Zoology and Entomology, University of Pretoria, Pretoria, Private Bag x20 Hatfield, South Africa.*

*3. Forestry and Agricultural Biotechnology Institute, University of Pretoria, Pretoria, Private Bag x20 Hatfield, South Africa.*

*4.* *Department of Life Sciences, South Eastern Kenya University, P.O. Box 170-90200 Kitui, Kenya.*

**Corresponding author:* [*dmutyambai@icipe.org*](mailto:dmutyambai@icipe.org)

**Supplementary Results**

**Taxonomic profiles of belowground bacterial and fungal communities**

We found the 30 most abundant bacterial genera communities in push-pull technology (PPT) and maize-monoculture (Mono) cropping systems. The highest order of bacteria relative abundance in PPT (soil and maize-root) compared to that of Mono (soil and maize-root) cropping system was as follows Burkholderia-Caballeronia-Paraburkholderia>, Enterobacter>, Sphingomonas>, Candidatus Udaeobacter>, Bacillus>, Ralstonia>, Sphingobium>, Herbaspirillum>, Gaiella>, Candidatus. Solibacter>, Dyella>, RB41>, Stenotrophomonas>, Streptomyces>, Chryseobacterium>, Mycobacterium>, Anaeromyxobacter>, Nitrospira>, Nocardioides>, Mitsuaria> and MND1 **(Table S1).** The Mono cropping system had Pseudomonas>, Bryobacter>, Allorhizobium-Neorhizobium-Pararhizobium-Rhizobium>, Bradyrhizobium>, Conexibacter>, Acidothermus>, Flavobacterium> and Pantoea as the most relatively abundant genera compared to PPT. However, the interaction between sample type, PPT, and Mono cropping system (PPT soil and PPT maize-root, and Mono soil and Mono maize-root) had varying effects on the impact of the bacterial genera communities (**Table S2**). In terms of study counties, the most enriched bacterial genera in Bungoma in the order of relatively abundant were Pseudomonas>, Allorhizobium-Neorhizobium-Pararhizobium-Rhizobium>, Flavobacterium> and Nocardioides compared to that of Siaya and Vihiga. Streptomyces>, Burkholderia-Caballeronia-Paraburkholderia>, Bacillus>, Conexibacter>, Sphingobium>, Dyella>, cidothermus>, Chryseobacterium>, Gaiella>, Herbaspirillum>, Mitsuaria>, Mycobacterium>, Pantoea>, Ralstonia> and RB41 were the most abundant genera in Siaya compared to the other two counties. Candidatus Udaeobacter>, Bradyrhizobium>, Enterobacter>, Sphingomonas>, Bryobacter>, Candidatus Solibacter>, Anaeromyxobacter>, Ellin6067>, MND1>, Nitrospira> and Stenotrophomonas were more abundant in Vihiga compared to that of Bungoma and Siaya counties (**Table S3)**. However, PPT positively impacts bacterial genera between the cropping system and study locations in different sample types. Burkholderia-Caballeronia-Paraburkholderia were more relatively abundant in Siaya PPT maize-root compared to the other cropping system, sample types, and study locations. Acidothermus> and Pantoea were more abundant in Siaya Mono maize-root, and Conexibacter were more enriched in Siaya Mono soil **(Table S4)**. Allorhizobium-Neorhizobium-Pararhizobium-Rhizobium were more relatively abundant in Vihiga PPT maize-root compared to the other cropping system, sample type, and study location. Ellin6067>, Enterobacter>, MND1>, Nitrospira> and Streptomyces were more relatively abundant in Vihiga PPT soil. Bradyrhizobium>, Herbaspirillum> and Stenotrophomonas were more dominant in Vihiga Mono maize-root. Arthrobacter>, Candidatus Udaeobacter> and Nitrospira were more enriched in Vihiga Mono soil. Dyella and Ralstonia were more relatively abundant in Bungoma PPT maize-root compared to the other cropping system, sample types, and study locations. Gaiella>, Mycobacterium>, Nocardioides>, Bacillus> and Sphingobium were more relatively abundant in Bungoma PPT soil. Anaeromyxobacter> Bryobacter>, Candidatus Solibacter>, Flavobacterium>, Pseudolabrys>, RB41> and Sphingomonas were more relatively abundant in Bungoma Mono soil. Pseudomonas were more dominant in Bungoma Mono maize-root compared to the other cropping system, sample type, and study locations.

We found the 30 most abundant fungal genera communities in PPT and Mono cropping systems (**Tables S5, S6, S7, and S8).**

**Supplementary Tables**

**Table S1:** Relative abundance (%) of bacterial genera communities in push-pull (soil + maize-root) and maize-monoculture (soil + maize-root) cropping systems. Bacterial genera with relative abundances < 1 were grouped as 'Other'.

| **S/No.** | **Genus** | **Percentage relative abundance** | |
| --- | --- | --- | --- |
|  |  | **Cropping systems** | |
|  |  | **PPT** | **Mono** |
| 1 | *Burkholderia-Caballeronia-Paraburkholderia* | 13.50 | 9.60 |
| 2 | *Enterobacter* | 8.40 | 3.40 |
| 3 | *Sphingomonas* | 5.30 | 4.00 |
| 4 | *Candidatus Udaeobacter* | 5.20 | 4.20 |
| 5 | *Pseudomonas* | 4.90 | 23.50 |
| 6 | *Bacillus* | 4.60 | 4.00 |
| 7 | *Ralstonia* | 3.40 | 1.60 |
| 8 | *Bryobacter* | 2.00 | 2.10 |
| 9 | *Sphingobium* | 2.10 | 2.00 |
| 10 | *Herbaspirillum* | 2.00 | 1.70 |
| 11 | *Allorhizobium-Neorhizobium-Pararhizobium-Rhizobium* | 1.90 | 2.10 |
| 12 | *Bradyrhizobium* | 1.80 | 2.90 |
| 13 | *Gaiella* | 1.80 | 1.40 |
| 14 | *Candidatus Solibacter* | 1.60 | 1.20 |
| 15 | *Dyella* | 1.30 | 0.90 |
| 16 | *RB41* | 1.30 | 1.00 |
| 17 | *Stenotrophomonas* | 1.20 | 0.40 |
| 18 | *Streptomyces* | 1.20 | 0.80 |
| 19 | *Chryseobacterium* | 1.10 | 0.40 |
| 20 | *Mycobacterium* | 1.10 | 1.00 |
| 21 | *Anaeromyxobacter* | 1.00 | 0.70 |
| 22 | *Nitrospira* | 1.00 | 0.70 |
| 23 | *Nocardioides* | 1.00 | 0.50 |
| 24 | *Conexibacter* | 0.90 | 1.70 |
| 25 | *Mitsuaria* | 0.90 | 0.60 |
| 26 | *MND1* | 0.90 | 0.60 |
| 27 | *Arthrobacter* | 0.70 | 0.70 |
| 28 | *Acidothermus* | 0.60 | 0.80 |
| 29 | *Flavobacterium* | 0.60 | 1.20 |
| 30 | *Pantoea* | 0.30 | 1.50 |
|  | Others | 26.50 | 22.80 |
|  | **Total** | **100** | **100** |

**Table S2:** Relative abundance (%) of bacterial genera communities in push-pull and maize-monoculture cropping system interaction and sample type. Bacterial genera with relative abundances < 1 were grouped as 'Other'.

| **S/No.** | **Genus** | **Percentage relative abundance** | | | |
| --- | --- | --- | --- | --- | --- |
|  |  | **Cropping systems** | | | |
|  |  | **PPT** | | **Mono** | |
|  |  | **Soil** | **Maize-root** | **Soil** | **Maize-root** |
| 1 | *Acidothermus* | 1.10 | 0.00 | 2.20 | 0.00 |
| 2 | *Allorhizobium-Neorhizobium-Pararhizobium-Rhizobium* | 0.30 | 3.50 | 0.30 | 3.10 |
| 3 | *Anaeromyxobacter* | 2.00 | 0.00 | 1.90 | 0.00 |
| 4 | *Arthrobacter* | 1.30 | 0.10 | 1.70 | 0.00 |
| 5 | *Bacillus* | 7.80 | 1.60 | 6.40 | 2.50 |
| 6 | *Bradyrhizobium* | 2.40 | 1.10 | 2.40 | 3.20 |
| 7 | *Bryobacter* | 4.30 | 0.00 | 5.20 | 0.00 |
| 8 | *Burkholderia-Caballeronia-Paraburkholderia* | 0.60 | 25.80 | 0.50 | 15.00 |
| 9 | *Candidatus Solibacter* | 3.30 | 0.00 | 3.20 | 0.00 |
| 10 | *Candidatus Udaeobacter* | 10.60 | 0.10 | 11.20 | 0.10 |
| 11 | *Conexibacter* | 1.80 | 0.00 | 4.60 | 0.00 |
| 12 | *Dyella* | 0.00 | 2.40 | 0.10 | 1.40 |
| 13 | *Ellin6067* | 1.60 | 0.00 | 1.50 | 0.00 |
| 14 | *Enterobacter* | 0.50 | 15.80 | 0.30 | 5.30 |
| 15 | *Flavobacterium* | 0.90 | 0.30 | 1.60 | 0.90 |
| 16 | *Gaiella* | 3.60 | 0.00 | 3.80 | 0.00 |
| 17 | *Herbaspirillum* | 0.10 | 3.80 | 0.00 | 2.60 |
| 18 | *Mitsuaria* | 0.00 | 1.70 | 0.00 | 1.00 |
| 19 | *MND1* | 1.90 | 0.00 | 1.60 | 0.00 |
| 20 | *Mycobacterium* | 2.10 | 0.10 | 2.50 | 0.10 |
| 21 | *Nitrospira* | 2.10 | 0.00 | 2.00 | 0.00 |
| 22 | *Nocardioides* | 2.00 | 0.00 | 1.40 | 0.00 |
| 23 | *Pantoea* | 0.00 | 0.50 | 0.30 | 2.30 |
| 24 | *Pseudomonas* | 0.90 | 8.70 | 0.90 | 37.10 |
| 25 | *Ralstonia* | 0.10 | 6.40 | 0.10 | 2.50 |
| 26 | *RB41* | 2.70 | 0.00 | 2.70 | 0.00 |
| 27 | *Sphingobium* | 0.10 | 4.00 | 0.00 | 3.20 |
| 28 | *Sphingomonas* | 8.30 | 2.50 | 8.10 | 1.50 |
| 29 | *Stenotrophomonas* | 0.00 | 2.30 | 0.00 | 0.70 |
| 30 | *Streptomyces* | 1.60 | 0.90 | 1.20 | 0.50 |
|  | Others | 35.90 | 18.30 | 32.30 | 16.90 |
|  | **Total** | **100** | **100** | **100** | **100** |

**Table S3:** Relative abundance (%) of bacterial genera communities in push-pull and maize-monoculture cropping systems based on study locations (counties). Bacterial genera with relative abundances < 1 were grouped as 'Other'

| **S/No.** | **Genus** | **Percentage relative abundance** | | |
| --- | --- | --- | --- | --- |
|  |  | **Study counties** | | |
|  |  | **Bungoma** | **Siaya** | **Vihiga** |
| 1 | *Acidothermus* | 0.40 | 1.00 | 0.40 |
| 2 | *Allorhizobium-Neorhizobium-Pararhizobium-Rhizobium* | 2.80 | 2.00 | 1.50 |
| 3 | *Anaeromyxobacter* | 0.40 | 0.90 | 1.00 |
| 4 | *Bacillus* | 2.40 | 6.60 | 1.20 |
| 5 | *Bradyrhizobium* | 1.30 | 1.70 | 4.70 |
| 6 | *Bryobacter* | 1.10 | 2.10 | 2.60 |
| 7 | *Burkholderia-Caballeronia-Paraburkholderia* | 4.20 | 16.40 | 6.80 |
| 8 | *Candidatus Solibacter* | 0.80 | 1.20 | 2.20 |
| 9 | *Candidatus Udaeobacter* | 2.70 | 3.70 | 8.40 |
| 10 | *Chryseobacterium* | 0.50 | 0.90 | 0.50 |
| 11 | *Conexibacter* | 0.50 | 2.00 | 0.60 |
| 12 | *Dyella* | 0.90 | 1.50 | 0.30 |
| 13 | *Ellin6067* | 0.40 | 0.60 | 1.00 |
| 14 | *Enterobacter* | 3.00 | 7.10 | 4.80 |
| 15 | *Flavobacterium* | 2.20 | 0.20 | 1.30 |
| 16 | *Gaiella* | 1.00 | 1.80 | 1.50 |
| 17 | *Herbaspirillum* | 1.70 | 1.90 | 1.70 |
| 18 | *Mitsuaria* | 0.10 | 1.00 | 0.90 |
| 19 | *MND1* | 0.40 | 0.60 | 1.30 |
| 20 | *Mycobacterium* | 0.80 | 1.20 | 0.90 |
| 21 | *Nitrospira* | 0.60 | 0.60 | 1.70 |
| 22 | *Nocardioides* | 0.90 | 0.60 | 0.80 |
| 23 | *Pantoea* | 0.50 | 1.20 | 0.70 |
| 24 | *Pseudomonas* | 48.00 | 6.50 | 6.00 |
| 25 | *Ralstonia* | 2.10 | 3.30 | 0.90 |
| 26 | *RB41* | 0.30 | 0.70 | 2.70 |
| 27 | *Sphingobium* | 1.40 | 2.70 | 1.40 |
| 28 | *Sphingomonas* | 2.20 | 4.10 | 7.40 |
| 29 | *Stenotrophomonas* | 0.20 | 0.40 | 1.90 |
| 30 | *Streptomyces* | 0.60 | 0.90 | 1.30 |
|  | Others | 15.90 | 24.40 | 31.70 |
|  | **Total** | **100** | **100** | **100** |

**Table S4:** Relative abundance of (%) bacterial genera communities in push-pull and maize-monoculture cropping systems between sample type and study location. Bacterial genera with relative abundances < 1 were grouped as 'Other'

| **S/no.** | **Genus** | **Percentage relative abundance** | | | | | | | | | | | |
| --- | --- | --- | --- | --- | --- | --- | --- | --- | --- | --- | --- | --- | --- |
|  |  | **Cropping systems, locations and sample types interaction** | | | | | | | | | | | |
|  |  | **SPR** | **SPS** | **SMR** | **SMS** | **VPR** | **VPS** | **VMR** | **VMS** | **BPR** | **BPS** | **BMR** | **BMS** |
| 1 | *Acidothermus* | 0.00 | 1.80 | 0.00 | 3.50 | 0.10 | 0.50 | 0.00 | 0.70 | 0.00 | 0.70 | 0.00 | 1.70 |
| 2 | *Allorhizobium-Neorhizobium-Pararhizobium-Rhizobium* | 3.40 | 0.30 | 2.80 | 0.20 | 5.20 | 0.20 | 2.30 | 0.40 | 2.50 | 0.60 | 4.00 | 0.50 |
| 3 | *Anaeromyxobacter* | 0.00 | 2.30 | 0.00 | 2.00 | 0.00 | 2.00 | 0.00 | 1.40 | 0.00 | 0.10 | 0.00 | 2.40 |
| 4 | *Arthrobacter* | 0.00 | 1.60 | 0.00 | 1.90 | 0.10 | 0.80 | 0.00 | 2.10 | 0.20 | 1.30 | 0.00 | 0.50 |
| 5 | *Bacillus* | 1.80 | 12.20 | 5.00 | 10.60 | 2.10 | 0.50 | 0.50 | 2.10 | 0.40 | 13.50 | 0.20 | 3.70 |
| 6 | *Bradyrhizobium* | 1.10 | 2.40 | 1.30 | 2.40 | 1.10 | 2.20 | 12.50 | 2.20 | 1.30 | 3.60 | 0.30 | 3.00 |
| 7 | *Bryobacter* | 0.00 | 4.90 | 0.00 | 5.70 | 0.10 | 4.20 | 0.00 | 4.50 | 0.00 | 1.80 | 0.00 | 5.40 |
| 8 | *Burkholderia-Caballeronia-Paraburkholderia* | 28.70 | 0.60 | 25.00 | 0.60 | 25.50 | 0.80 | 11.40 | 0.40 | 14.50 | 0.30 | 2.50 | 0.60 |
| 9 | *Candidatus Solibacter* | 0.00 | 3.20 | 0.00 | 3.00 | 0.00 | 4.20 | 0.00 | 2.90 | 0.00 | 1.00 | 0.00 | 4.50 |
| 10 | *Candidatus Udaeobacter* | 0.10 | 9.20 | 0.00 | 9.10 | 0.20 | 13.70 | 0.10 | 13.80 | 0.00 | 6.70 | 0.10 | 11.60 |
| 11 | *Conexibacter* | 0.00 | 2.80 | 0.00 | 7.80 | 0.00 | 0.50 | 0.00 | 1.60 | 0.00 | 1.50 | 0.00 | 1.90 |
| 12 | *Dyella* | 2.20 | 0.00 | 2.60 | 0.10 | 1.30 | 0.10 | 0.50 | 0.00 | 4.60 | 0.00 | 0.10 | 0.00 |
| 13 | *Ellin6067* | 0.00 | 1.50 | 0.00 | 1.50 | 0.00 | 1.90 | 0.00 | 1.30 | 0.00 | 0.90 | 0.00 | 1.70 |
| 14 | *Enterobacter* | 17.30 | 0.00 | 6.40 | 0.10 | 12.80 | 1.30 | 9.50 | 0.60 | 12.60 | 0.00 | 1.10 | 0.10 |
| 15 | *Flavobacterium* | 0.10 | 0.20 | 0.40 | 0.00 | 0.90 | 2.00 | 1.10 | 0.80 | 0.50 | 0.40 | 1.50 | 7.90 |
| 16 | *Gaiella* | 0.00 | 4.40 | 0.00 | 4.80 | 0.10 | 2.10 | 0.00 | 3.00 | 0.00 | 5.40 | 0.00 | 2.30 |
| 17 | *Herbaspirillum* | 4.50 | 0.00 | 1.80 | 0.00 | 1.40 | 0.20 | 5.70 | 0.00 | 3.10 | 0.00 | 2.10 | 0.10 |
| 18 | *MND1* | 0.00 | 1.80 | 0.00 | 1.50 | 0.00 | 2.50 | 0.00 | 1.70 | 0.00 | 0.30 | 0.00 | 2.10 |
| 19 | *Mycobacterium* | 0.10 | 2.40 | 0.20 | 3.40 | 0.00 | 1.20 | 0.10 | 1.60 | 0.00 | 3.90 | 0.00 | 1.90 |
| 20 | *Nitrospira* | 0.00 | 1.60 | 0.00 | 1.30 | 0.00 | 2.70 | 0.00 | 2.70 | 0.00 | 2.20 | 0.00 | 2.20 |
| 21 | *Nocardioides* | 0.00 | 1.80 | 0.10 | 1.30 | 0.00 | 1.00 | 0.00 | 1.80 | 0.00 | 6.00 | 0.00 | 0.90 |
| 22 | *Pantoea* | 0.50 | 0.00 | 3.50 | 0.00 | 0.10 | 0.00 | 2.80 | 0.00 | 0.70 | 0.00 | 0.20 | 1.80 |
| 23 | *Pseudolabrys* | 0.10 | 0.80 | 0.00 | 0.50 | 0.00 | 1.80 | 0.00 | 0.70 | 0.00 | 2.20 | 0.00 | 2.90 |
| 24 | *Pseudomonas* | 4.80 | 0.20 | 16.40 | 0.10 | 5.10 | 2.00 | 16.50 | 1.70 | 27.60 | 0.30 | 79.70 | 1.70 |
| 25 | *Ralstonia* | 6.50 | 0.10 | 4.20 | 0.10 | 3.80 | 0.10 | 1.10 | 0.00 | 8.50 | 0.00 | 0.80 | 0.10 |
| 26 | *RB41* | 0.00 | 2.20 | 0.00 | 1.40 | 0.00 | 3.70 | 0.00 | 5.30 | 0.00 | 1.10 | 0.00 | 1.00 |
| 27 | *Sphingobium* | 4.20 | 0.00 | 4.60 | 0.00 | 1.60 | 0.20 | 4.20 | 0.00 | 5.40 | 0.10 | 0.60 | 0.10 |
| 28 | *Sphingomonas* | 2.80 | 7.40 | 1.90 | 5.90 | 1.10 | 10.10 | 1.50 | 12.80 | 2.40 | 5.60 | 0.90 | 4.00 |
| 29 | *Stenotrophomonas* | 0.80 | 0.00 | 0.70 | 0.00 | 10.30 | 0.00 | 1.80 | 0.00 | 0.70 | 0.00 | 0.10 | 0.00 |
| 30 | *Streptomyces* | 0.30 | 1.90 | 0.60 | 1.40 | 3.60 | 0.90 | 1.00 | 1.00 | 0.80 | 2.50 | 0.10 | 0.90 |
|  | Others | 20.60 | 32.30 | 22.30 | 29.70 | 23.50 | 36.60 | 27.20 | 32.90 | 14.20 | 38.10 | 5.80 | 32.50 |
|  | **Total** | **100** | **100** | **100** | **100** | **100** | **100** | **100** | **100** | **100** | **100** | **100** | **100** |

**Keys:** SPR, Siaya push-pull maize-root; SPS, Siaya push-pull soil; SMR, SPS, Siaya monoculture maize-root; SMS, Siaya maize- monoculture soil; VPR, Vihiga push-pull maize-root; Vihiga push-pull soil; Vihiga maize- monoculture maize-root; Vihiga maize- monoculture soil; Bungoma push-pull maize-root; Bungoma push-pull soil; Bungoma maize- monoculture maize-root; Bungoma maize- monoculture soil

**Table S5:** Relative abundance of (%) fungal genera communities in push-pull (soil + maize-root) and maize-monoculture (soil + maize-root) cropping systems. Fungal genera with relative abundances < 1 were grouped as 'Other'.

| **S/No.** | **Genus** | **Percentage relative abundance** | |
| --- | --- | --- | --- |
|  |  | **Cropping systems** | |
|  |  | **PPT** | **Mono** |
| 1 | *Arachnion* | 2.40 | 0.00 |
| 2 | *Aspergillus* | 0.40 | 0.90 |
| 3 | *Bionectria* | 2.20 | 1.20 |
| 4 | *Chloridium* | 0.50 | 0.50 |
| 5 | *Clitopilus* | 1.70 | 0.20 |
| 6 | *Condenascus* | 1.10 | 1.10 |
| 7 | *Curvularia* | 0.60 | 2.10 |
| 8 | *Ectophoma* | 1.60 | 0.10 |
| 9 | *Exophiala* | 11.90 | 7.80 |
| 10 | *Gibberella* | 6.20 | 8.40 |
| 11 | *Lectera* | 1.00 | 0.20 |
| 12 | *Marasmius* | 1.70 | 0.20 |
| 13 | *Mortierella* | 17.00 | 14.70 |
| 14 | *Neocosmospora* | 1.00 | 1.20 |
| 15 | *Paraboeremia* | 8.90 | 3.50 |
| 16 | *Parafabraea* | 0.80 | 1.60 |
| 17 | *Penicillium* | 1.90 | 4.10 |
| 18 | *Poaceascoma* | 0.40 | 0.90 |
| 19 | *Psathyrella* | 0.20 | 1.40 |
| 20 | *Purpureocillium* | 0.60 | 0.90 |
| 21 | *Pyrenochaetopsis* | 0.90 | 0.50 |
| 22 | *Ramicandelaber* | 0.70 | 0.40 |
| 23 | *Remotididymella* | 1.00 | 0.20 |
| 24 | *Robillarda* | 0.80 | 0.60 |
| 25 | *Similiphoma* | 1.60 | 19.30 |
| 26 | *Spiromyces* | 12.10 | 10.00 |
| 27 | *Talaromyces* | 3.40 | 4.40 |
| 28 | *Trichoderma* | 0.90 | 0.50 |
| 29 | *Wallemia* | 1.30 | 0.10 |
| 30 | *Xepicula* | 0.70 | 0.70 |
|  | Others | 14.70 | 12.20 |
|  | **Total** | **100** | **100** |

**Table S6:** Relative abundance of (%) fungal genera communities in push-pull and maize-monoculture cropping system interaction and sample type. Fungal genera with relative abundances lower than 1 were grouped as 'Other'.

| **S/No.** | **Genus** | **Percentage relative abundance** | | | |
| --- | --- | --- | --- | --- | --- |
|  |  | **Cropping systems** | | | |
|  |  | **PPT** | | **Mono** | |
|  |  | **Soil** | **Maize-root** | **Soil** | **Maize-root** |
| 1 | *Arachnion* | 3.60 | 0.00 | 0.10 | 0.00 |
| 2 | *Aspergillus* | 0.50 | 0.10 | 1.70 | 0.00 |
| 3 | *Bionectria* | 3.10 | 0.30 | 2.10 | 0.00 |
| 4 | *Chloridium* | 0.70 | 0.00 | 1.00 | 0.00 |
| 5 | *Clitopilus* | 2.50 | 0.00 | 0.40 | 0.00 |
| 6 | *Condenascus* | 1.50 | 0.30 | 1.90 | 0.10 |
| 7 | *Curvularia* | 0.10 | 1.50 | 1.10 | 3.30 |
| 8 | *Ectophoma* | 2.30 | 0.10 | 0.20 | 0.00 |
| 9 | *Exophiala* | 3.70 | 28.40 | 0.50 | 16.70 |
| 10 | *Gibberella* | 3.40 | 11.70 | 9.50 | 7.10 |
| 11 | *Lectera* | 1.50 | 0.00 | 0.40 | 0.00 |
| 12 | *Marasmius* | 0.30 | 4.40 | 0.30 | 0.00 |
| 13 | *Mortierella* | 24.30 | 2.40 | 24.80 | 2.30 |
| 14 | *Neocosmospora* | 1.20 | 0.60 | 1.70 | 0.60 |
| 15 | *Paraboeremia* | 1.60 | 23.60 | 0.60 | 7.10 |
| 16 | *Parafabraea* | 1.10 | 0.20 | 2.80 | 0.20 |
| 17 | *Penicillium* | 2.70 | 0.40 | 6.20 | 1.70 |
| 18 | *Poaceascoma* | 0.00 | 1.10 | 0.10 | 1.90 |
| 19 | *Psathyrella* | 0.30 | 0.00 | 0.10 | 2.90 |
| 20 | *Purpureocillium* | 0.90 | 0.00 | 1.60 | 0.00 |
| 21 | *Pyrenochaetopsis* | 1.10 | 0.40 | 0.50 | 0.50 |
| 22 | *Ramicandelaber* | 1.00 | 0.00 | 0.80 | 0.00 |
| 23 | *Remotididymella* | 1.40 | 0.00 | 0.30 | 0.00 |
| 24 | *Robillarda* | 1.10 | 0.20 | 0.90 | 0.20 |
| 25 | *Similiphoma* | 0.10 | 4.60 | 0.00 | 43.00 |
| 26 | *Spiromyces* | 16.00 | 4.40 | 15.80 | 2.80 |
| 27 | *Talaromyces* | 4.50 | 1.20 | 7.70 | 0.20 |
| 28 | *Trichoderma* | 1.20 | 0.30 | 0.80 | 0.10 |
| 29 | *Wallemia* | 1.90 | 0.00 | 0.10 | 0.00 |
| 30 | *Xepicula* | 1.00 | 0.20 | 1.30 | 0.00 |
|  | Others | 15.30 | 13.50 | 14.80 | 9.00 |
|  | **Total** | **100** | **100** | **100** | **100** |

**Table S7: Relative abundance of (%) fungal genera communities in push-pull and maize-monoculture cropping systems based on study locations (counties). Fungal genera with relative abundances < 1 were grouped as 'Other'.**

| **S/No** | **Genus** | **Percentage relative abundance** | | |
| --- | --- | --- | --- | --- |
|  |  | **Study counties** | | |
|  |  | **Bungoma** | **Siaya** | **Vihiga** |
| 1 | *Arachnion* | 0.00 | 1.60 | 0.00 |
| 2 | *Aspergillus* | 0.00 | 1.00 | 0.20 |
| 3 | *Bionectria* | 0.00 | 1.90 | 1.70 |
| 4 | *Chloridium* | 0.00 | 0.50 | 0.80 |
| 5 | *Clitopilus* | 0.00 | 1.30 | 0.10 |
| 6 | *Condenascus* | 0.00 | 1.40 | 0.80 |
| 7 | *Curvularia* | 5.40 | 0.90 | 1.10 |
| 8 | *Delfinachytrium* | 0.00 | 0.10 | 1.20 |
| 9 | *Ectophoma* | 0.10 | 0.90 | 0.70 |
| 10 | *Exophiala* | 6.00 | 10.30 | 9.00 |
| 11 | *Gibberella* | 8.00 | 6.70 | 9.00 |
| 12 | *Marasmius* | 0.00 | 0.10 | 3.30 |
| 13 | *Mortierella* | 0.30 | 17.00 | 19.60 |
| 14 | *Neocosmospora* | 0.10 | 1.20 | 1.30 |
| 15 | *Paraboeremia* | 3.10 | 5.90 | 7.00 |
| 16 | *Parafabraea* | 0.10 | 1.00 | 2.70 |
| 17 | *Penicillium* | 0.10 | 4.00 | 2.50 |
| 18 | *Poaceascoma* | 0.50 | 0.80 | 0.40 |
| 19 | *Psathyrella* | 0.00 | 1.30 | 0.00 |
| 20 | *Purpureocillium* | 0.00 | 1.10 | 0.20 |
| 21 | *Pyrenochaetopsis* | 0.00 | 0.90 | 0.60 |
| 22 | *Remotididymella* | 0.00 | 0.60 | 0.50 |
| 23 | *Robillarda* | 0.00 | 0.80 | 0.80 |
| 24 | *Sarocladium* | 2.00 | 0.10 | 0.00 |
| 25 | *Similiphoma* | 65.70 | 5.50 | 1.20 |
| 26 | *Spiromyces* | 2.40 | 11.20 | 15.90 |
| 27 | *Talaromyces* | 0.10 | 4.90 | 2.90 |
| 28 | *Trichoderma* | 0.10 | 0.70 | 0.70 |
| 29 | *Wallemia* | 0.00 | 0.70 | 0.60 |
| 30 | *Xepicula* | 0.00 | 0.70 | 1.30 |
|  | **Total** | **100** | **100** | **100** |

**Table S8:** Relative abundance of (%) fungal genera communities in push-pull and maize-monoculture cropping systems based on study locations (counties). Fungal genera with relative abundances < 1 were grouped as 'Other.'

| **S/No** | **Genus** | **Percentage relative abundance** | | | | | | | | | |
| --- | --- | --- | --- | --- | --- | --- | --- | --- | --- | --- | --- |
|  |  | **Cropping systems, locations, and sample types interaction** | | | | | | | | | |
|  |  | **SPR** | **SPS** | **SMR** | **SMS** | **VPR** | **VPS** | **VMR** | **VMS** | **BPR** | **BMR** |
| 1 | *Arachnion* | 0.00 | 4.60 | 0.00 | 0.10 | 0.00 | 0.00 | 0.00 | 0.00 | 0.00 | 0.00 |
| 2 | *Aspergillus* | 0.10 | 0.60 | 0.00 | 2.20 | 0.30 | 0.40 | 0.00 | 0.20 | 0.00 | 0.00 |
| 3 | *Bionectria* | 0.40 | 2.90 | 0.00 | 2.30 | 0.30 | 3.70 | 0.10 | 1.50 | 0.00 | 0.00 |
| 4 | *Chloridium* | 0.00 | 0.50 | 0.00 | 1.00 | 0.10 | 1.30 | 0.00 | 1.10 | 0.00 | 0.00 |
| 5 | *Clitopilus* | 0.00 | 3.30 | 0.00 | 0.50 | 0.00 | 0.00 | 0.00 | 0.20 | 0.00 | 0.00 |
| 6 | *Condenascus* | 0.10 | 1.50 | 0.10 | 2.40 | 0.60 | 1.50 | 0.50 | 0.40 | 0.10 | 0.00 |
| 7 | *Curvularia* | 1.00 | 0.20 | 1.10 | 1.30 | 2.90 | 0.10 | 2.20 | 0.50 | 0.60 | 6.50 |
| 8 | *Delfinachytrium* | 0.00 | 0.10 | 0.00 | 0.30 | 0.00 | 0.70 | 0.00 | 2.90 | 0.00 | 0.00 |
| 9 | *Ectophoma* | 0.00 | 2.30 | 0.00 | 0.30 | 0.00 | 2.20 | 0.10 | 0.00 | 0.80 | 0.00 |
| 10 | *Exophiala* | 42.40 | 2.80 | 22.20 | 0.30 | 7.50 | 6.80 | 30.80 | 0.90 | 14.30 | 4.10 |
| 11 | *Gibberella* | 4.00 | 3.20 | 10.60 | 9.30 | 13.30 | 4.20 | 10.30 | 10.30 | 37.30 | 1.40 |
| 12 | *Lectera* | 0.00 | 1.90 | 0.00 | 0.50 | 0.00 | 0.00 | 0.00 | 0.40 | 0.00 | 0.00 |
| 13 | *Marasmius* | 0.00 | 0.20 | 0.00 | 0.10 | 15.70 | 0.70 | 0.00 | 0.80 | 0.00 | 0.00 |
| 14 | *Mortierella* | 2.80 | 26.60 | 0.10 | 21.70 | 2.50 | 16.40 | 14.20 | 33.80 | 1.00 | 0.10 |
| 15 | *Neocosmospora* | 0.40 | 1.10 | 0.90 | 1.70 | 1.00 | 1.30 | 0.60 | 1.70 | 0.30 | 0.10 |
| 16 | *Paraboeremia* | 31.10 | 1.70 | 7.10 | 0.50 | 18.40 | 1.30 | 18.00 | 0.70 | 5.30 | 2.50 |
| 17 | *Parafabraea* | 0.30 | 1.00 | 0.40 | 1.70 | 0.00 | 1.60 | 0.10 | 6.00 | 0.00 | 0.10 |
| 18 | *Penicillium* | 0.50 | 2.00 | 1.80 | 8.30 | 0.40 | 5.10 | 5.20 | 0.10 | 0.10 | 0.00 |
| 19 | *Poaceascoma* | 0.40 | 0.00 | 3.90 | 0.10 | 1.60 | 0.00 | 0.40 | 0.00 | 2.70 | 0.00 |
| 20 | *Psathyrella* | 0.00 | 0.30 | 6.20 | 0.10 | 0.00 | 0.10 | 0.00 | 0.00 | 0.00 | 0.00 |
| 21 | *Purpureocillium* | 0.00 | 1.10 | 0.00 | 2.00 | 0.00 | 0.20 | 0.00 | 0.30 | 0.00 | 0.00 |
| 22 | *Pyrenochaetopsis* | 0.30 | 1.20 | 1.20 | 0.50 | 1.00 | 0.80 | 0.00 | 0.40 | 0.00 | 0.00 |
| 23 | *Remotididymella* | 0.10 | 1.60 | 0.00 | 0.10 | 0.00 | 0.90 | 0.30 | 0.60 | 0.00 | 0.00 |
| 24 | *Robillarda* | 0.10 | 1.00 | 0.20 | 1.10 | 0.30 | 1.80 | 0.80 | 0.40 | 0.10 | 0.00 |
| 25 | *Similiphoma* | 0.10 | 0.20 | 30.00 | 0.00 | 5.70 | 0.00 | 1.00 | 0.00 | 19.80 | 76.40 |
| 26 | *Spiromyces* | 2.60 | 14.50 | 4.50 | 13.50 | 6.00 | 20.90 | 2.60 | 22.40 | 8.40 | 0.90 |
| 27 | *Talaromyces* | 1.60 | 3.20 | 0.50 | 10.20 | 0.60 | 8.90 | 0.00 | 0.70 | 0.50 | 0.00 |
| 28 | *Trichoderma* | 0.10 | 1.10 | 0.00 | 0.90 | 1.00 | 1.20 | 0.10 | 0.40 | 0.00 | 0.10 |
| 29 | *Wallemia* | 0.00 | 1.90 | 0.00 | 0.10 | 0.00 | 2.10 | 0.00 | 0.10 | 0.00 | 0.00 |
| 30 | *Xepicula* | 0.00 | 1.10 | 0.00 | 0.80 | 0.70 | 0.50 | 0.10 | 2.90 | 0.00 | 0.00 |
|  | Others | 11.60 | 16.30 | 9.00 | 16.10 | 20.00 | 15.40 | 12.80 | 10.30 | 8.60 | 7.60 |
|  | **Total** | **100** | **100** | **100** | **100** | **100** | **100** | **100** | **100** | **100** | **100** |

**Table S9:** Results of PERMANOVA testing the effects of sample type, location, cropping system, and their interaction on bacterial communities on soil and maize-root from smallholder fields. Significant effects are indicated in bold at *P* < 0.05.

| **Factors** | ***df*** | **Sum of Sqs** | ***R^2^*** | ***F* value** | ***P* value** |
| --- | --- | --- | --- | --- | --- |
| Sample type and cropping systems | 3 | 4.417 | 0.337 | 5.764 | **0.0001** |
| Residual | 34 | 8.686 | 0.663 | - | - |
| Cropping systems | 1 | 0.236 | 0.018 | 0.661 | 0.8526 |
| Residual | 36 | 12.866 | 0.982 | - | - |
| Study locations | 2 | 0.933 | 0.071 | 1.342 | 0.1236 |
| Residual | 35 | 12.169 | 0.929 | - | - |
| Locations, sample type and cropping systems | 11 | 6.863 | 0.524 | 2.599 | **0.0001** |
| Residual | 26 | 6.240 | 0.476 | - | - |

**Table S10:** Results of PERMANOVA testing the effects of sample type, location, cropping systems, and their interaction on fungal communities on soil and maize-root from smallholder fields. Significant effects are indicated in bold at *P* < 0.05.

| **Factor** | ***df*** | **Sum of Sqs** | ***R^2^*** | ***F* value** | ***P* value** |
| --- | --- | --- | --- | --- | --- |
| Sample type and cropping systems | 3 | 2.015 | 0.160 | 1.841 | **0.0001** |
| Residual | 29 | 10.580 | 0.839 | - | - |
| Cropping systems | 1 | 0.400 | 0.032 | 1.017 | 0.3712 |
| Residual | 31 | 12.196 | 0.968 | - | - |
| Study locations | 2 | 1.156 | 0.091 | 1.516 | **0.0001** |
| Residual | 30 | 11.439 | 0.908 | - | - |
| Location, sample, and cropping systems | 9 | 4.431 | 0.352 | 1.387 | **0.0001** |
| Residual | 23 | 8.165 | 0.648 | - | - |

**Supplementary Figures**

**
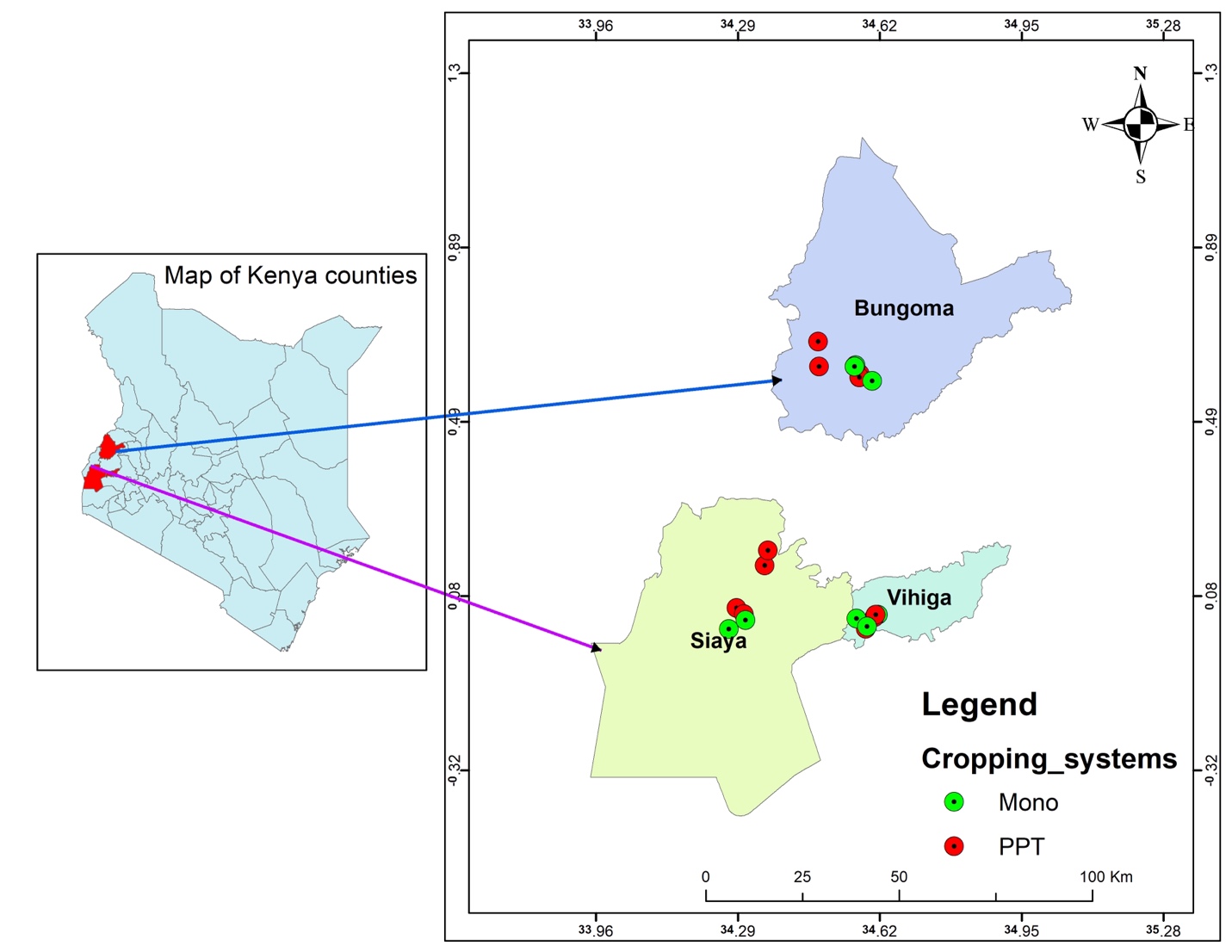
**

**Figure S1:** Map of Kenya showing the smallholder farms where samples were collected at Vihiga, Siaya and Bungoma counties.


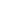

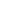

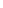

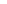

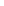

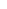

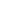

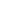

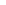


**Figure S2:** Correlograms showing the relationships between the different soil physico-chemical properties; **(A)** Push-pull technology and **(B)** Maize-monoculture cropping system fields. pH, potential of hydrogen; EC, electrical conductivity; P, phosphorus; K, potassium; Na, sodium; Ca, calcium; Mg, magnesium; Fe, iron; Mn, manganese; Cu, copper; Zn, zinc; B, boron; Mo, molybdenum; S, sulphur; N, nitrogen; OC, organic carbon; EA, exchangeable acidity; ESP, exchangeable sodium percentage. Asterisks represent significant effect correlations (***, P < 0.001; **, P < 0.01; and *, P < 0.05).

The differences between the maize-root and soil bacterial and fungal genera were evident in the ASVs across all samples and studied location **(Figs. S3, S4, S5, S6, and S7)**.

**
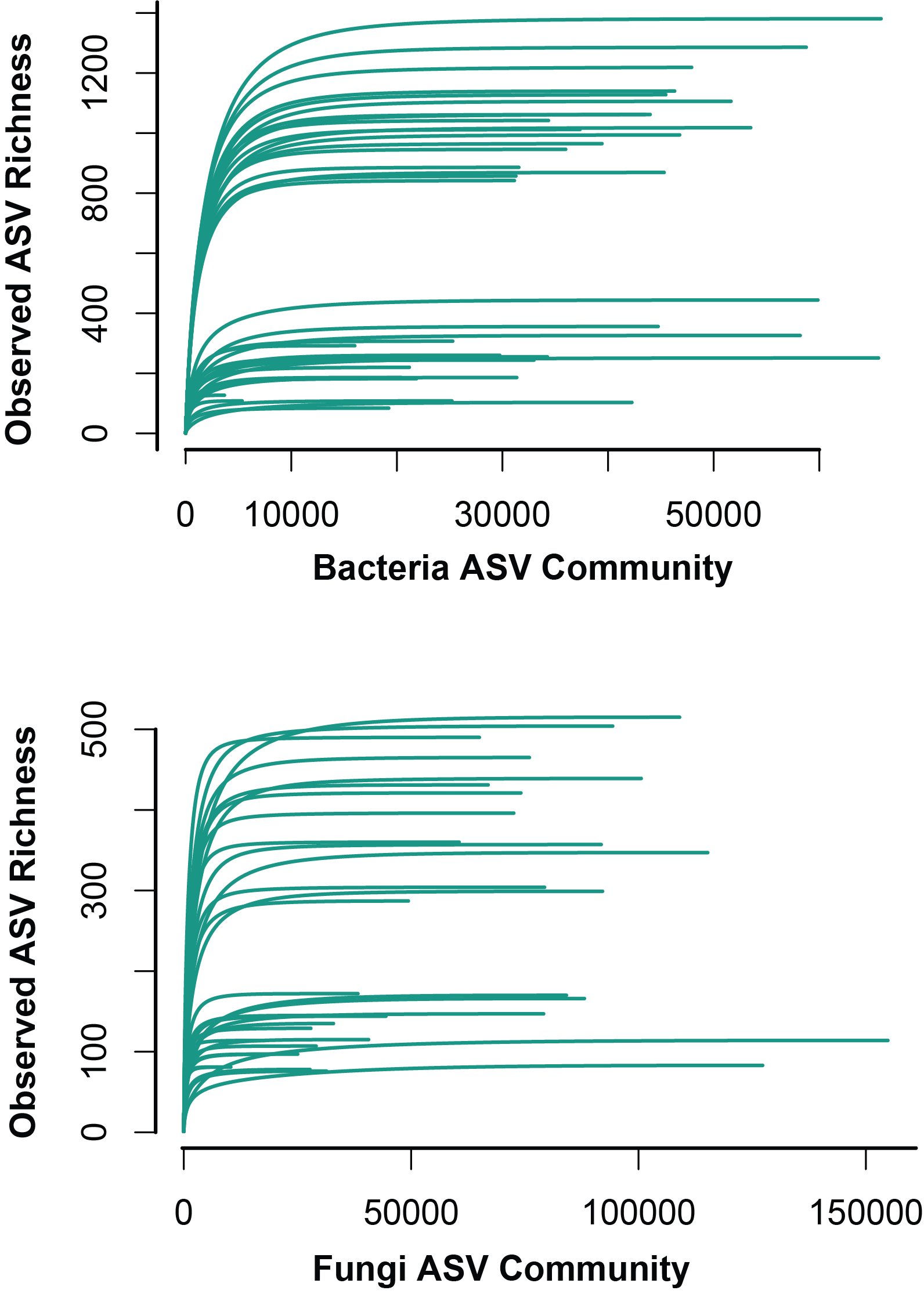
**

**Figure S3:** Rarefaction curves for bacteria and fungi amplicon sequencing variant (ASVs) richness in push-pull and maize-monoculture cropping systems.





**Figure S4:** Soil and maize-root of top 30 bacterial genera communities showing relative abundance in individual farms per sample types; **(A)** Cropping systems of maize-root samples; R, maize-root samples; MR, maize-monoculture root, PR, push-pull maize-root; **(B)** Cropping system of soil samples; S, soil samples; MS, maize-monoculture soil; PS, push-pull soil. Bacterial genera with relative abundances < 1 were grouped as 'Other'.





**Figure S5:** Soil and maize-root of top 30 fungal genera communities showing relative abundance in individual farms per sample type; **(A)** Cropping systems of maize-root samples; R, maize-root samples; MR, maize-monoculture root, PR, push-pull maize-root; **(B)** Cropping systems of soil samples; S, soil samples; MS, maize-monoculture soil; PS, push-pull soil. Fungal genera with relative abundances < 1% were grouped as 'Other'.





**Figure S6:** Soil and maize-root samples of top 30 bacterial genera communities showing relative abundance between study locations. Bacterial genera with relative abundances < 1% were grouped as 'Other'.





**Figure S7:** Soil and maize-root samples of top 30 fungal genera communities showing relative abundance between study locations. Fungal genera with relative abundances < 1% were grouped as 'Other'.
