# Supplementary material for: Long-term push–pull cropping system shifts soil and maize-root microbiome diversity paving way to resilient farming system
Source: BMC Microbiol. 2024 Mar 18;24:92. doi: 10.1186/s12866-024-03238-z (PMC10946131; doi:10.1186/s12866-024-03238-z)
Supplement: Supplementary file 2 — Supplementary Material 2. [file 12866_2024_3238_MOESM2_ESM.zip › Jalloh A.A et al. Push-pull_16S_Additional file_2B_stat_analysis.pdf]

Abundance_Soil_Root_Metagenomics

Ann Laigong

10/18/2022

{r setup, include=FALSE} knitr::opts_chunk$set(echo = TRUE)

Load the packages

```
suppressPackageStartupMessages({
require(csv)
require(dplyr)
require(tidyr)
library(dada2)
library(Cairo)
library(ggtree)
library(VennDiagram)
library(UpSetR)
library("phyloseq"); packageVersion("phyloseq") # Handling and analysis of high-
throughput microbiome census ata. library("vegan");packageVersion("vegan") #
Community Ecology Package. library("ggplot2");packageVersion("ggplot2") #
Create Elegant Data Visualisations Using the Grammar of graphics.
library("dendextend");packageVersion("dendextend")
library("tidyr");packageVersion("tidyr") library("viridis");packageVersion("viridis")
library("reshape");packageVersion("reshape") #install.packages("remotes")
#remotes::install_github("vmikk/metagMisc")
library(metagMisc) library(janitor)
library(coin) # Conditional Inference Procedures in a Permutation Test Framework.
library(reshape2) # Flexibly Reshape Data: A Reboot of the Reshape Package.
#install.packages("ggnewscale")
library(ggnewscale) # Multiple Fill and Colour Scales in 'ggplot2'.
#BiocManager::install("MicrobiotaProcess")
library(MicrobiotaProcess) # an R package for analysis, visualization and biomarker
discovery of Microbiome.
library(patchwork)
library(ape)
#library(ANCOMBC)
library(tidyr)
})
```

```
#(.packages())
```

Loading the data

```
rm(list=ls())
# Import the tax table tax
<-
read.table("C:/Users/icipe/Desktop/active_manuscript/soil/dada2_16s/
ASV_tax_species.tsv", header = TRUE, sep = "\t", row.names = 1) head(tax)
names(tax)
tax<-tax[,-c(9:11)]
#head(tax)
tax<-t(tax)
tax<-as.matrix(tax) #converting tax dataframe to a matrix #head(tax)
tax<-(t(tax))
tax<-phyloseq::tax_table(tax) #converting the tax matrix to phyloseq object
#class(tax)
#head(tax)

#Loading OTU
otu <-
read.table("C:/Users/icipe/Desktop/active_manuscript/soil/dada2_16s/
ASV_table.tsv", header= TRUE, sep = "\t", row.names = 1)

#otu<-otu[,-c(21)]
head(otu)
names(otu)
otu<-otu_table(otu,taxa_are_rows = TRUE) #converting otu matrix to phyloseq
object.
head(otu)

#combine the two physeq object i.e tax,otu physeq
<-phyloseq(otu, tax)
physeq

#importing the metadata
meta<-
read.csv("C:/Users/icipe/Desktop/active_manuscript/soil/dada2_ITS/
soil_metadata.csv",sep = ",", header = TRUE, row.names = 1) head(meta)
#names(meta) = c("Sample_ID","Animal_Type", "Microbiome.Domain","Location")
names(meta)
head(meta)
meta<-sample_data(meta) #converting the metadata to a phyloseq object
```

# we now create a third object called random for mergingwith the other three object
library("ape")
random_tree <- rtree(ntaxa(physeq), rooted = TRUE, tip.label =
taxa_names(physeq))

#merging the preceeding 3 objects.
physeq1 <- merge_phyloseq(physeq, meta, random_tree) physeq1

Data pre-processing

#filtering the unwanted sequences
ps2 <- subset_taxa(physeq1, (Order!="Chloroplast") | is.na(Order)) ntaxa(ps2)
ps2 <- subset_taxa(ps2, (Phylum!="Chloroflexi") | is.na(Phylum)) ntaxa(ps2)
ps2<- subset_taxa(ps2, (Family!="Mitochondria") | is.na(Family)) ntaxa(ps2)
ps2<- subset_taxa(ps2, (Kingdom!="Archaea") | is.na(Kingdom)) ntaxa(ps2)
ps2<- subset_taxa(ps2, (Kingdom!="Eukaryota") | is.na(Kingdom)) ntaxa(ps2)

ps2<-subset_taxa(ps2, !is.na(Phylum) & !Phylum %in% c("", "uncharacterized"))
ntaxa(ps2)

ps2 <- subset_taxa(ps2, (Genus!="Morganella") | is.na(Order)) ntaxa(ps2)

ps2 <- subset_taxa(ps2, (Genus!="Myroides") | is.na(Phylum)) ntaxa(ps2)

ps3 <- prune_taxa(taxa_sums(ps2) > 38, ps2) ps3
#Extracting the filtered taxonomy and feature tables for barplot plotting
tax_table <- phyloseq_to_df(ps3, addtax = T, addtot = F, addmaxrank = F)
cumulation <- tax_table %>% adorn_totals(c("col"))
cumulation <- cumulation[order(cumulation$Total, decreasing = TRUE),]

#merging the blast taxonomic classification to blast abundance table merged_data
<- tax_table
write.csv(merged_data,
file="C:/Users/icipe/Desktop/active_manuscript/soil/dada2_16s/finale_r
esults/abud/Supp_Taxonomic_Classification_Abudance.csv")

```
#grouping the data (entire dataset): Genus, Species and sample names
Featured_table <- merged_data[,c(8,10:47)]
group <- Featured_table %>%
   group_by(Genus)%>%
   summarise_if(is.numeric, sum)
#Groups the data in the defined order which eases downstream analysis group <-
Featured_table %>% group_by(Genus)%>%
   summarise_each(funs(sum),
"R1","R10","R11","R12","R13","R14","R15","R16","R17","R18","R19","R2", "R20",

"R3","R4","R5","R6","R7","R8","R9","S1","S10","S11","S12","S13","S14",
"S15","S16",
                          "S17","S18","S2","S3","S4","S5","S6","S7","S8","S9")
group<-group[-c(1),]
head(group)
```

TREATMENTS

```
#creating multiple dataframes for the different treatments

ps<-group[,c(1,22,24,26,28,30,33,35,37,39)] # Push pull soil ms<-
group[,c(1,23,25,27,29,31,32,34,36,38)] # Monoculture soil pr<-
group[,c(1,2,4,6,8,10,12,15,17,19,21)] # Pushpull root
mr<-group[,c(1,3,5,7,9,11,13,14,16,18,20)] # Monoculture root

all<-group

ps_total <- ps %>% adorn_totals(c("col"))
ps_total <- mutate(ps_total, ps=rowSums(ps_total[11])/10)
ps_total <- ps_total[,c(1,12)]

ms_total <- ms %>% adorn_totals(c("col"))
ms_total <- mutate(ms_total, ms=rowSums(ms_total[11])/10)
ms_total <- ms_total[,c(1,12)]

pr_total <- pr %>% adorn_totals(c("col"))
pr_total <- mutate(pr_total, pr=rowSums(pr_total[12])/11)
pr_total <- pr_total[,c(1,13)]

mr_total <- mr %>% adorn_totals(c("col"))
mr_total <- mutate(mr_total, mr=rowSums(mr_total[12])/11)
mr_total <- mr_total[,c(1,13)]

all_total <- all %>% adorn_totals(c("col"))
all_total <- mutate(all_total, all=rowSums(all_total[40])/39) all_total <-
all_total[,c(1,41)]

# Merging
merged <- Reduce(function(x,y) merge(x,y,by="Genus",all=TRUE),
                          list(ps_total,ms_total,pr_total, mr_total ))
```

```
names(merged)<-c('Genus','Push Soil','Mono Soil','Push Root', 'Mono Root')

#calculating the total abundance per genus and ordering from the most abundant to
the lowest
cumulation <- merged %>% adorn_totals(c("col"))
cumulation <- cumulation[order(cumulation$Total, decreasing = TRUE),]
cumulation$perc = cumulation$Total / sum(cumulation$Total) * 100

tired<-head(cumulation$Genus, n=20) #453 genus #
Using R base append() #install.packages('rlist')
library('rlist')
li2 <- append(tired,"Others") print(li2)
genus_Rep <- li2

group <- aggregate(merged[-1], list(Genus = replace(merged$Genus,!
(merged$Genus %in% genus_Rep), "Others")), sum)
#View(group)



PS<-group[,c(1:2)]
MS<- group[,c(1:3)]
PR<- group[,c(1:4)]
MR<- group[,c(1:5)]
All<- group[,c(1:5)]
```

Viewing Sample Diversity

```
#install.packages("janitor")
library(janitor)
#converting the abudances into percentage
bar_all <- adorn_percentages(All, denominator = "col", na.rm = T) bar_all %>%
   adorn_totals("row") %>%
   adorn_pct_formatting()
dist_all<-bar_all %>%
   adorn_totals("row") %>%
   adorn_pct_formatting()
dist_all write.csv(dist_all,
"C:/Users/icipe/Desktop/active_manuscript/soil/dada2_16s/finale_result
s/abud/Top_20_Abundance_Farming_Type.csv")
#gathering the data
bar_all <- bar_all %>%
   gather(value = "abundance", key = "Farming_type", -Genus) bar_all <-
as.data.frame(gsub("\\(", " (", as.matrix(bar_all)))
```

```
# coerce the dataframe columns into respective data type
bar_all$Genus <- as.factor(bar_all$Genus)
bar_all$Farming_type<-as.character(bar_all$Farming_type)
bar_all$abundance <- as.numeric(bar_all$abundance)

#ordering the data for plotting
bar_all$Genus <- reorder(bar_all$Genus, bar_all$abundance) bar_all$Genus <-
factor(bar_all$Genus, levels=rev(levels(bar_all$Genus)))
bar_all$Genus <- factor(bar_all$Genus,
                                    levels=genus_Rep)

# Defining the color pallete
myPalette <- c("#1B9E77", "#D95F02", "#7570B3", "#E7298A", "#99000D",
"#E6AB02", "#A6761D", "#666666","#FDCDAC", "#1F78B4", "#B2DF8A",
"#33A02C", "#CBD5E8", "#E31A1C", "#FDBF6F",
"#FF7F00","#4A1486","#C0C0C0","#B3E2CD","#FFFF33",  "#5172b2","#F4CAE4",
"#E6F5C9", "#FCFBFD","#139BF1","#09FF00","#065535", "#1D91C0",
"#C0FFEE","#B35806","#0C2C84","#D0ED0E","#092617","#499976","#4D5D53",
"#E48400","#6082B6","#316689","#CEFB02","#738678","#645452","#EEA47FFF
", "#00539CFF", "#FC766AFF", "#42EADDFF", "#00A4CCFF", "#69B3BB",
"#B589D6","#D1DFB7","#97BC62FF","#D198C5FF","#000000", "#CBCE91FF",
"#616247FF", "#D64161FF","#435E55FF", "#DD4132FF","#CE4A7EFF",
"#BD7F37FF","#FFA351FF","#185E57", "#FCE3E3", "#EF6C6C", "#EF6C9A",
"#93103E", "#F7E3FC", "#D56CEF", "#BD19E6", "#8D6CEF", "#DBD1FA",
"#BFC8F8", "#1531BC", "#9FA5F3", "#95C7F3", "#6CEFC8", "#6CEF84",
"#91E619", "#B7CEEC", "#9AFEFF", "#57FEFF", "#78C7C7", "#46C7C7",
"#00A36C", "#728C00", "#4E9268", "#6CC417", "#64E986", "#F5E216",
"#FFCE44", "#8B8000", "#660000", "#610541", "#E56E94", "#F660AB",
"#E3319D", "#FF77FF", "#C45AEC", "#6960EC", "#736AFF", "#F9B7FF",
"#FCDFFF", "#D291BC", "#614051", "#FEA3AA", "#7D0541")

length(myPalette)

# Definig the names in italics
guide_italics <- guides(fill = guide_legend(label.theme = element_text(size = 15,
face = "italic", colour = "Black", angle = 0)))
```

Plotting barplot

```
library(Cairo)
library(forcats)

#plotting the barplot
p_all <- ggplot(bar_all,aes(x = fct_inorder(Farming_type), y = abundance),
labs(fill= Genus), group=row.names(bar_all))+
```

```
xlab("Farming Type")+ ylab("abundance") + geom_col(aes(fill = Genus),position =
position_stack(reverse = FALSE))+
   theme(axis.text.x = element_text(angle = 72,size = 15, hjust = 1, face =
"italic", family = "Arial"))+
     scale_fill_manual(values = myPalette)+ #guides(fill =
   guide_legend(reverse = FALSE))+ guide_italics+
   theme(legend.text = element_text(size = 8, colour = "black", face = "italic",
family = "Arial"), legend.text.align = 0)+
   theme(axis.text.y = element_text(angle = 0, vjust = 0.5, size = 8, family =
"Arial"))+
   theme(axis.text = element_text(colour = "black", size = 8, family = "Arial"))+
   theme(axis.line = element_line())+
   theme(panel.background = element_rect(fill = "white"),plot.margin =
margin(0.1, 0.1, 0.1, 0.1, "cm"), plot.background = element_rect(colour = NULL,
size = 1))+
   theme(axis.ticks.length.y = unit(.15, "cm"), axis.ticks.length.x = unit(.25, "cm"),
axis.text.x = element_text(margin = margin(t = .3, unit = "cm")))+
   theme(legend.position = "right", legend.justification = "top", legend.direction =
"vertical", legend.text = element_text(size = 10))+
   theme(legend.key = element_rect(fill = "white"))+ theme(legend.title =
   element_text(face = NULL, size = 8, family =
"Arial"))+
   theme(panel.background = element_blank(), axis.text = element_blank())+
   theme(axis.text = element_text(colour = "black", size = 8, family = "Arial"))

#install.packages('extrafont')
library(extrafont) #font_import
#BiocManager::install("ggpubr")

p_fmty_20<-p_all
p_fmty_20

ggsave("C:/Users/icipe/Desktop/active_manuscript/soil/dada2_16s/
finale_results/abud/Top_20_Genus_Abundance_Farming_Type.jpeg",
          width = 18, height = 12, dpi = 600)

ggsave("C:/Users/icipe/Desktop/active_manuscript/soil/dada2_16s/finale
_results/abud/Top_20_Genus_Abundance_Farming_Type.png",
          width = 18, height = 12, dpi = 600)

ggsave("C:/Users/icipe/Desktop/active_manuscript/soil/dada2_16s/finale
_results/abud/Top_20_Genus_Abundance_Farming_Type.svg",
          width = 18, height = 12, dpi = 600)
```

```
ggsave("C:/Users/icipe/Desktop/active_manuscript/soil/dada2_16s/finale
_results/abud/Top_20_Genus_Abundance_Farming_Type.tiff", width
          = 18, height = 12, dpi = 600)
```

SOIL_ROOT COMBINED VISUALIZATION GENUS

```
#grouping the data (entire dataset): Genus, Species and sample names
Featured_table <- merged_data[,c(8,10:47)]
group_sr <- Featured_table %>%
   group_by(Genus)%>%
   summarise_if(is.numeric, sum)
#Groups the data in the defined order which eases downstream analysis group_sr
<- Featured_table %>% group_by(Genus)%>%
   summarise_each(funs(sum),
"R1","R10","R11","R12","R13","R14","R15","R16","R17","R18","R19","R2", "R20",

"R3","R4","R5","R6","R7","R8","R9","S1","S10","S11","S12","S13","S14",
"S15","S16",
                         "S17","S18","S2","S3","S4","S5","S6","S7","S8","S9")
group_sr<-group_sr[-c(1),]
head (group_sr)
#creating multiple dataframes for the different treatments

soil<-group_sr[,c(1,22:39)]
root<-group_sr[,c(1:21)]
all_sr<-group_sr


dim(soil)
soil_total <- soil %>% adorn_totals(c("col"))
soil_total <- mutate(soil_total, soil=rowSums(soil_total[20])/19) soil_total <-
soil_total[,c(1,21)]

dim(root)
root_total <- root %>% adorn_totals(c("col"))
root_total <- mutate(root_total, root=rowSums(root_total[22])/21) root_total <-
root_total[,c(1,23)]

dim(all_sr)
all_sr_total <- all_sr %>% adorn_totals(c("col")) all_sr_total <-
mutate(all_sr_total, all_sr=rowSums(all_sr_total[40])/39)
all_sr_total <- all_sr_total[,c(1,41)]

# Merging
merged_sr <- Reduce(function(x,y) merge(x,y,by="Genus",all=TRUE),
                         list(root_total,soil_total ))

names(merged_sr)<-c('Genus','Root','Soil')
```

```
#calculating the total abundance per genus and ordering from the most abundant to the lowest
cumulation_sr <- merged_sr %>% adorn_totals(c("col"))
cumulation_sr <- cumulation_sr[order(cumulation_sr$Total, decreasing = TRUE),]
cumulation_sr$perc = cumulation_sr$Total / sum(cumulation_sr$Total) * 100

tired_sr<-head(cumulation_sr$Genus, n=20) #
Using R base append() #install.packages('rlist')
library('rlist')
li2 <- append(tired_sr,"Others")
print(li2)
genus_Rep <- li2

group_sr <- aggregate(merged_sr[-1], list(Genus =
replace(merged_sr$Genus,!(merged_sr$Genus %in% genus_Rep), "Others")), sum)
#View(group)

head(group_sr)
ROOT<-group_sr[,c(1:2)]
SOIL<-group_sr[,c(1,3)]
All_sr<- group_sr[,c(1:3)]
```

Viewing Sample Diversity

```
#install.packages("janitor")
library(janitor)
#converting the abudances into percentage
bar_all_sr <- adorn_percentages(All_sr, denominator = "col", na.rm = T)
bar_all_sr %>% adorn_totals("row")
   %>% adorn_pct_formatting()
dist_all_sr<-bar_all_sr %>%
   adorn_totals("row") %>%
   adorn_pct_formatting()
dist_all_sr write.csv(dist_all_sr,
"C:/Users/icipe/Desktop/active_manuscript/soil/dada2_16s/finale_result
s/abud/Top_20_Abundance_Sample_Type_Soil_Root.csv")
#gathering the data bar_all_sr <-
bar_all_sr %>%
   gather(value = "abundance", key = "Sample_type", -Genus)
bar_all_sr <- as.data.frame(gsub("\\(", " (", as.matrix(bar_all_sr)))
```

```
# coerce the dataframe columns into respective data type
bar_all_sr$Genus <- as.factor(bar_all_sr$Genus)
bar_all_sr$Sample_type<- as.character(bar_all_sr$Sample_type)
bar_all_sr$abundance <- as.numeric(bar_all_sr$abundance)

#ordering the data for plotting
bar_all_sr$Genus <- reorder(bar_all_sr$Genus, bar_all_sr$abundance)
bar_all_sr$Genus <- factor(bar_all_sr$Genus, levels=rev(levels(bar_all_sr$Genus)))
bar_all_sr$Genus <- factor(bar_all_sr$Genus,
                           levels=genus_Rep)

# Defining the color pallete
myPalette <- c("#1B9E77", "#D95F02", "#7570B3", "#E7298A", "#99000D",
"#E6AB02", "#A6761D", "#666666","#FDCDAC", "#1F78B4", "#B2DF8A",
"#33A02C", "#CBD5E8", "#E31A1C", "#FDBF6F",
"#FF7F00","#4A1486","#C0C0C0","#B3E2CD","#FFFF33",  "#5172b2","#F4CAE4",
"#E6F5C9", "#FCFBFD","#139BF1","#09FF00","#065535", "#1D91C0",
"#C0FFEE","#B35806","#0C2C84","#D0ED0E","#092617","#499976","#4D5D53",
"#E48400","#6082B6","#316689","#CEFB02","#738678","#645452","#EEA47FFF
", "#00539CFF", "#FC766AFF", "#42EADDFF", "#00A4CCFF", "#69B3BB",
"#B589D6","#D1DFB7","#97BC62FF","#D198C5FF","#000000", "#CBCE91FF",
"#616247FF", "#D64161FF","#435E55FF", "#DD4132FF","#CE4A7EFF",
"#BD7F37FF","#FFA351FF","#185E57", "#FCE3E3", "#EF6C6C", "#EF6C9A",
"#93103E", "#F7E3FC", "#D56CEF", "#BD19E6", "#8D6CEF", "#DBD1FA",
"#BFC8F8", "#1531BC", "#9FA5F3", "#95C7F3", "#6CEFC8", "#6CEF84",
"#91E619", "#B7CEEC", "#9AFEFF", "#57FEFF", "#78C7C7", "#46C7C7",
"#00A36C", "#728C00", "#4E9268", "#6CC417", "#64E986", "#F5E216",
"#FFCE44", "#8B8000", "#660000", "#610541", "#E56E94", "#F660AB",
"#E3319D", "#FF77FF", "#C45AEC", "#6960EC", "#736AFF", "#F9B7FF",
"#FCDFFF", "#D291BC", "#614051", "#FEA3AA", "#7D0541")

length(myPalette)

# Definig the names in italics
guide_italics <- guides(fill = guide_legend(label.theme = element_text(size = 15,
face = "italic", colour = "Black", angle = 0)))
```

Plotting barplot

```
library(Cairo)
library(forcats)

#plotting the barplot
psr_all <- ggplot(bar_all_sr,aes(x = fct_inorder(Sample_type), y = abundance),
labs(fill= Genus), group=row.names(bar_all_sr))+ xlab("Sample Type")+
ylab("abundance") + geom_col(aes(fill =
```

```
Genus),position = position_stack(reverse = FALSE))+ theme(axis.text.x =
   element_text(angle = 72,size = 15, hjust = 1,
face = "italic", family = "Arial"))+
     scale_fill_manual(values = myPalette)+
   #guides(fill = guide_legend(reverse = FALSE))+ guide_italics+
   theme(legend.text = element_text(size = 8, colour = "black", face = "italic",
family = "Arial"), legend.text.align = 0)+
   theme(axis.text.y = element_text(angle = 0, vjust = 0.5, size = 8, family =
"Arial"))+
   theme(axis.text = element_text(colour = "black", size = 8, family = "Arial"))+
   theme(axis.line = element_line())+
   theme(panel.background = element_rect(fill = "white"),plot.margin =
margin(0.1, 0.1, 0.1, 0.1, "cm"), plot.background = element_rect(colour = NULL,
size = 1))+
   theme(axis.ticks.length.y = unit(.15, "cm"), axis.ticks.length.x = unit(.25, "cm"),
axis.text.x = element_text(margin = margin(t = .3, unit = "cm")))+
   theme(legend.position = "right", legend.justification = "top", legend.direction =
"vertical", legend.text = element_text(size = 10))+
   theme(legend.key = element_rect(fill = "white"))+ theme(legend.title =
   element_text(face = NULL, size = 8, family =
"Arial"))+
   theme(panel.background = element_blank(), axis.text = element_blank())+
   theme(axis.text = element_text(colour = "black", size = 8, family = "Arial"))

#install.packages('extrafont')
library(extrafont) #font_import
#BiocManager::install("ggpubr")

psr_genus_20<-psr_all psr_genus_20

ggsave("C:/Users/icipe/Desktop/active_manuscript/soil/dada2_16s/
finale_results/abud/Top_20_Genus_Abundance_Sampling_Type.SR.jpeg",
          width = 20, height = 12, dpi = 600)

ggsave("C:/Users/icipe/Desktop/active_manuscript/soil/dada2_16s/finale
_results/abud/Top_20_Genus_Abundance_Sampling_Type.SR.png", width
          = 20, height = 12, dpi = 600)

ggsave("C:/Users/icipe/Desktop/active_manuscript/soil/dada2_16s/finale
_results/abud/Top_20_Genus_Abundance_Sampling_Type.SR.svg", width
          = 20, height = 12, dpi = 600)

ggsave("C:/Users/icipe/Desktop/active_manuscript/soil/dada2_16s/finale
```

```
_results/abud/Top_20_Genus_Abundance_Sampling_Type.SR.tiff", width
          = 20, height = 12, dpi = 600)
```

LOCATION VISUALIZATION GENUS

```
#grouping the data (entire dataset): Genus, Species and sample names
Featured_table <- merged_data[,c(8,10:47)]
group_lc <- Featured_table %>%
   group_by(Genus)%>%
   summarise_if(is.numeric, sum)
#Groups the data in the defined order which eases downstream analysis group_lc
<- Featured_table %>% group_by(Genus)%>%
   summarise_each(funs(sum),
"R1","R10","R11","R12","R13","R14","R15","R16","R17","R18","R19","R2", "R20",

"R3","R4","R5","R6","R7","R8","R9","S1","S10","S11","S12","S13","S14",
"S15","S16",
                          "S17","S18","S2","S3","S4","S5","S6","S7","S8","S9")
group_lc<-group_lc[-c(1),]
head (group_lc)
#creating multiple dataframes for the different treatments

bungoma<-group_lc[,c(1,10:12,14,30:31)]
siaya<-group_lc[,c(1,2:3,13,15:23,32:39)]
vihiga<-group_lc[,c(1,4:9,24:29)]

dim(bungoma)
bungoma_total <- bungoma %>% adorn_totals(c("col"))
bungoma_total <- mutate(bungoma_total,
bungoma=rowSums(bungoma_total[8])/7)
bungoma_total <- bungoma_total[,c(1,9)]

dim(siaya)
siaya_total <- siaya %>% adorn_totals(c("col"))
siaya_total <- mutate(siaya_total, siaya=rowSums(siaya_total[22])/21) siaya_total
<- siaya_total[,c(1,23)]

dim(vihiga)
vihiga_total <- vihiga %>% adorn_totals(c("col")) vihiga_total <-
mutate(vihiga_total, vihiga=rowSums(vihiga_total[14])/13)
vihiga_total <- vihiga_total[,c(1,15)]

# Merging
merged_lc <- Reduce(function(x,y) merge(x,y,by="Genus",all=TRUE),
                          list(bungoma_total,siaya_total, vihiga_total))

names(merged_lc)<-c('Genus','Bungoma','Siaya','Vihiga')
```

```
#calculating the total abundance per genus and ordering from the most abundant to
the lowest
cumulation_lc <- merged_lc %>% adorn_totals(c("col"))
cumulation_lc <- cumulation_lc[order(cumulation_lc$Total, decreasing = TRUE),]
cumulation_lc$perc = cumulation_lc$Total / sum(cumulation_lc$Total) * 100

tired_lc<-head(cumulation_lc$Genus, n=20) #
Using R base append() #install.packages('rlist')
library('rlist')
li2 <- append(tired_lc,"Others")
print(li2)
genus_Rep <- li2

group_lc <- aggregate(merged_lc[-1], list(Genus =
replace(merged_lc$Genus,!(merged_lc$Genus %in% genus_Rep), "Others")), sum)
#View(group)

head(group_lc)

BUNGOMA<-group_lc[,c(1:2)]
SIAYA<-group_lc[,c(1,3)]
VIHIGA<-group_lc[,c(1,4)]
All_lc<- group_lc[,c(1:4)]
```

Viewing Sample Diversity

```
#install.packages("janitor")
library(janitor)
#converting the abudances into percentage
bar_all_lc <- adorn_percentages(All_lc, denominator = "col", na.rm = T)
bar_all_lc %>% adorn_totals("row")
   %>% adorn_pct_formatting()
dist_all_lc<-bar_all_lc %>%
   adorn_totals("row") %>%
   adorn_pct_formatting()
dist_all_lc write.csv(dist_all_lc,
"C:/Users/icipe/Desktop/active_manuscript/soil/dada2_16s/finale_result
s/abud/Top_20_Abundance_Location.csv")
#gathering the data bar_all_lc <-
bar_all_lc %>%
   gather(value = "abundance", key = "Location", -Genus)
bar_all_lc <- as.data.frame(gsub("\\(", " (", as.matrix(bar_all_lc)))
```

```
# coerce the dataframe columns into respective data type
bar_all_lc$Genus <- as.factor(bar_all_lc$Genus)
bar_all_lc$Sample_type<- as.character(bar_all_lc$Location)
bar_all_lc$abundance <- as.numeric(bar_all_lc$abundance)

#ordering the data for plotting
bar_all_lc$Genus <- reorder(bar_all_lc$Genus, bar_all_lc$abundance)
bar_all_lc$Genus <- factor(bar_all_lc$Genus, levels=rev(levels(bar_all_lc$Genus)))
bar_all_lc$Genus <- factor(bar_all_lc$Genus,
                           levels=genus_Rep)

# Defining the color pallete
myPalette <- c("#1B9E77", "#D95F02", "#7570B3", "#E7298A", "#99000D",
"#E6AB02", "#A6761D", "#666666","#FDCDAC", "#1F78B4", "#B2DF8A",
"#33A02C", "#CBD5E8", "#E31A1C", "#FDBF6F",
"#FF7F00","#4A1486","#C0C0C0","#B3E2CD","#FFFF33",  "#5172b2","#F4CAE4",
"#E6F5C9", "#FCFBFD","#139BF1","#09FF00","#065535", "#1D91C0",
"#C0FFEE","#B35806","#0C2C84","#D0ED0E","#092617","#499976","#4D5D53",
"#E48400","#6082B6","#316689","#CEFB02","#738678","#645452","#EEA47FFF
", "#00539CFF", "#FC766AFF", "#42EADDFF", "#00A4CCFF", "#69B3BB",
"#B589D6","#D1DFB7","#97BC62FF","#D198C5FF","#000000", "#CBCE91FF",
"#616247FF", "#D64161FF","#435E55FF", "#DD4132FF","#CE4A7EFF",
"#BD7F37FF","#FFA351FF","#185E57", "#FCE3E3", "#EF6C6C", "#EF6C9A",
"#93103E", "#F7E3FC", "#D56CEF", "#BD19E6", "#8D6CEF", "#DBD1FA",
"#BFC8F8", "#1531BC", "#9FA5F3", "#95C7F3", "#6CEFC8", "#6CEF84",
"#91E619", "#B7CEEC", "#9AFEFF", "#57FEFF", "#78C7C7", "#46C7C7",
"#00A36C", "#728C00", "#4E9268", "#6CC417", "#64E986", "#F5E216",
"#FFCE44", "#8B8000", "#660000", "#610541", "#E56E94", "#F660AB",
"#E3319D", "#FF77FF", "#C45AEC", "#6960EC", "#736AFF", "#F9B7FF",
"#FCDFFF", "#D291BC", "#614051", "#FEA3AA", "#7D0541")

length(myPalette)

# Definig the names in italics
guide_italics <- guides(fill = guide_legend(label.theme = element_text(size = 15,
face = "italic", colour = "Black", angle = 0)))
```

Plotting barplot

```
library(Cairo)
library(forcats)

#plotting the barplot
plc_all <- ggplot(bar_all_lc,aes(x = fct_inorder(Location), y = abundance),
labs(fill= Genus), group=row.names(bar_all_lc))+
```

```
xlab("Location")+ ylab("abundance") + geom_col(aes(fill = Genus),position =
position_stack(reverse = FALSE))+
   theme(axis.text.x = element_text(angle = 72,size = 15, hjust = 1, face =
"italic", family = "Arial"))+
     scale_fill_manual(values = myPalette)+ #guides(fill =
   guide_legend(reverse = FALSE))+ guide_italics+
   theme(legend.text = element_text(size = 8, colour = "black", face = "italic",
family = "Arial"), legend.text.align = 0)+
   theme(axis.text.y = element_text(angle = 0, vjust = 0.5, size = 8, family =
"Arial"))+
   theme(axis.text = element_text(colour = "black", size = 8, family = "Arial"))+
   theme(axis.line = element_line())+
   theme(panel.background = element_rect(fill = "white"),plot.margin =
margin(0.1, 0.1, 0.1, 0.1, "cm"), plot.background = element_rect(colour = NULL,
size = 1))+
   theme(axis.ticks.length.y = unit(.15, "cm"), axis.ticks.length.x = unit(.25, "cm"),
axis.text.x = element_text(margin = margin(t = .3, unit = "cm")))+
   theme(legend.position = "right", legend.justification = "top", legend.direction =
"vertical", legend.text = element_text(size = 10))+
   theme(legend.key = element_rect(fill = "white"))+ theme(legend.title =
   element_text(face = NULL, size = 8, family =
"Arial"))+
   theme(panel.background = element_blank(), axis.text = element_blank())+
   theme(axis.text = element_text(colour = "black", size = 8, family = "Arial"))

#install.packages('extrafont')
library(extrafont) #font_import
#BiocManager::install("ggpubr")

plc_genus_20<-plc_all plc_genus_20

ggsave("C:/Users/icipe/Desktop/active_manuscript/soil/dada2_16s/
finale_results/abud/Top_20_Genus_Abundance_Location.jpeg",
          width = 20, height = 12, dpi = 600)

ggsave("C:/Users/icipe/Desktop/active_manuscript/soil/dada2_16s/finale
_results/abud/Top_20_Genus_Abundance_Location.png",
          width = 20, height = 12, dpi = 600)

ggsave("C:/Users/icipe/Desktop/active_manuscript/soil/dada2_16s/finale
_results/abud/Top_20_Genus_Abundance_Location.svg",
          width = 20, height = 12, dpi = 600)
```

```
ggsave("C:/Users/icipe/Desktop/active_manuscript/soil/dada2_16s/finale
_results/abud/Top_20_Genus_Abundance_Location.tiff", width
        = 20, height = 12, dpi = 600)
```

COMBINING: Sample Type, Farming Type, and Location

```
#psr_genus_20
#p_fmty_20
#plc_genus_20


psr_genus_20<-psr_genus_20 + stat_ellipse()  + labs(tag = "A")
p_fmty_20<- p_fmty_20 + stat_ellipse()  + labs(tag = "B")
plc_genus_20<- plc_genus_20+ stat_ellipse()  + labs(tag = "C")
require(ggplot2)
require(dplyr)
require(svglite)
library(ggpubr)

ggarrange(psr_genus_20,p_fmty_20,plc_genus_20,
            ncol = 2, nrow = 2) + geom_point()



ggsave("C:/Users/icipe/Desktop/active_manuscript/soil/dada2_16s/
finale_results/pub/Top_20_Bacteria_Genus_combined.jpeg",
        width = 36, height = 24, dpi = 600)

ggsave("C:/Users/icipe/Desktop/active_manuscript/soil/dada2_16s/finale
_results/pub/Top_20_Bacteria_Genus_combined.png", width
        = 36, height = 24, dpi = 600)

ggsave("C:/Users/icipe/Desktop/active_manuscript/soil/dada2_16s/finale
_results/pub/Top_20_Bacteria_Genus_combined.svg", width =
        36, height = 24, dpi = 600)

ggsave("C:/Users/icipe/Desktop/active_manuscript/soil/dada2_16s/finale
_results/pub/Top_20_Bacteria_Genus_combined.tiff", width =
        36, height = 24, dpi = 600)
```

ROOTS SAMPLES DIVERSITY

```
#creating multiple dataframes for the different treatments
#Groups the data in the defined order which eases downstream analysis group <-
Featured_table %>% group_by(Genus)%>%
  summarise_each(funs(sum),
"R1","R10","R11","R12","R13","R14","R15","R16","R17","R18","R19","R2", "R20",
```

```
"R3","R4","R5","R6","R7","R8","R9","S1","S10","S11","S12","S13","S14",
"S15","S16",
                        "S17","S18","S2","S3","S4","S5","S6","S7","S8","S9")
group<-group[-c(1),]
head(group)


R1_PR<-group[,c(1:2)]
R3_PR<-group[,c(1,15)]
R5_PR<-group[,c(1,17)]
R7_PR<-group[,c(1,19)]
R9_PR<-group[,c(1,21)]
R11_PR<-group[,c(1,4)]
R13_PR<-group[,c(1,6)]
R15_PR<-group[,c(1,8)]
R17_PR<-group[,c(1,10)]
R19_PR<-group[,c(1,12)]
R2_MR<-group[,c(1,13)]
R4_MR<-group[,c(1,16)]
R6_MR<-group[,c(1,18)]
R8_MR<-group[,c(1,20)]
R10_MR<-group[,c(1,3)]
R12_MR<-group[,c(1,5)]
R14_MR<-group[,c(1,7)]
R16_MR<-group[,c(1,9)]
R18_MR<-group[,c(1,11)]
R20_MR<-group[,c(1,14)]

dim(R1_PR)
R1_PR_total <- R1_PR%>% adorn_totals(c("col"))
R1_PR_total <- mutate(R1_PR_total, R1_PR=rowSums(R1_PR_total[3])/2)
R1_PR_total <- R1_PR_total[,c(1,4)]

R3_PR_total <- R3_PR%>% adorn_totals(c("col"))
R3_PR_total <- mutate(R3_PR_total, R3_PR=rowSums(R3_PR_total[3])/2)
R3_PR_total <- R3_PR_total[,c(1,4)]

R5_PR_total <- R5_PR%>% adorn_totals(c("col"))
R5_PR_total <- mutate(R5_PR_total, R5_PR=rowSums(R5_PR_total[3])/2)
R5_PR_total <- R5_PR_total[,c(1,4)]

R7_PR_total <- R7_PR%>% adorn_totals(c("col"))
R7_PR_total <- mutate(R7_PR_total, R7_PR=rowSums(R7_PR_total[3])/2)
R7_PR_total <- R7_PR_total[,c(1,4)]

R9_PR_total <- R9_PR%>% adorn_totals(c("col"))
R9_PR_total <- mutate(R9_PR_total, R9_PR=rowSums(R9_PR_total[3])/2)
R9_PR_total <- R9_PR_total[,c(1,4)]

R11_PR_total <- R11_PR%>% adorn_totals(c("col"))
```

```
R11_PR_total <- mutate(R11_PR_total,
R11_PR=rowSums(R11_PR_total[3])/2)
R11_PR_total <- R11_PR_total[,c(1,4)]

R13_PR_total <- R13_PR%>% adorn_totals(c("col")) R13_PR_total
<- mutate(R13_PR_total, R13_PR=rowSums(R13_PR_total[3])/2)
R13_PR_total <- R13_PR_total[,c(1,4)]

R15_PR_total <- R15_PR%>% adorn_totals(c("col")) R15_PR_total
<- mutate(R15_PR_total, R15_PR=rowSums(R15_PR_total[3])/2)
R15_PR_total <- R15_PR_total[,c(1,4)]

R17_PR_total <- R17_PR%>% adorn_totals(c("col")) R17_PR_total
<- mutate(R17_PR_total, R17_PR=rowSums(R17_PR_total[3])/2)
R17_PR_total <- R17_PR_total[,c(1,4)]

R19_PR_total <- R19_PR%>% adorn_totals(c("col")) R19_PR_total
<- mutate(R19_PR_total, R19_PR=rowSums(R19_PR_total[3])/2)
R19_PR_total <- R19_PR_total[,c(1,4)]

R2_MR_total <- R2_MR%>% adorn_totals(c("col"))
R2_MR_total <- mutate(R2_MR_total, R2_MR=rowSums(R2_MR_total[3])/2)
R2_MR_total <- R2_MR_total[,c(1,4)]

R4_MR_total <- R4_MR%>% adorn_totals(c("col"))
R4_MR_total <- mutate(R4_MR_total, R4_MR=rowSums(R4_MR_total[3])/2)
R4_MR_total <- R4_MR_total[,c(1,4)]

R6_MR_total <- R6_MR%>% adorn_totals(c("col"))
R6_MR_total <- mutate(R6_MR_total, R6_MR=rowSums(R6_MR_total[3])/2)
R6_MR_total <- R6_MR_total[,c(1,4)]

R8_MR_total <- R8_MR%>% adorn_totals(c("col"))
R8_MR_total <- mutate(R8_MR_total, R8_MR=rowSums(R8_MR_total[3])/2)
R8_MR_total <- R8_MR_total[,c(1,4)]

R10_MR_total <- R10_MR%>% adorn_totals(c("col"))
R10_MR_total <- mutate(R10_MR_total,
R10_MR=rowSums(R10_MR_total[3])/2)
R10_MR_total <- R10_MR_total[,c(1,4)]

R12_MR_total <- R12_MR%>% adorn_totals(c("col"))
R12_MR_total <- mutate(R12_MR_total,
R12_MR=rowSums(R12_MR_total[3])/2)
R12_MR_total <- R12_MR_total[,c(1,4)]
```

```
R14_MR_total <- R14_MR%>% adorn_totals(c("col"))
R14_MR_total <- mutate(R14_MR_total,
R14_MR=rowSums(R14_MR_total[3])/2)
R14_MR_total <- R14_MR_total[,c(1,4)]

R16_MR_total <-R16_MR%>% adorn_totals(c("col"))
R16_MR_total <- mutate(R16_MR_total,
R16_MR=rowSums(R16_MR_total[3])/2)
R16_MR_total <- R16_MR_total[,c(1,4)]

R18_MR_total <- R18_MR%>% adorn_totals(c("col"))
R18_MR_total <- mutate(R18_MR_total,
R18_MR=rowSums(R18_MR_total[3])/2)
R18_MR_total <- R18_MR_total[,c(1,4)]

R20_MR_total <- R20_MR%>% adorn_totals(c("col"))
R20_MR_total <- mutate(R20_MR_total,
R20_MR=rowSums(R20_MR_total[3])/2)
R20_MR_total <- R20_MR_total[,c(1,4)]


# Merging
merged <- Reduce(function(x,y) merge(x,y,by="Genus",all=TRUE),
                      list(R1_PR_total, R3_PR_total,
R5_PR_total,R7_PR_total,R9_PR_total,

R11_PR_total,R13_PR_total,R15_PR_total,R17_PR_total,R19_PR_total,

R2_MR_total,R4_MR_total,R6_MR_total,R8_MR_total,R10_MR_total,

R12_MR_total,R14_MR_total,R16_MR_total,R18_MR_total,R20_MR_total))

names(merged)<-c('Genus','R1_PR', 'R3_PR',
'R5_PR','R7_PR','R9_PR','R11_PR','R13_PR',

'R15_PR','R17_PR','R19_PR','R2_MR','R4_MR','R6_MR','R8_MR','R10_MR',
                      'R12_MR','R14_MR','R16_MR','R18_MR','R20_MR')

#calculating the total abundance per genus and ordering from the most abundant to
the lowest
cumulation <- merged %>% adorn_totals(c("col"))
cumulation <- cumulation[order(cumulation$Total, decreasing = TRUE),]
cumulation$perc = cumulation$Total / sum(cumulation$Total) * 100

tired<-head(cumulation$Genus, n=20) #453 genus #
Using R base append() #install.packages('rlist')
library('rlist')
```

```
li2 <- append(tired,"Others") print(li2)
genus_Rep <- li2

group <- aggregate(merged[-1], list(Genus = replace(merged$Genus,!
(merged$Genus %in% genus_Rep), "Others")), sum)
#View(group)

dim(group)
All<- group[,c(1:21)]
```

Viewing Sample Diversity

```
#install.packages("janitor")
library(janitor)
#converting the abudances into percentage
bar_all <- adorn_percentages(All, denominator = "col", na.rm = T) bar_all %>%
   adorn_totals("row") %>%
   adorn_pct_formatting()
dist_all<-bar_all %>%
   adorn_totals("row") %>%
   adorn_pct_formatting()
dist_all write.csv(dist_all,
"C:/Users/icipe/Desktop/active_manuscript/soil/dada2_16s/finale_result
s/abud/Top_20_Abundance_Root_Samples.csv")
#gathering the data
bar_all <- bar_all %>%
   gather(value = "abundance", key = "Farming_type", -Genus) bar_all <-
as.data.frame(gsub("\\(", " (", as.matrix(bar_all)))

# coerce the dataframe columns into respective data type
bar_all$Genus <- as.factor(bar_all$Genus)
bar_all$Farming_type<- as.character(bar_all$Farming_type)
bar_all$abundance <- as.numeric(bar_all$abundance)

#ordering the data for plotting
bar_all$Genus <- reorder(bar_all$Genus, bar_all$abundance) bar_all$Genus <-
factor(bar_all$Genus, levels=rev(levels(bar_all$Genus)))
bar_all$Genus <- factor(bar_all$Genus,
                                    levels=genus_Rep)

# Defining the color pallete
myPalette <- c("#1B9E77", "#D95F02", "#7570B3", "#E7298A", "#99000D",
"#E6AB02", "#A6761D", "#666666","#FDCDAC", "#1F78B4", "#B2DF8A",
"#33A02C", "#CBD5E8", "#E31A1C", "#FDBF6F",
"#FF7F00","#4A1486","#C0C0C0","#B3E2CD","#FFFF33",
```

```
"#5172b2","#F4CAE4", "#E6F5C9",
"#FCFBFD","#139BF1","#09FF00","#065535", "#1D91C0",
"#C0FFEE","#B35806","#0C2C84","#D0ED0E","#092617","#499976","#4D5D53",
"#E48400","#6082B6","#316689","#CEFB02","#738678","#645452","#EEA47FFF
", "#00539CFF", "#FC766AFF", "#42EADDFF", "#00A4CCFF", "#69B3BB",
"#B589D6","#D1DFB7","#97BC62FF","#D198C5FF","#000000", "#CBCE91FF",
"#616247FF", "#D64161FF","#435E55FF", "#DD4132FF","#CE4A7EFF",
"#BD7F37FF","#FFA351FF","#185E57", "#FCE3E3", "#EF6C6C", "#EF6C9A",
"#93103E", "#F7E3FC", "#D56CEF", "#BD19E6", "#8D6CEF", "#DBD1FA",
"#BFC8F8", "#1531BC", "#9FA5F3", "#95C7F3", "#6CEFC8", "#6CEF84",
"#91E619", "#B7CEEC", "#9AFEFF", "#57FEFF", "#78C7C7", "#46C7C7",
"#00A36C", "#728C00", "#4E9268", "#6CC417", "#64E986", "#F5E216",
"#FFCE44", "#8B8000", "#660000", "#610541", "#E56E94", "#F660AB",
"#E3319D", "#FF77FF", "#C45AEC", "#6960EC", "#736AFF", "#F9B7FF",
"#FCDFFF", "#D291BC", "#614051", "#FEA3AA", "#7D0541")

length(myPalette)

# Definig the names in italics
guide_italics <- guides(fill = guide_legend(label.theme = element_text(size = 15,
face = "italic", colour = "Black", angle = 0)))
```

Plotting barplot

```
library(Cairo)
library(forcats)

#plotting the barplot
p_all <- ggplot(bar_all,aes(x = fct_inorder(Farming_type), y = abundance),
labs(fill= Genus), group=row.names(bar_all))+ xlab("Root Sample")+
ylab("abundance") + geom_col(aes(fill = Genus),position = position_stack(reverse
= FALSE))+
   theme(axis.text.x = element_text(angle = 72,size = 15, hjust = 1, face =
"italic", family = "Arial"))+
     scale_fill_manual(values = myPalette)+ #guides(fill =
   guide_legend(reverse = FALSE))+ guide_italics+
   theme(legend.text = element_text(size = 8, colour = "black", face = "italic",
family = "Arial"), legend.text.align = 0)+
   theme(axis.text.y = element_text(angle = 0, vjust = 0.5, size = 8, family =
"Arial"))+
   theme(axis.text = element_text(colour = "black", size = 8, family = "Arial"))+
   theme(axis.line = element_line())+
   theme(panel.background = element_rect(fill = "white"),plot.margin =
margin(0.1, 0.1, 0.1, 0.1, "cm"), plot.background = element_rect(colour = NULL,
size = 1))+
   theme(axis.ticks.length.y = unit(.15, "cm"), axis.ticks.length.x =
```

```
unit(.25, "cm"), axis.text.x = element_text(margin = margin(t = .3, unit =
"cm")))+
   theme(legend.position = "right", legend.justification = "top", legend.direction =
"vertical", legend.text = element_text(size = 10))+
   theme(legend.key = element_rect(fill = "white"))+ theme(legend.title =
   element_text(face = NULL, size = 8, family =
"Arial"))+
   theme(panel.background = element_blank(), axis.text = element_blank())+
   theme(axis.text = element_text(colour = "black", size = 8, family = "Arial"))

#install.packages('extrafont')
library(extrafont) #font_import
#BiocManager::install("ggpubr")

p_root_20<-p_all
p_root_20

ggsave("C:/Users/icipe/Desktop/active_manuscript/soil/dada2_16s/
finale_results/abud/Top_20_Genus_Abundance_Root_Samples.jpeg",
           width = 18, height = 12, dpi = 600)

ggsave("C:/Users/icipe/Desktop/active_manuscript/soil/dada2_16s/finale
_results/abud/Top_20_Genus_Abundance_Root_Samples.png",
           width = 18, height = 12, dpi = 600)

ggsave("C:/Users/icipe/Desktop/active_manuscript/soil/dada2_16s/finale
_results/abud/Top_20_Genus_Abundance_Root_samples.svg", width
           = 18, height = 12, dpi = 600)

ggsave("C:/Users/icipe/Desktop/active_manuscript/soil/dada2_16s/finale
_results/abud/Top_20_Genus_Abundance_Root_samples.tiff", width
           = 18, height = 12, dpi = 600)
```

SOIL SAMPLES

```
#Groups the data in the defined order which eases downstream analysis group <-
Featured_table %>% group_by(Genus)%>%
   summarise_each(funs(sum),
"R1","R10","R11","R12","R13","R14","R15","R16","R17","R18","R19","R2", "R20",

"R3","R4","R5","R6","R7","R8","R9","S1","S10","S11","S12","S13","S14",
"S15","S16",
                          "S17","S18","S2","S3","S4","S5","S6","S7","S8","S9")
group<-group[-c(1),]
head(group)

S1_PS<-group[,c(1,22)]
```

```
S3_PS<-group[,c(1,33)]
S5_PS<-group[,c(1,35)]
S7_PS<-group[,c(1,37)]
S9_PS<-group[,c(1,39)]
S11_PS<-group[,c(1,24)]
S13_PS<-group[,c(1,26)]
S15_PS<-group[,c(1,28)]
S17_PS<-group[,c(1,30)]
S2_MS<-group[,c(1,32)]
S4_MS<-group[,c(1,34)]
S6_MS<-group[,c(1,36)]
S8_MS<-group[,c(1,38)]
S10_MS<-group[,c(1,23)]
S12_MS<-group[,c(1,25)]
S14_MS<-group[,c(1,27)]
S16_MS<-group[,c(1,29)]
S18_MS<-group[,c(1,31)]
```

SOIL SAMPLES DIVERSITY

```
S1_PS_total <- S1_PS%>% adorn_totals(c("col"))
S1_PS_total <- mutate(S1_PS_total, S1_PS=rowSums(S1_PS_total[3])/2)
S1_PS_total <- S1_PS_total[,c(1,4)]

S3_PS_total <- S3_PS%>% adorn_totals(c("col"))
S3_PS_total <- mutate(S3_PS_total, S3_PS=rowSums(S3_PS_total[3])/2)
S3_PS_total <- S3_PS_total[,c(1,4)]

S5_PS_total <- S5_PS%>% adorn_totals(c("col"))
S5_PS_total <- mutate(S5_PS_total, S5_PS=rowSums(S5_PS_total[3])/2)
S5_PS_total <- S5_PS_total[,c(1,4)]

S7_PS_total <- S7_PS%>% adorn_totals(c("col"))
S7_PS_total <- mutate(S7_PS_total, S7_PS=rowSums(S7_PS_total[3])/2)
S7_PS_total <- S7_PS_total[,c(1,4)]

S9_PS_total <- S9_PS%>% adorn_totals(c("col"))
S9_PS_total <- mutate(S9_PS_total, S9_PS=rowSums(S9_PS_total[3])/2)
S9_PS_total <- S9_PS_total[,c(1,4)]

S11_PS_total <- S11_PS%>% adorn_totals(c("col")) S11_PS_total
<- mutate(S11_PS_total, S11_PS=rowSums(S11_PS_total[3])/2)
S11_PS_total <- S11_PS_total[,c(1,4)]

S13_PS_total <- S13_PS%>% adorn_totals(c("col")) S13_PS_total
<- mutate(S13_PS_total, S13_PS=rowSums(S13_PS_total[3])/2)
S13_PS_total <- S13_PS_total[,c(1,4)]
```

```
S15_PS_total <- S15_PS%>% adorn_totals(c("col")) S15_PS_total
<- mutate(S15_PS_total, S15_PS=rowSums(S15_PS_total[3])/2)
S15_PS_total <- S15_PS_total[,c(1,4)]

S17_PS_total <- S17_PS%>% adorn_totals(c("col")) S17_PS_total
<- mutate(S17_PS_total, S17_PS=rowSums(S17_PS_total[3])/2)
S17_PS_total <- S17_PS_total[,c(1,4)]

S2_MS_total <- S2_MS%>% adorn_totals(c("col"))
S2_MS_total <- mutate(S2_MS_total, S2_MS=rowSums(S2_MS_total[3])/2)
S2_MS_total <- S2_MS_total[,c(1,4)]

S4_MS_total <- S4_MS%>% adorn_totals(c("col"))
S4_MS_total <- mutate(S4_MS_total, S4_MS=rowSums(S4_MS_total[3])/2)
S4_MS_total <- S4_MS_total[,c(1,4)]

S6_MS_total <- S6_MS%>% adorn_totals(c("col"))
S6_MS_total <- mutate(S6_MS_total, S6_MS=rowSums(S6_MS_total[3])/2)
S6_MS_total <- S6_MS_total[,c(1,4)]

S8_MS_total <- S8_MS%>% adorn_totals(c("col"))
S8_MS_total <- mutate(S8_MS_total, S8_MS=rowSums(S8_MS_total[3])/2)
S8_MS_total <- S8_MS_total[,c(1,4)]

S10_MS_total <- S10_MS%>% adorn_totals(c("col"))
S10_MS_total <- mutate(S10_MS_total,
S10_MS=rowSums(S10_MS_total[3])/2)
S10_MS_total <- S10_MS_total[,c(1,4)]

S12_MS_total <- S12_MS%>% adorn_totals(c("col"))
S12_MS_total <- mutate(S12_MS_total,
S12_MS=rowSums(S12_MS_total[3])/2)
S12_MS_total <- S12_MS_total[,c(1,4)]

S14_MS_total <- S14_MS%>% adorn_totals(c("col"))
S14_MS_total <- mutate(S14_MS_total,
S14_MS=rowSums(S14_MS_total[3])/2)
S14_MS_total <- S14_MS_total[,c(1,4)]

S16_MS_total <- S16_MS%>% adorn_totals(c("col"))
S16_MS_total <- mutate(S16_MS_total,
S16_MS=rowSums(S16_MS_total[3])/2)
S16_MS_total <- S16_MS_total[,c(1,4)]

S18_MS_total <- S18_MS%>% adorn_totals(c("col"))
S18_MS_total <- mutate(S18_MS_total,
```

```
S18_MS=rowSums(S18_MS_total[3])/2)
S18_MS_total <- S18_MS_total[,c(1,4)]

# Merging
merged <- Reduce(function(x,y) merge(x,y,by="Genus",all=TRUE),

list(S1_PS_total,S3_PS_total,S5_PS_total,S7_PS_total,S9_PS_total,S11_P S_total,

S13_PS_total,S15_PS_total,S17_PS_total,S2_MS_total,S4_MS_total,S6_MS_t otal,

S8_MS_total,S10_MS_total,S12_MS_total,S14_MS_total,S16_MS_total,S18_MS
_total))

names(merged)<-
c('Genus','S1_PS','S3_PS','S5_PS','S7_PS','S9_PS','S11_PS','S13_PS','S
15_PS','S17_PS','S2_MS',

'S4_MS','S6_MS','S8_MS','S10_MS','S12_MS','S14_MS','S16_MS','S18_MS')

#calculating the total abundance per genus and ordering from the most abundant to
the lowest
cumulation <- merged %>% adorn_totals(c("col"))
cumulation <- cumulation[order(cumulation$Total, decreasing = TRUE),]
cumulation$perc = cumulation$Total / sum(cumulation$Total) * 100

tired<-head(cumulation$Genus, n=20) #453 genus #
Using R base append() #install.packages('rlist')
library('rlist')
li2 <- append(tired,"Others") print(li2)
genus_Rep <- li2

group <- aggregate(merged[-1], list(Genus = replace(merged$Genus,!
(merged$Genus %in% genus_Rep), "Others")), sum)
#View(group)

dim(group)
All<- group[,c(1:19)]
```

Viewing Sample Diversity

```
#install.packages("janitor")
library(janitor)
#converting the abudances into percentage
bar_all <- adorn_percentages(All, denominator = "col", na.rm = T) bar_all %>%
```

```
   adorn_totals("row") %>%
   adorn_pct_formatting()
dist_all<-bar_all %>%
   adorn_totals("row") %>%
   adorn_pct_formatting()
dist_all write.csv(dist_all,
"C:/Users/icipe/Desktop/active_manuscript/soil/dada2_16s/finale_result
s/abud/Top_20_Abundance_Soil_Samples.csv")
#gathering the data
bar_all <- bar_all %>%
   gather(value = "abundance", key = "Farming_type", -Genus) bar_all <-
as.data.frame(gsub("\\(", " (", as.matrix(bar_all)))

# coerce the dataframe columns into respective data type
bar_all$Genus <- as.factor(bar_all$Genus)
bar_all$Farming_type<- as.character(bar_all$Farming_type)
bar_all$abundance <- as.numeric(bar_all$abundance)

#ordering the data for plotting
bar_all$Genus <- reorder(bar_all$Genus, bar_all$abundance) bar_all$Genus <-
factor(bar_all$Genus, levels=rev(levels(bar_all$Genus)))
bar_all$Genus <- factor(bar_all$Genus,
                                    levels=genus_Rep)

# Defining the color pallete
myPalette <- c("#1B9E77", "#D95F02", "#7570B3", "#E7298A", "#99000D",
"#E6AB02", "#A6761D", "#666666","#FDCDAC", "#1F78B4", "#B2DF8A",
"#33A02C", "#CBD5E8", "#E31A1C", "#FDBF6F",
"#FF7F00","#4A1486","#C0C0C0","#B3E2CD","#FFFF33",  "#5172b2","#F4CAE4",
"#E6F5C9", "#FCFBFD","#139BF1","#09FF00","#065535", "#1D91C0",
"#C0FFEE","#B35806","#0C2C84","#D0ED0E","#092617","#499976","#4D5D53",
"#E48400","#6082B6","#316689","#CEFB02","#738678","#645452","#EEA47FFF
", "#00539CFF", "#FC766AFF", "#42EADDFF", "#00A4CCFF", "#69B3BB",
"#B589D6","#D1DFB7","#97BC62FF","#D198C5FF","#000000", "#CBCE91FF",
"#616247FF", "#D64161FF","#435E55FF", "#DD4132FF","#CE4A7EFF",
"#BD7F37FF","#FFA351FF","#185E57", "#FCE3E3", "#EF6C6C", "#EF6C9A",
"#93103E", "#F7E3FC", "#D56CEF", "#BD19E6", "#8D6CEF", "#DBD1FA",
"#BFC8F8", "#1531BC", "#9FA5F3", "#95C7F3", "#6CEFC8", "#6CEF84",
"#91E619", "#B7CEEC", "#9AFEFF", "#57FEFF", "#78C7C7", "#46C7C7",
"#00A36C", "#728C00", "#4E9268", "#6CC417", "#64E986", "#F5E216",
"#FFCE44", "#8B8000", "#660000", "#610541", "#E56E94", "#F660AB",
"#E3319D", "#FF77FF", "#C45AEC", "#6960EC", "#736AFF", "#F9B7FF",
"#FCDFFF", "#D291BC", "#614051", "#FEA3AA", "#7D0541")

length(myPalette)

# Definig the names in italics
```

```
guide_italics <- guides(fill = guide_legend(label.theme = element_text(size = 15,
face = "italic", colour = "Black", angle = 0)))
```

Plotting barplot

```
library(Cairo)
library(forcats)

#plotting the barplot
p_all <- ggplot(bar_all,aes(x = fct_inorder(Farming_type), y = abundance),
labs(fill= Genus), group=row.names(bar_all))+ xlab("Root Sample")+
ylab("abundance") + geom_col(aes(fill = Genus),position = position_stack(reverse
= FALSE))+
   theme(axis.text.x = element_text(angle = 72,size = 15, hjust = 1, face =
"italic", family = "Arial"))+
     scale_fill_manual(values = myPalette)+ #guides(fill =
   guide_legend(reverse = FALSE))+ guide_italics+
   theme(legend.text = element_text(size = 8, colour = "black", face = "italic",
family = "Arial"), legend.text.align = 0)+
   theme(axis.text.y = element_text(angle = 0, vjust = 0.5, size = 8, family =
"Arial"))+
   theme(axis.text = element_text(colour = "black", size = 8, family = "Arial"))+
   theme(axis.line = element_line())+
   theme(panel.background = element_rect(fill = "white"),plot.margin =
margin(0.1, 0.1, 0.1, 0.1, "cm"), plot.background = element_rect(colour = NULL,
size = 1))+
   theme(axis.ticks.length.y = unit(.15, "cm"), axis.ticks.length.x = unit(.25, "cm"),
axis.text.x = element_text(margin = margin(t = .3, unit = "cm")))+
   theme(legend.position = "right", legend.justification = "top", legend.direction =
"vertical", legend.text = element_text(size = 10))+
   theme(legend.key = element_rect(fill = "white"))+ theme(legend.title =
   element_text(face = NULL, size = 8, family =
"Arial"))+
   theme(panel.background = element_blank(), axis.text = element_blank())+
   theme(axis.text = element_text(colour = "black", size = 8, family = "Arial"))

#install.packages('extrafont')
library(extrafont) #font_import
#BiocManager::install("ggpubr")

p_soil_20<-p_all p_soil_20
```

```
ggsave("C:/Users/icipe/Desktop/active_manuscript/soil/dada2_16s/
finale_results/abud/Top_20_Genus_Abundance_Soil_Samples.jpeg",
          width = 18, height = 12, dpi = 600)

ggsave("C:/Users/icipe/Desktop/active_manuscript/soil/dada2_16s/finale
_results/abud/Top_20_Genus_Abundance_Soil_Samples.png", width
          = 18, height = 12, dpi = 600)

ggsave("C:/Users/icipe/Desktop/active_manuscript/soil/dada2_16s/finale
_results/abud/Top_20_Genus_Abundance_Soil_samples.svg", width
          = 18, height = 12, dpi = 600)

ggsave("C:/Users/icipe/Desktop/active_manuscript/soil/dada2_16s/finale
_results/abud/Top_20_Genus_Abundance_Soil_samples.tiff", width
          = 18, height = 12, dpi = 600)
```

COMBININING: Root and Soil Samples

```
#p_root_20
#p_soil_20

p_root_20<-p_root_20 + stat_ellipse()  + labs(tag = "A")
p_soil_20<-p_soil_20 + stat_ellipse()  + labs(tag = "B")

ggarrange(p_root_20,p_soil_20,
              ncol = 1, nrow = 2) + geom_point()

ggsave("C:/Users/icipe/Desktop/active_manuscript/soil/dada2_16s/
finale_results/pub/Top_20_Bacteria_Genus_Samples.png",
          width = 18, height = 24, dpi = 600)

ggsave("C:/Users/icipe/Desktop/active_manuscript/soil/dada2_16s/finale
_results/pub/Top_20_Bacteria_Genus_Samples.svg", width =
          18, height = 24, dpi = 600)

ggsave("C:/Users/icipe/Desktop/active_manuscript/soil/dada2_16s/finale
_results/pub/Top_20_Bacteria_Genus_Samples.tiff", width =
          18, height = 24, dpi = 600)
ggsave("C:/Users/icipe/Desktop/active_manuscript/soil/dada2_16s/finale
_results/pub/Top_20_Bacteria_Genus_Samples.jpeg", width =
          18, height = 24, dpi = 600)
```

BETA DIVERSITY

```
#ordinating the phyloseq object
library(vegan)
library(ggpubr) #weighted
unifrac
Palette <- c('#89C5DA', "#DA5724", "#74D944", "#CE50CA", "#3F4921",
"#C0717C", "#CBD588", "#5F7FC7", "#673770", "#D3D93E", "#38333E",
"#508578", "#D7C1B1", "#689030", "#AD6F3B", "#CD9BCD", "#D14285",
```

```
"#6DDE88", "#652926", "#7FDCC0", "#C84248", "#8569D5", "#5E738F",
"#D1A33D", "#8A7C64", "#599861")

ordu = ordinate(ps3, "PCoA", "unifrac", weighted = TRUE) p <-
plot_ordination(ps3, ordu, color="Sample_type")+
geom_point(size=2) +
   scale_color_manual(values = Palette) + theme(axis.text.x =
   element_text(angle = 0, hjust = 1))+ theme(legend.justification =
   "top")+ theme(legend.position = "right")+
   theme(legend.key = element_rect(fill = "white"))+
   theme(legend.text = element_text(size = rel(1), colour = "black"))+
   theme(legend.title = element_text(face = NULL))
+theme(panel.background = element_blank(), axis.text = element_blank())+
   theme(axis.text = element_text(colour = "black", size = rel(1)))+ theme(axis.line
   = element_line())+
   theme(panel.background = element_rect(fill = "white"),plot.margin =
margin(0.5, 0.5, 0.5, 0.5, "cm"), plot.background = element_rect(colour =
"grey"))

bA1<-p + stat_ellipse()  + labs(tag = "A")


p <- plot_ordination(ps3, ordu, color="Farming_type")+ geom_point(size=2) +
   scale_color_manual(values = Palette) + theme(axis.text.x =
   element_text(angle = 0, hjust = 1))+ theme(legend.justification =
   "top")+ theme(legend.position = "right")+
   theme(legend.key = element_rect(fill = "white"))+
   theme(legend.text = element_text(size = rel(1), colour = "black"))+
   theme(legend.title = element_text(face = NULL))
+theme(panel.background = element_blank(), axis.text = element_blank())+
   theme(axis.text = element_text(colour = "black", size = rel(1)))+ theme(axis.line
   = element_line())+
   theme(panel.background = element_rect(fill = "white"),plot.margin =
margin(0.5, 0.5, 0.5, 0.5, "cm"), plot.background = element_rect(colour =
"grey"))

bB1<-p + stat_ellipse()  + labs(tag = "B")


p <- plot_ordination(ps3, ordu, color="Location")+ geom_point(size=2)
+
   scale_color_manual(values = Palette) + theme(axis.text.x =
   element_text(angle = 0, hjust = 1))+ theme(legend.justification =
   "top")+
```

```
   theme(legend.position = "right")+ theme(legend.key =
   element_rect(fill = "white"))+
   theme(legend.text = element_text(size = rel(1), colour = "black"))+
   theme(legend.title = element_text(face = NULL))
+theme(panel.background = element_blank(), axis.text = element_blank())+
   theme(axis.text = element_text(colour = "black", size = rel(1)))+ theme(axis.line
   = element_line())+
   theme(panel.background = element_rect(fill = "white"),plot.margin =
margin(0.5, 0.5, 0.5, 0.5, "cm"), plot.background = element_rect(colour =
"grey"))

bC1<-p + stat_ellipse()   + labs(tag = "C")


require(ggplot2) require(dplyr)
require(svglite) ggarrange(bA1,
bB1, bC1,
               ncol = 2, nrow = 2) + geom_point()




ggsave("C:/Users/icipe/Desktop/active_manuscript/soil/dada2_16s/finale
_results/pub/Beta_Diversity.jpeg",
          width = 12, height = 12, dpi = 600)

ggsave("C:/Users/icipe/Desktop/active_manuscript/soil/dada2_16s/finale
_results/pub/Beta_Diversity.png",
          width = 12, height = 12, dpi = 600)

ggsave("C:/Users/icipe/Desktop/active_manuscript/soil/dada2_16s/finale
_results/pub/Beta_Diversity.svg",
          width = 12, height = 12, dpi = 600)

ggsave("C:/Users/icipe/Desktop/active_manuscript/soil/dada2_16s/finale
_results/pub/Beta_Diversity.tiff",
          width = 12, height = 12, dpi = 600)

#Beta diversity estimation #Drawing
the venn diagrams
#Removing all genuses with zero hits in the dataframes
#install.packages("ggpolypath")            # Install ggpolypath package
library("ggpolypath")
library(ggvenn)
library(ggVennDiagram)
#Sample Type soil_total
```

```
root_total

Soil<-soil_total[!(soil_total$soil == 0),]
Root<-root_total[!(root_total$root == 0),] #remove
NA genus

#
Soil<-Soil %>% select(Genus)
Root<-Root %>% select(Genus)


# Farming type
ps_total
head(ms_total)
pr_total mr_total

PSV<-ps_total[!(ps_total$ps == 0),]
MSV<-ms_total[!(ms_total$ms == 0),]
PRV<-pr_total[!(pr_total$pr == 0),]
MRV<-mr_total[!(mr_total$mr == 0),] #remove

NA genus

PSV<-PSV                    %>%
select(Genus)        MSV<-MSV
%>%   select(Genus)   PRV<-
PRV     %>%     select(Genus)
MRV<-MRV                    %>%
select(Genus)

# Location
head(bungoma_total)
bungomav<-bungoma_total[!(bungoma_total$bungoma == 0),]
siayav<-siaya_total[!(siaya_total$siaya == 0),]
vihigav<-vihiga_total[!(vihiga_total$vihiga == 0),] #remove NA
genus

bungomav<-PSV %>%
select(Genus) siayav<-MSV %>%
select(Genus) vihigav<-PRV %>%
select(Genus)

library(gplots)
vd <- list(bungomav, siayav, vihiga) names(vd) =
c("Bungoma", "Siaya", "Vihiga") venn(data=vd)
ggVennDiagram(vd)
ggvenn(vd)
```

VENN DIAGRAM

SAMPLE TYPE

Venn or Upset plot

```
vennlist <- get_vennlist(obj=ps3,
                         factorNames="Sample_type")
vennp <- venn.diagram(vennlist,
                      height=5,
                      width=5,
                      filename=NULL,
                      fill=c("#89C5DA", "#DA5724"), cat.col=c("#89C5DA",
                     "#DA5724"),
                      alpha = 0.85, fontfamily
                      = "serif", fontface =
                      "bold", cex = 1.2,
                      cat.cex = 1.3, cat.default.pos =
                      "outer", cat.dist=0.1,
                      margin = 0.1,
                      lwd = 3,
                     # lty ='dotted', imagetype =
                      "svg")
grid::grid.draw(vennp)



png("C:/Users/icipe/Desktop/active_manuscript/soil/dada2_16s/
finale_results/venn/1.venn_sample_type.png",width = 1000, height = 600, units
= "px")
grid::grid.draw(vennp)
while (!is.null(dev.list()))  dev.off()

upsetda <- get_upset(obj=ps3,
                     factorNames="Sample_type")
png("C:/Users/icipe/Desktop/active_manuscript/soil/dada2_16s/finale_re
sults/venn/2.venn_sample_type.png",width = 1000, height = 600, units = "px")
upset(upsetda, sets=unique(as.vector(sample_data(ps3)$Sample_type)),
      sets.bar.color = "#56B4E9",
      order.by = "freq", empty.intersections =
      "on")
while (!is.null(dev.list()))  dev.off()
```

FARMING TYPE

Venn or Upset plot

```
vennlist <- get_vennlist(obj=ps3,
                         factorNames="Farming_type")
vennp <- venn.diagram(vennlist,
                      height=5,
                      width=5,
                      filename=NULL,
                      fill=c("#89C5DA", "#DA5724"), cat.col=c("#89C5DA",
                     "#DA5724"),
                      alpha = 0.85, fontfamily
                      = "serif", fontface =
                      "bold", cex = 1.2,
                      cat.cex = 1.3, cat.default.pos =
                      "outer", cat.dist=0.1,
                      margin = 0.1,
                      lwd = 3,
                     # lty ='dotted', imagetype =
                      "svg")
grid::grid.draw(vennp)

png("C:/Users/icipe/Desktop/active_manuscript/soil/dada2_16s/
finale_results/venn/1.venn_farming_type.png",width = 1000, height = 600, units
= "px")
grid::grid.draw(vennp)
while (!is.null(dev.list()))  dev.off()

upsetda <- get_upset(obj=ps3,
                     factorNames="Sample_type")
png("C:/Users/icipe/Desktop/active_manuscript/soil/dada2_16s/finale_re
sults/venn/2.venn_farming_type.png",width = 1000, height = 600, units
= "px")
upset(upsetda, sets=unique(as.vector(sample_data(ps3)$Sampling_type)),

      sets.bar.color = "#56B4E9",
      order.by = "freq",
      empty.intersections = "on")
while (!is.null(dev.list()))  dev.off()
```

LOCATION

Venn or Upset plot

```
vennlist <- get_vennlist(obj=ps3,
                         factorNames="Location") vennp
<- venn.diagram(vennlist,
```

```
                                height=5,
                                width=5,
                                filename=NULL,
                                fill=c("#89C5DA", "#DA5724","#0071BC"),
                               cat.col=c("#89C5DA", "#DA5724","#0071BC"),
                                alpha = 0.85, fontfamily
                                = "serif", fontface =
                                "bold", cex = 1.2,
                                cat.cex = 1.3, cat.default.pos =
                                "outer", cat.dist=0.1,
                                margin = 0.1,
                                lwd = 3,
                               # lty ='dotted', imagetype =
                                "svg")
grid::grid.draw(vennp)

png("C:/Users/icipe/Desktop/active_manuscript/soil/dada2_16s/
finale_results/venn/1.venn_Location.png",width = 1000, height = 600, units = "px")
grid::grid.draw(vennp)
while (!is.null(dev.list()))  dev.off()

upsetda <- get_upset(obj=ps3,
                               factorNames="Location")
png("C:/Users/icipe/Desktop/active_manuscript/soil/dada2_16s/finale_re
sults/venn/2.venn_Location.png",width = 1000, height = 600, units = "px")
upset(upsetda, sets=unique(as.vector(sample_data(ps3)$Location)), sets.bar.color
      = "#56B4E9",
      order.by = "freq", empty.intersections =
      "on")
while (!is.null(dev.list()))  dev.off()
```

ALPHA DIVERSITY

```
ps3
set.seed(1024)
rareres <- get_rarecurve(obj=ps3, chunks=400)

p_rare <- ggrarecurve(obj=rareres,
                                indexNames=c("Observe","Chao1","ACE","Shannon"),
                                ) +
          theme(legend.spacing.y=unit(0.01,"cm"),
                legend.text=element_text(size=4))

prare1 <- ggrarecurve(obj=rareres, factorNames="Sample_type",
                                indexNames=c("Observe","Chao1","ACE","Shannon")
                                ) +
```

```
            scale_fill_manual(values=c("#89C5DA", "#DA5724", "#74D944",
"#CE50CA"))+
            scale_color_manual(values=c("#89C5DA", "#DA5724", "#74D944",
"#CE50CA"))+
            theme_bw()+ theme(axis.text=element_text(size=8),
panel.grid=element_blank(),
                    strip.background = element_rect(colour=NA,fill="grey"),
                    strip.text.x = element_text(face="bold"))

prare2 <- ggrarecurve(obj=rareres,
                          factorNames="Sample_type",
                          shadow=FALSE,
                          indexNames=c("Observe", "Chao1",
"ACE","Shannon")
                          ) +
            scale_color_manual(values=c("#89C5DA", "#DA5724", "#74D944",
"#CE50CA"))+
            theme_bw()+ theme(axis.text=element_text(size=8),
panel.grid=element_blank(),
                    strip.background = element_rect(colour=NA,fill="grey"),
                    strip.text.x = element_text(face="bold")) p_rare
/ prare1 / prare2

alphaobj <- get_alphaindex(ps3)
head(as.data.frame(alphaobj))

p_alpha_ty <-
ggbox(alphaobj,indexNames=c("Chao1","Observe","Shannon","Simpson"),
factorNames="Sample_type") +
             scale_fill_manual(values=c("#89C5DA", "#DA5724", "#74D944",
"#CE50CA"))+
             theme(strip.background = element_rect(colour=NA, fill="grey"))
p_alpha_ty

ggsave("C:/Users/icipe/Desktop/active_manuscript/soil/dada2_16s/
finale_results/alpha/Alpha_Diversity_Sample_Type.jpeg",
          width = 18, height = 12, dpi = 600)

ggsave("C:/Users/icipe/Desktop/active_manuscript/soil/dada2_16s/finale
_results/alpha/Alpha_Diversity_Sample_Type.png", width =
          18, height = 12, dpi = 600)

ggsave("C:/Users/icipe/Desktop/active_manuscript/soil/dada2_16s/finale
_results/alpha/Alpha_Diversity_Sample_Type.svg", width =
          18, height = 12, dpi = 600)
```

```
ggsave("C:/Users/icipe/Desktop/active_manuscript/soil/dada2_16s/finale
_results/alpha/Alpha_Diversity_Sample_Type.tiff", width =
          18, height = 12, dpi = 600)

alphaobj <- get_alphaindex(ps3)
head(as.data.frame(alphaobj))

p_alpha_lc <- ggbox(alphaobj,
indexNames=c("Chao1","Observe","Shannon","Simpson"), factorNames="Location")
+
                scale_fill_manual(values=c("#89C5DA", "#DA5724", "#74D944",
"#CE50CA"))+
                theme(strip.background = element_rect(colour=NA, fill="grey"))
p_alpha_lc

ggsave("C:/Users/icipe/Desktop/active_manuscript/soil/dada2_16s/
finale_results/alpha/Alpha_Diversity_Location.jpeg",
          width = 18, height = 12, dpi = 600)

ggsave("C:/Users/icipe/Desktop/active_manuscript/soil/dada2_16s/finale
_results/alpha/Alpha_Diversity_Location.png", width
          = 18, height = 12, dpi = 600)

ggsave("C:/Users/icipe/Desktop/active_manuscript/soil/dada2_16s/finale
_results/alpha/Alpha_Diversity_Location.svg", width
          = 18, height = 12, dpi = 600)

ggsave("C:/Users/icipe/Desktop/active_manuscript/soil/dada2_16s/finale
_results/alpha/Alpha_Diversity_Location.tiff", width =
          18, height = 12, dpi = 600)

alphaobj <- get_alphaindex(ps3)
head(as.data.frame(alphaobj))

p_alpha_ft <- ggbox(alphaobj,
indexNames=c("Chao1","Observe","Shannon","Simpson"),
factorNames="Farming_type") +
                scale_fill_manual(values=c("#89C5DA", "#DA5724", "#74D944",
"#CE50CA"))+
                theme(strip.background = element_rect(colour=NA, fill="grey"))
p_alpha_ft

ggsave("C:/Users/icipe/Desktop/active_manuscript/soil/dada2_16s/
finale_results/alpha/Alpha_Diversity_Farming_Type.jpeg",
          width = 18, height = 12, dpi = 600)

ggsave("C:/Users/icipe/Desktop/active_manuscript/soil/dada2_16s/finale
```

```
_results/alpha/Alpha_Diversity_Farming_Type.png", width =
        18, height = 12, dpi = 600)

ggsave("C:/Users/icipe/Desktop/active_manuscript/soil/dada2_16s/finale
_results/alpha/Alpha_Diversity_Farming_Type.svg", width =
        18, height = 12, dpi = 600)

ggsave("C:/Users/icipe/Desktop/active_manuscript/soil/dada2_16s/finale
_results/alpha/Alpha_Diversity_Farming_Type.tiff", width =
        18, height = 12, dpi = 600)


p_alpha_ty<-p_alpha_ty + stat_ellipse() + labs(tag = "A")
p_alpha_ft<-p_alpha_ft + stat_ellipse() + labs(tag = "B")
p_alpha_lc<-p_alpha_lc + stat_ellipse()  + labs(tag = "C")

ggarrange(p_alpha_ty,  p_alpha_ft, p_alpha_lc, ncol =
            1, nrow = 3) + geom_point()


ggsave("C:/Users/icipe/Desktop/active_manuscript/soil/dada2_16s/finale
_results/pub/Combined_Alpha_Diversity.jpeg", width
        = 12, height = 36, dpi = 600)

ggsave("C:/Users/icipe/Desktop/active_manuscript/soil/dada2_16s/finale
_results/pub/Combined_Alpha_Diversity.png", width
        = 12, height = 36, dpi = 600)

ggsave("C:/Users/icipe/Desktop/active_manuscript/soil/dada2_16s/finale
_results/pub/Combined_Alpha_Diversity.svg", width
        = 12, height = 36, dpi = 600)

ggsave("C:/Users/icipe/Desktop/active_manuscript/soil/dada2_16s/finale
_results/pub/Combined_Alpha_Diversity.tiff", width =
        12, height = 36, dpi = 600)

###ALPHA PART TWO



#alpha diversity estimation physeq4
<- ps3
physeq4

#checking out the total read counts in the samples reads <-
sample_sums(physeq4)
reads

summary(sample_sums(physeq4))
```

```
library(microbiome)
#Extracting the otu table from the phyloseq object and plotting the rarefaction
curve
otu_tab <- t(abundances(physeq4)) p
<- vegan::rarecurve(otu_tab,
                         step = 50, label = FALSE, sample =
                         min(rowSums(otu_tab),
                                        col = "blue", cex = 0.6))

set.seed(9242)

#calculatin an even sampling depth for all the samples rarefied <-
rarefy_even_depth(physeq4, sample.size = 38) rarefied

#calculating the alpha diversity
diversity <- alpha(rarefied, index = "all")
diversity <- rownames_to_column(diversity, "sample_id")

#Extracting the sample metadata from the phyloseq object sdata1
<- meta(physeq4)
sdata1 <- rownames_to_column(sdata1, "sample_id")
```

Sample Type

CHAO1

```
#Chao1 diversity estimates
chao1 <- diversity %>% select(sample_id, chao1)
chao_edited <- merge(chao1, sdata1, by = "sample_id", all = TRUE) #chao_edited
<- chao_edited[c(1:18, 62:86, 19:61),]

#confirming if the chao1 indices are normally distributed
shapiro.test(chao_edited$chao1)

#plotting chao1 distribution boxplot
Pchao <- ggboxplot(chao_edited, "Sample_type","chao1",
                 color = "Sample_type", palette =c("#FBAE17", "#F52100",
"#0071BC"),
                 add = "jitter", linetype = "solid", Family = "Palatino
Linotype", add.params = list(),
                 error.plot = "pointrange", legand = NULL, size = NULL, width =
0.7, notch = FALSE, outlier.shape = 20, facet.by = NULL,
                 panel.labs = NULL, short.panel.labs = TRUE,bxp.errorbar
= FALSE, bxp.errorbar.width = 0.4, ggtheme = theme_pubr())+ theme(axis.text.x
   = element_text(angle = 0, hjust = 0.5)) + theme(legend.text =
   element_text(size = 10, colour = "black", face =
"italic"), legend.text.align = 0)+
```

```
   theme(axis.text.y = element_text(angle = 0, vjust = 0.5, size = 10))
+
   theme(axis.text = element_text(colour = "black", size = 10))+ theme(axis.line =
   element_line())+
   theme(panel.background = element_rect(fill = "white"),plot.margin =
margin(0.5, 0.5, 0.5, 0.5, "cm"), plot.background = element_rect(colour = NULL,
size = 1))+
   theme(axis.ticks.length.y = unit(.25, "cm"), axis.ticks.length.x = unit(.25, "cm"),
axis.text.x = element_text(margin = margin(t = .3, unit = "cm")))+
   theme(legend.justification = "top")+
   theme(legend.position = "right")+ theme(legend.key =
   element_rect(fill = "white"))+
   theme(legend.title = element_text(face = NULL, size = 10))
+theme(panel.background = element_blank(), axis.text = element_blank())+
   theme(axis.text = element_text(colour = "black", size = 10)+
            theme(axis.line = element_line())+ theme(panel.background =
            element_rect(fill =
"white"),plot.margin = margin(0.5, 0.5, 0.5, 0.5, "cm"), plot.background =
element_rect(colour = "grey"))+
            theme(axis.ticks.length.y = unit(.25, "cm"), axis.ticks.length.x =
unit(.25, "cm"), axis.text.x = element_text(margin = margin(t = .3, unit =
"cm")))+
            theme(axis.title.y = element_text(size = 10, face = "plain", angle =
90))+
            theme(axis.title.x = element_text(size = 10, angle =
0)))#+stat_compare_means()
Pchao<-Pchao + aes(x = fct_inorder(Sample_type)) + theme(legend.position =
"none") + xlab("Sample Type") + ylab("Chao1") Pchao
```

OBSERVED

```
#Chao1 diversity estimates
observed <- diversity %>% select(sample_id, observed) observed_edited <-
merge(observed, sdata1, by = "sample_id", all = TRUE)
#chao_edited <- chao_edited[c(1:18, 62:86, 19:61),]

#confirming if the chao1 indices are normally distributed
shapiro.test(observed_edited$observed)

#plotting chao1 distribution boxplot
Pobs <- ggboxplot(observed_edited, "Sample_type","observed",
                  color = "Sample_type", palette =c("#FBAE17", "#F52100",
"#0071BC"),
                  add = "jitter", linetype = "solid", Family = "Palatino
Linotype", add.params = list(),
                  error.plot = "pointrange", legand = NULL, size = NULL,
```

```
width = 0.7, notch = FALSE, outlier.shape = 20, facet.by = NULL, panel.labs =
                        NULL, short.panel.labs = TRUE,bxp.errorbar
= FALSE, bxp.errorbar.width = 0.4, ggtheme = theme_pubr())+ theme(axis.text.x
   = element_text(angle = 0, hjust = 0.5)) + theme(legend.text =
   element_text(size = 10, colour = "black", face =
"italic"), legend.text.align = 0)+
   theme(axis.text.y = element_text(angle = 0, vjust = 0.5, size = 10))
+
   theme(axis.text = element_text(colour = "black", size = 10))+ theme(axis.line =
   element_line())+
   theme(panel.background = element_rect(fill = "white"),plot.margin =
margin(0.5, 0.5, 0.5, 0.5, "cm"), plot.background = element_rect(colour = NULL,
size = 1))+
   theme(axis.ticks.length.y = unit(.25, "cm"), axis.ticks.length.x = unit(.25, "cm"),
axis.text.x = element_text(margin = margin(t = .3, unit = "cm")))+
   theme(legend.justification = "top")+
   theme(legend.position = "right")+ theme(legend.key =
   element_rect(fill = "white"))+
   theme(legend.title = element_text(face = NULL, size = 10))
+theme(panel.background = element_blank(), axis.text = element_blank())+
   theme(axis.text = element_text(colour = "black", size = 10)+
                theme(axis.line = element_line())+ theme(panel.background =
                element_rect(fill =
"white"),plot.margin = margin(0.5, 0.5, 0.5, 0.5, "cm"), plot.background =
element_rect(colour = "grey"))+
                theme(axis.ticks.length.y = unit(.25, "cm"), axis.ticks.length.x =
unit(.25, "cm"), axis.text.x = element_text(margin = margin(t = .3, unit =
"cm")))+
                theme(axis.title.y = element_text(size = 10, face = "plain", angle =
90))+
                theme(axis.title.x = element_text(size = 10, angle = 0)))#+
stat_compare_means()


Pobs<-Pobs + aes(x = fct_inorder(Sample_type)) + theme(legend.position
= "none") + xlab("Sample Type") + ylab("Observed") Pobs
```

SHANNON DIVERSITY

```
#Extracting the shannon diversity index
shannon <- diversity %>% select(sample_id, diversity_shannon)

shannon_edited <- merge(shannon, sdata1, by = "sample_id", all = TRUE)
#shannon_edited <- shannon_edited[c(1:18, 62:86, 19:61),]

#confirming if the shannon indices are normally distributed
shapiro.test(shannon_edited$diversity_shannon)
```

```
library(ggpubr)


#plotting the boxplots for the shannon index data
P <- ggboxplot(shannon_edited, "Sample_type","diversity_shannon", color =
                        "Sample_type", palette =c("#FBAE17", "#F52100",
"#0071BC"),
                        add = "jitter", linetype = "solid", Family = "Palatino
Linotype", add.params = list(),
                        error.plot = "pointrange", legand = NULL, size = NULL, width =
0.7, notch = FALSE, outlier.shape = 20, facet.by = NULL,
                        panel.labs = NULL, short.panel.labs = TRUE,bxp.errorbar
= FALSE, bxp.errorbar.width = 0.4, ggtheme = theme_pubr())+ theme(axis.text.x
   = element_text(angle = 0, hjust = 0.5))+ theme(legend.text =
   element_text(size = 10, colour = "black", face =
"italic"), legend.text.align = 0)+
   theme(axis.text.y = element_text(angle = 0, vjust = 0.5, size = 10))
+
   theme(axis.text = element_text(colour = "black", size = 10))+ theme(axis.line =
   element_line())+
   theme(panel.background = element_rect(fill = "white"),plot.margin =
margin(0.5, 0.5, 0.5, 0.5, "cm"), plot.background = element_rect(colour = NULL,
size = 1))+
   theme(axis.ticks.length.y = unit(.25, "cm"), axis.ticks.length.x = unit(.25, "cm"),
axis.text.x = element_text(margin = margin(t = .3, unit = "cm")))+
   theme(legend.justification = "top")+
   theme(legend.position = "right")+ theme(legend.key =
   element_rect(fill = "white"))+
   theme(legend.title = element_text(face = NULL, size = 10))
+theme(panel.background = element_blank(), axis.text = element_blank())+
   theme(axis.text = element_text(colour = "black", size = 10)+
               theme(axis.line = element_line())+ theme(panel.background =
               element_rect(fill =
"white"),plot.margin = margin(0.5, 0.5, 0.5, 0.5, "cm"), plot.background =
element_rect(colour = "grey"))+
               theme(axis.ticks.length.y = unit(.25, "cm"), axis.ticks.length.x =
unit(.25, "cm"), axis.text.x = element_text(margin = margin(t = .3, unit =
"cm")))+
               theme(axis.title.y = element_text(size = 10, face = "plain", angle =
90))+
               theme(axis.title.x = element_text(size = 10, angle = 0)))
#+stat_compare_means()

Pshan<-P + aes(x = fct_inorder(Sample_type)) + theme(legend.position = "none")
+ xlab("Sample Type") + ylab("Shannon")

Pshan
```

Evenness

```
#Extracting the shannon diversity index
even <- diversity %>% select(sample_id, evenness_pielou)

even_edited <- merge(even, sdata1, by = "sample_id", all = TRUE)
#shannon_edited <- shannon_edited[c(1:18, 62:86, 19:61),]

#confirming if the shannon indices are normally distributed
shapiro.test(even_edited$evenness_pielou)

library(ggpubr)



#plotting the boxplots for the shannon index data
P <- ggboxplot(even_edited, "Sample_type","evenness_pielou",
                        color = "Sample_type", palette =c("#FBAE17", "#F52100",
"#0071BC"),
                        add = "jitter", linetype = "solid", Family = "Palatino
Linotype", add.params = list(),
                        error.plot = "pointrange", legand = NULL, size = NULL, width =
0.7, notch = FALSE, outlier.shape = 20, facet.by = NULL,
                        panel.labs = NULL, short.panel.labs = TRUE,bxp.errorbar
= FALSE, bxp.errorbar.width = 0.4, ggtheme = theme_pubr())+ theme(axis.text.x
   = element_text(angle = 0, hjust = 0.5))+ theme(legend.text =
   element_text(size = 10, colour = "black", face =
"italic"), legend.text.align = 0)+
   theme(axis.text.y = element_text(angle = 0, vjust = 0.5, size = 10))
+
   theme(axis.text = element_text(colour = "black", size = 10))+ theme(axis.line =
   element_line())+
   theme(panel.background = element_rect(fill = "white"),plot.margin =
margin(0.5, 0.5, 0.5, 0.5, "cm"), plot.background = element_rect(colour = NULL,
size = 1))+
   theme(axis.ticks.length.y = unit(.25, "cm"), axis.ticks.length.x = unit(.25, "cm"),
axis.text.x = element_text(margin = margin(t = .3, unit = "cm")))+
   theme(legend.justification = "top")+
   theme(legend.position = "right")+ theme(legend.key =
   element_rect(fill = "white"))+
   theme(legend.title = element_text(face = NULL, size = 10))
+theme(panel.background = element_blank(), axis.text = element_blank())+
   theme(axis.text = element_text(colour = "black", size = 10)+
               theme(axis.line = element_line())+ theme(panel.background =
               element_rect(fill =
"white"),plot.margin = margin(0.5, 0.5, 0.5, 0.5, "cm"), plot.background =
element_rect(colour = "grey"))+
               theme(axis.ticks.length.y = unit(.25, "cm"), axis.ticks.length.x =
unit(.25, "cm"), axis.text.x =
```

```
element_text(margin = margin(t = .3, unit = "cm")))+ theme(axis.title.y =
            element_text(size = 10, face = "plain",
angle = 90))+
            theme(axis.title.x = element_text(size = 10, angle = 0)))
#+stat_compare_means()

Peven<-P + aes(x = fct_inorder(Sample_type)) + theme(legend.position = "none")
+ xlab("Sample Type") + ylab("Evenness")

Peven
```

LOCATION

CHAO1

```
#Chao1 diversity estimates
chao1 <- diversity %>% select(sample_id, chao1)
chao_edited <- merge(chao1, sdata1, by = "sample_id", all = TRUE) #chao_edited
<- chao_edited[c(1:18, 62:86, 19:61),]

#confirming if the chao1 indices are normally distributed
shapiro.test(chao_edited$chao1)

#plotting chao1 distribution boxplot
Pchao_l <- ggboxplot(chao_edited, "Location","chao1",
                color = "Location", palette =c("#FBAE17", "#F52100",
"#0071BC"),
                add = "jitter", linetype = "solid", Family = "Palatino
Linotype", add.params = list(),
                error.plot = "pointrange", legand = NULL, size = NULL, width =
0.7, notch = FALSE, outlier.shape = 20, facet.by = NULL,
                panel.labs = NULL, short.panel.labs = TRUE,bxp.errorbar
= FALSE, bxp.errorbar.width = 0.4, ggtheme = theme_pubr())+ theme(axis.text.x
  = element_text(angle = 0, hjust = 0.5)) + theme(legend.text =
  element_text(size = 10, colour = "black", face =
"italic"), legend.text.align = 0)+
  theme(axis.text.y = element_text(angle = 0, vjust = 0.5, size = 10))
+
  theme(axis.text = element_text(colour = "black", size = 10))+ theme(axis.line =
  element_line())+
  theme(panel.background = element_rect(fill = "white"),plot.margin =
margin(0.5, 0.5, 0.5, 0.5, "cm"), plot.background = element_rect(colour = NULL,
size = 1))+
  theme(axis.ticks.length.y = unit(.25, "cm"), axis.ticks.length.x = unit(.25, "cm"),
axis.text.x = element_text(margin = margin(t = .3, unit = "cm")))+
  theme(legend.justification = "top")+
  theme(legend.position = "right")+ theme(legend.key =
  element_rect(fill = "white"))+
```

```
   theme(legend.title = element_text(face = NULL, size = 10))
+theme(panel.background = element_blank(), axis.text = element_blank())+
   theme(axis.text = element_text(colour = "black", size = 10)+
               theme(axis.line = element_line())+ theme(panel.background =
               element_rect(fill =
"white"),plot.margin = margin(0.5, 0.5, 0.5, 0.5, "cm"), plot.background =
element_rect(colour = "grey"))+
               theme(axis.ticks.length.y = unit(.25, "cm"), axis.ticks.length.x =
unit(.25, "cm"), axis.text.x = element_text(margin = margin(t = .3, unit =
"cm")))+
               theme(axis.title.y = element_text(size = 10, face = "plain", angle =
90))+
               theme(axis.title.x = element_text(size = 10, angle =
0)))#+stat_compare_means()
Pchao_l<-Pchao_l + aes(x = fct_inorder(Location)) + theme(legend.position =
"none") + xlab("Location") + ylab("Chao1") Pchao_l
```

OBSERVED

```
#Chao1 diversity estimates
observed <- diversity %>% select(sample_id, observed) observed_edited <-
merge(observed, sdata1, by = "sample_id", all = TRUE)
#chao_edited <- chao_edited[c(1:18, 62:86, 19:61),]

#confirming if the chao1 indices are normally distributed
shapiro.test(observed_edited$observed)

#plotting chao1 distribution boxplot
Pobs_l <- ggboxplot(observed_edited, "Location","observed",
                       color = "Location", palette =c("#FBAE17", "#F52100",
"#0071BC"),
                       add = "jitter", linetype = "solid", Family = "Palatino
Linotype", add.params = list(),
                       error.plot = "pointrange", legand = NULL, size = NULL, width =
0.7, notch = FALSE, outlier.shape = 20, facet.by = NULL,
                       panel.labs = NULL, short.panel.labs = TRUE,bxp.errorbar
= FALSE, bxp.errorbar.width = 0.4, ggtheme = theme_pubr())+ theme(axis.text.x
   = element_text(angle = 0, hjust = 0.5)) + theme(legend.text =
   element_text(size = 10, colour = "black", face =
"italic"), legend.text.align = 0)+
   theme(axis.text.y = element_text(angle = 0, vjust = 0.5, size = 10))
+
   theme(axis.text = element_text(colour = "black", size = 10))+ theme(axis.line =
   element_line())+
   theme(panel.background = element_rect(fill = "white"),plot.margin =
margin(0.5, 0.5, 0.5, 0.5, "cm"), plot.background = element_rect(colour = NULL,
size = 1))+
```

```
   theme(axis.ticks.length.y = unit(.25, "cm"), axis.ticks.length.x = unit(.25, "cm"),
axis.text.x = element_text(margin = margin(t = .3, unit = "cm")))+
   theme(legend.justification = "top")+
   theme(legend.position = "right")+ theme(legend.key =
   element_rect(fill = "white"))+
   theme(legend.title = element_text(face = NULL, size = 10))
+theme(panel.background = element_blank(), axis.text = element_blank())+
   theme(axis.text = element_text(colour = "black", size = 10)+
               theme(axis.line = element_line())+ theme(panel.background =
               element_rect(fill =
"white"),plot.margin = margin(0.5, 0.5, 0.5, 0.5, "cm"), plot.background =
element_rect(colour = "grey"))+
               theme(axis.ticks.length.y = unit(.25, "cm"), axis.ticks.length.x =
unit(.25, "cm"), axis.text.x = element_text(margin = margin(t = .3, unit =
"cm")))+
               theme(axis.title.y = element_text(size = 10, face = "plain", angle =
90))+
               theme(axis.title.x = element_text(size = 10, angle = 0)))#+
stat_compare_means()


Pobs_l<-Pobs_l + aes(x = fct_inorder(Location)) + theme(legend.position =
"none") + xlab("Location") + ylab("Observed")

Pobs_l
```

SHANNON DIVERSITY

```
#Extracting the shannon diversity index
shannon <- diversity %>% select(sample_id, diversity_shannon)

shannon_edited <- merge(shannon, sdata1, by = "sample_id", all = TRUE)
#shannon_edited <- shannon_edited[c(1:18, 62:86, 19:61),]

#confirming if the shannon indices are normally distributed
shapiro.test(shannon_edited$diversity_shannon)

library(ggpubr)



#plotting the boxplots for the shannon index data
P_l <- ggboxplot(shannon_edited, "Location","diversity_shannon", color =
                       "Location", palette =c("#FBAE17", "#F52100",
"#0071BC"),
                       add = "jitter", linetype = "solid", Family = "Palatino
Linotype", add.params = list(),
                       error.plot = "pointrange", legand = NULL, size = NULL, width =
0.7, notch = FALSE, outlier.shape = 20, facet.by = NULL,
```

```
                    panel.labs = NULL, short.panel.labs = TRUE,bxp.errorbar
= FALSE, bxp.errorbar.width = 0.4, ggtheme = theme_pubr())+ theme(axis.text.x
  = element_text(angle = 0, hjust = 0.5))+ theme(legend.text =
  element_text(size = 10, colour = "black", face =
"italic"), legend.text.align = 0)+
  theme(axis.text.y = element_text(angle = 0, vjust = 0.5, size = 10))
+
  theme(axis.text = element_text(colour = "black", size = 10))+ theme(axis.line =
  element_line())+
  theme(panel.background = element_rect(fill = "white"),plot.margin =
margin(0.5, 0.5, 0.5, 0.5, "cm"), plot.background = element_rect(colour = NULL,
size = 1))+
  theme(axis.ticks.length.y = unit(.25, "cm"), axis.ticks.length.x = unit(.25, "cm"),
axis.text.x = element_text(margin = margin(t = .3, unit = "cm")))+
  theme(legend.justification = "top")+
  theme(legend.position = "right")+ theme(legend.key =
  element_rect(fill = "white"))+
  theme(legend.title = element_text(face = NULL, size = 10))
+theme(panel.background = element_blank(), axis.text = element_blank())+
  theme(axis.text = element_text(colour = "black", size = 10)+
            theme(axis.line = element_line())+ theme(panel.background =
            element_rect(fill =
"white"),plot.margin = margin(0.5, 0.5, 0.5, 0.5, "cm"), plot.background =
element_rect(colour = "grey"))+
            theme(axis.ticks.length.y = unit(.25, "cm"), axis.ticks.length.x =
unit(.25, "cm"), axis.text.x = element_text(margin = margin(t = .3, unit =
"cm")))+
            theme(axis.title.y = element_text(size = 10, face = "plain", angle =
90))+
            theme(axis.title.x = element_text(size = 10, angle = 0)))
#+stat_compare_means()

Pshan_l<-P_l + aes(x = fct_inorder(Location)) + theme(legend.position
= "none") + xlab("Location") + ylab("Shannon")

Pshan_l
```

Evenness

```
#Extracting the shannon diversity index
even <- diversity %>% select(sample_id, evenness_pielou)

even_edited <- merge(even, sdata1, by = "sample_id", all = TRUE)
#shannon_edited <- shannon_edited[c(1:18, 62:86, 19:61),]

#confirming if the shannon indices are normally distributed
shapiro.test(even_edited$evenness_pielou)

library(ggpubr)
```

```
#plotting the boxplots for the shannon index data
Pe_l <- ggboxplot(even_edited, "Location","evenness_pielou",
                        color = "Location", palette =c("#FBAE17", "#F52100",
"#0071BC"),
                        add = "jitter", linetype = "solid", Family = "Palatino
Linotype", add.params = list(),
                        error.plot = "pointrange", legand = NULL, size = NULL, width =
0.7, notch = FALSE, outlier.shape = 20, facet.by = NULL,
                        panel.labs = NULL, short.panel.labs = TRUE,bxp.errorbar
= FALSE, bxp.errorbar.width = 0.4, ggtheme = theme_pubr())+ theme(axis.text.x
   = element_text(angle = 0, hjust = 0.5))+ theme(legend.text =
   element_text(size = 10, colour = "black", face =
"italic"), legend.text.align = 0)+
   theme(axis.text.y = element_text(angle = 0, vjust = 0.5, size = 10))
+
   theme(axis.text = element_text(colour = "black", size = 10))+ theme(axis.line =
   element_line())+
   theme(panel.background = element_rect(fill = "white"),plot.margin =
margin(0.5, 0.5, 0.5, 0.5, "cm"), plot.background = element_rect(colour = NULL,
size = 1))+
   theme(axis.ticks.length.y = unit(.25, "cm"), axis.ticks.length.x = unit(.25, "cm"),
axis.text.x = element_text(margin = margin(t = .3, unit = "cm")))+
   theme(legend.justification = "top")+
   theme(legend.position = "right")+ theme(legend.key =
   element_rect(fill = "white"))+
   theme(legend.title = element_text(face = NULL, size = 10))
+theme(panel.background = element_blank(), axis.text = element_blank())+
   theme(axis.text = element_text(colour = "black", size = 10)+
                theme(axis.line = element_line())+ theme(panel.background =
                element_rect(fill =
"white"),plot.margin = margin(0.5, 0.5, 0.5, 0.5, "cm"), plot.background =
element_rect(colour = "grey"))+
                theme(axis.ticks.length.y = unit(.25, "cm"), axis.ticks.length.x =
unit(.25, "cm"), axis.text.x = element_text(margin = margin(t = .3, unit =
"cm")))+
                theme(axis.title.y = element_text(size = 10, face = "plain", angle =
90))+
                theme(axis.title.x = element_text(size = 10, angle = 0)))
#+stat_compare_means()

Peven_l<-Pe_l + aes(x = fct_inorder(Location)) + theme(legend.position
= "none") + xlab("Location") + ylab("Evenness") Peven_l
```

FARMING TYPE

CHAO1

```
#Chao1 diversity estimates
chao1 <- diversity %>% select(sample_id, chao1)
chao_edited <- merge(chao1, sdata1, by = "sample_id", all = TRUE) #chao_edited
<- chao_edited[c(1:18, 62:86, 19:61),]

#confirming if the chao1 indices are normally distributed
shapiro.test(chao_edited$chao1)

#plotting chao1 distribution boxplot
Pchao_ft <- ggboxplot(chao_edited, "Farming_type","chao1", color =
                       "Farming_type", palette =c("#FBAE17",
"#F52100", "#0071BC"),
                       add = "jitter", linetype = "solid", Family = "Palatino Linotype",
add.params = list(),
                       error.plot = "pointrange", legand = NULL, size = NULL, width =
0.7, notch = FALSE, outlier.shape = 20, facet.by = NULL,
                       panel.labs = NULL, short.panel.labs = TRUE,bxp.errorbar
= FALSE, bxp.errorbar.width = 0.4, ggtheme = theme_pubr())+ theme(axis.text.x
   = element_text(angle = 0, hjust = 0.5)) + theme(legend.text =
   element_text(size = 10, colour = "black", face =
"italic"), legend.text.align = 0)+
   theme(axis.text.y = element_text(angle = 0, vjust = 0.5, size = 10))
+
   theme(axis.text = element_text(colour = "black", size = 10))+ theme(axis.line =
   element_line())+
   theme(panel.background = element_rect(fill = "white"),plot.margin =
margin(0.5, 0.5, 0.5, 0.5, "cm"), plot.background = element_rect(colour = NULL,
size = 1))+
   theme(axis.ticks.length.y = unit(.25, "cm"), axis.ticks.length.x = unit(.25, "cm"),
axis.text.x = element_text(margin = margin(t = .3, unit = "cm")))+
   theme(legend.justification = "top")+
   theme(legend.position = "right")+ theme(legend.key =
   element_rect(fill = "white"))+
   theme(legend.title = element_text(face = NULL, size = 10))
+theme(panel.background = element_blank(), axis.text = element_blank())+
   theme(axis.text = element_text(colour = "black", size = 10)+
               theme(axis.line = element_line())+ theme(panel.background =
               element_rect(fill =
"white"),plot.margin = margin(0.5, 0.5, 0.5, 0.5, "cm"), plot.background =
element_rect(colour = "grey"))+
               theme(axis.ticks.length.y = unit(.25, "cm"), axis.ticks.length.x =
unit(.25, "cm"), axis.text.x = element_text(margin = margin(t = .3, unit =
"cm")))+
```

```
            theme(axis.title.y = element_text(size = 10, face = "plain", angle =
90))+
            theme(axis.title.x = element_text(size = 10, angle =
0)))#+stat_compare_means()
Pchao_ft<-Pchao_ft + aes(x = fct_inorder(Farming_type)) + theme(legend.position
= "none") + xlab("Farming Type") + ylab("Chao1") Pchao_ft
```

FARMING TYPE

OBSERVED

```
#Chao1 diversity estimates
observed <- diversity %>% select(sample_id, observed) observed_edited <-
merge(observed, sdata1, by = "sample_id", all = TRUE)
#chao_edited <- chao_edited[c(1:18, 62:86, 19:61),]

#confirming if the chao1 indices are normally distributed
shapiro.test(observed_edited$observed)

#plotting chao1 distribution boxplot
Pobs_ft <- ggboxplot(observed_edited, "Farming_type","observed", color =
                "Farming_type", palette =c("#FBAE17",
"#F52100", "#0071BC"),
                add = "jitter", linetype = "solid", Family = "Palatino Linotype",
add.params = list(),
                error.plot = "pointrange", legand = NULL, size = NULL, width =
0.7, notch = FALSE, outlier.shape = 20, facet.by = NULL,
                panel.labs = NULL, short.panel.labs = TRUE,bxp.errorbar
= FALSE, bxp.errorbar.width = 0.4, ggtheme = theme_pubr())+ theme(axis.text.x
   = element_text(angle = 0, hjust = 0.5)) + theme(legend.text =
   element_text(size = 10, colour = "black", face =
"italic"), legend.text.align = 0)+
   theme(axis.text.y = element_text(angle = 0, vjust = 0.5, size = 10))
+
   theme(axis.text = element_text(colour = "black", size = 10))+ theme(axis.line =
   element_line())+
   theme(panel.background = element_rect(fill = "white"),plot.margin =
margin(0.5, 0.5, 0.5, 0.5, "cm"), plot.background = element_rect(colour = NULL,
size = 1))+
   theme(axis.ticks.length.y = unit(.25, "cm"), axis.ticks.length.x = unit(.25, "cm"),
axis.text.x = element_text(margin = margin(t = .3, unit = "cm")))+
   theme(legend.justification = "top")+
   theme(legend.position = "right")+ theme(legend.key =
   element_rect(fill = "white"))+
   theme(legend.title = element_text(face = NULL, size = 10))
+theme(panel.background = element_blank(), axis.text = element_blank())+
```

```
   theme(axis.text = element_text(colour = "black", size = 10)+
              theme(axis.line = element_line())+ theme(panel.background =
              element_rect(fill =
"white"),plot.margin = margin(0.5, 0.5, 0.5, 0.5, "cm"), plot.background =
element_rect(colour = "grey"))+
              theme(axis.ticks.length.y = unit(.25, "cm"), axis.ticks.length.x =
unit(.25, "cm"), axis.text.x = element_text(margin = margin(t = .3, unit =
"cm")))+
              theme(axis.title.y = element_text(size = 10, face = "plain", angle =
90))+
              theme(axis.title.x = element_text(size = 10, angle = 0)))#+
stat_compare_means()


Pobs_ft<-Pobs_ft + aes(x = fct_inorder(Farming_type)) + theme(legend.position =
"none") + xlab("Farming Type") + ylab("Observed")

Pobs_ft
```

SHANNON DIVERSITY

```
#Extracting the shannon diversity index
shannon <- diversity %>% select(sample_id, diversity_shannon)

shannon_edited <- merge(shannon, sdata1, by = "sample_id", all = TRUE)
#shannon_edited <- shannon_edited[c(1:18, 62:86, 19:61),]

#confirming if the shannon indices are normally distributed
shapiro.test(shannon_edited$diversity_shannon)

library(ggpubr)


#plotting the boxplots for the shannon index data
P_ft <- ggboxplot(shannon_edited, "Farming_type","diversity_shannon", color =
                      "Farming_type", palette =c("#FBAE17",
"#F52100", "#0071BC"),
                      add = "jitter", linetype = "solid", Family = "Palatino Linotype",
add.params = list(),
                      error.plot = "pointrange", legand = NULL, size = NULL, width =
0.7, notch = FALSE, outlier.shape = 20, facet.by = NULL,
                      panel.labs = NULL, short.panel.labs = TRUE,bxp.errorbar
= FALSE, bxp.errorbar.width = 0.4, ggtheme = theme_pubr())+ theme(axis.text.x
   = element_text(angle = 0, hjust = 0.5))+ theme(legend.text =
   element_text(size = 10, colour = "black", face =
"italic"), legend.text.align = 0)+
   theme(axis.text.y = element_text(angle = 0, vjust = 0.5, size = 10))
+
   theme(axis.text = element_text(colour = "black", size = 10))+
```

```
   theme(axis.line = element_line())+
   theme(panel.background = element_rect(fill = "white"),plot.margin =
margin(0.5, 0.5, 0.5, 0.5, "cm"), plot.background = element_rect(colour = NULL,
size = 1))+
   theme(axis.ticks.length.y = unit(.25, "cm"), axis.ticks.length.x = unit(.25, "cm"),
axis.text.x = element_text(margin = margin(t = .3, unit = "cm")))+
   theme(legend.justification = "top")+
   theme(legend.position = "right")+ theme(legend.key =
   element_rect(fill = "white"))+
   theme(legend.title = element_text(face = NULL, size = 10))
+theme(panel.background = element_blank(), axis.text = element_blank())+
   theme(axis.text = element_text(colour = "black", size = 10)+
               theme(axis.line = element_line())+ theme(panel.background =
               element_rect(fill =
"white"),plot.margin = margin(0.5, 0.5, 0.5, 0.5, "cm"), plot.background =
element_rect(colour = "grey"))+
               theme(axis.ticks.length.y = unit(.25, "cm"), axis.ticks.length.x =
unit(.25, "cm"), axis.text.x = element_text(margin = margin(t = .3, unit =
"cm")))+
               theme(axis.title.y = element_text(size = 10, face = "plain", angle =
90))+
               theme(axis.title.x = element_text(size = 10, angle = 0)))
#+stat_compare_means()

Pshan_ft<-P_ft + aes(x = fct_inorder(Farming_type)) + theme(legend.position =
"none") + xlab("Farming Type") + ylab("Shannon")

Pshan_ft
```

Evenness

```
#Extracting the shannon diversity index
even <- diversity %>% select(sample_id, evenness_pielou)

even_edited <- merge(even, sdata1, by = "sample_id", all = TRUE)
#shannon_edited <- shannon_edited[c(1:18, 62:86, 19:61),]

#confirming if the shannon indices are normally distributed
shapiro.test(even_edited$evenness_pielou)

library(ggpubr)



#plotting the boxplots for the shannon index data
Pe_ft <- ggboxplot(even_edited, "Farming_type","evenness_pielou", color =
                        "Farming_type", palette =c("#FBAE17",
"#F52100", "#0071BC"),
```

```
                        add = "jitter", linetype = "solid", Family = "Palatino Linotype",
add.params = list(),
                        error.plot = "pointrange", legand = NULL, size = NULL, width =
0.7, notch = FALSE, outlier.shape = 20, facet.by = NULL,
                        panel.labs = NULL, short.panel.labs = TRUE,bxp.errorbar
= FALSE, bxp.errorbar.width = 0.4, ggtheme = theme_pubr())+ theme(axis.text.x
   = element_text(angle = 0, hjust = 0.5))+ theme(legend.text =
   element_text(size = 10, colour = "black", face =
"italic"), legend.text.align = 0)+
   theme(axis.text.y = element_text(angle = 0, vjust = 0.5, size = 10))
+
   theme(axis.text = element_text(colour = "black", size = 10))+ theme(axis.line =
   element_line())+
   theme(panel.background = element_rect(fill = "white"),plot.margin =
margin(0.5, 0.5, 0.5, 0.5, "cm"), plot.background = element_rect(colour = NULL,
size = 1))+
   theme(axis.ticks.length.y = unit(.25, "cm"), axis.ticks.length.x = unit(.25, "cm"),
axis.text.x = element_text(margin = margin(t = .3, unit = "cm")))+
   theme(legend.justification = "top")+
   theme(legend.position = "right")+ theme(legend.key =
   element_rect(fill = "white"))+
   theme(legend.title = element_text(face = NULL, size = 10))
+theme(panel.background = element_blank(), axis.text = element_blank())+
   theme(axis.text = element_text(colour = "black", size = 10)+
                theme(axis.line = element_line())+ theme(panel.background =
                element_rect(fill =
"white"),plot.margin = margin(0.5, 0.5, 0.5, 0.5, "cm"), plot.background =
element_rect(colour = "grey"))+
                theme(axis.ticks.length.y = unit(.25, "cm"), axis.ticks.length.x =
unit(.25, "cm"), axis.text.x = element_text(margin = margin(t = .3, unit =
"cm")))+
                theme(axis.title.y = element_text(size = 10, face = "plain", angle =
90))+
                theme(axis.title.x = element_text(size = 10, angle = 0)))
#+stat_compare_means()

Peven_ft<-Pe_ft + aes(x = fct_inorder(Farming_type)) + theme(legend.position =
"none") + xlab("Farming Type") + ylab("Evenness")

Peven_ft

#Label and Merge

require(ggplot2)
require(dplyr)
require(svglite)
```

```
Pchao <-Pchao + labs(tag = "A") Pchao_ft
<- Pchao_ft+ labs(tag = "B") Pchao_l <-
Pchao_l + labs(tag = "C")

Peven<-Peven + labs(tag = "D")
Peven_ft<-Peven_ft + labs(tag = "E")
Peven_l<-Peven_l + labs(tag = "F")

Pshan<-Pshan + labs(tag = "G")
Pshan_ft<-Pshan_ft + labs(tag = "H")
Pshan_l<-Pshan_l + labs(tag = "I")



ggarrange(Pchao,     Pchao_ft,     Pchao_l,
          Peven,    Peven_ft,    Peven_l,
          Pshan, Pshan_ft, Pshan_l,
          ncol = 3, nrow = 3) + geom_point()

ggsave("C:/Users/icipe/Desktop/active_manuscript/soil/dada2_16s/finale
_results/pub/Alpha_Diversity.jpeg",
       width = 20, height = 20, dpi = 600)
ggsave("C:/Users/icipe/Desktop/active_manuscript/soil/dada2_16s/finale
_results/pub/Alpha_Diversity.png",
       width = 20, height = 20, dpi = 600)
ggsave("C:/Users/icipe/Desktop/active_manuscript/soil/dada2_16s/finale
_results/pub/Alpha_Diversity.svg",
       width = 20, height = 20, dpi = 600)
ggsave("C:/Users/icipe/Desktop/active_manuscript/soil/dada2_16s/finale
_results/pub/Alpha_Diversity.tiff",
       width = 20, height = 20, dpi = 600)

#Label and Merge

require(ggplot2)
require(dplyr)
require(svglite)

#Sample Type
#Pchao <-Pchao + labs(tag = "A")
#Peven<-Peven + labs(tag = "B")
#Pshan<-Pshan + labs(tag = "C")

ggarrange(Pchao,
          Peven,
          Pshan,
          ncol = 2, nrow = 2) + geom_point()

ggsave("C:/Users/icipe/Desktop/active_manuscript/soil/dada2_16s/finale
_results/alpha/Sample_Type_Alpha_Diversity.jpeg",
```

```
            width = 12, height = 12, dpi = 600)
ggsave("C:/Users/icipe/Desktop/active_manuscript/soil/dada2_16s/finale
_results/alpha/Sample_Type_Alpha_Diversity.png", width =
            12, height = 12, dpi = 600)
ggsave("C:/Users/icipe/Desktop/active_manuscript/soil/dada2_16s/finale
_results/alpha/Sample_Type_Alpha_Diversity.svg", width =
            12, height = 12, dpi = 600)
ggsave("C:/Users/icipe/Desktop/active_manuscript/soil/dada2_16s/finale
_results/alpha/Sample_Type_Alpha_Diversity.tiff", width =
            12, height = 12, dpi = 600)
#Farming_Type
Pchao_ft <- Pchao_ft+ labs(tag = "A")
Peven_ft<-Peven_ft + labs(tag = "B")
Pshan_ft<-Pshan_ft + labs(tag = "C")

ggarrange(Pchao_ft, Peven_ft, Pshan_ft,
                ncol = 2, nrow = 2) + geom_point()
ggsave("C:/Users/icipe/Desktop/active_manuscript/soil/dada2_16s/finale
_results/alpha/Farming_Type_Alpha_Diversity.jpeg", width =
            12, height = 12, dpi = 600)
ggsave("C:/Users/icipe/Desktop/active_manuscript/soil/dada2_16s/finale
_results/alpha/Farming_Type_Alpha_Diversity.png", width =
            12, height = 12, dpi = 600)
ggsave("C:/Users/icipe/Desktop/active_manuscript/soil/dada2_16s/finale
_results/alpha/Farming_Type_Alpha_Diversity.svg", width =
            12, height = 12, dpi = 600)
ggsave("C:/Users/icipe/Desktop/active_manuscript/soil/dada2_16s/finale
_results/alpha/Farming_Type_Alpha_Diversity.tiff", width =
            12, height = 12, dpi = 600)

#Location
Pchao_l <- Pchao_l + labs(tag = "A")
Peven_l<-Peven_l + labs(tag = "B")
Pshan_l<-Pshan_l + labs(tag = "C")

ggarrange(Pchao_l, Peven_l, Pshan_l,
                ncol = 2, nrow = 2) + geom_point()
ggsave("C:/Users/icipe/Desktop/active_manuscript/soil/dada2_16s/finale
_results/alpha/Location_Alpha_Diversity.jpeg", width =
            12, height = 12, dpi = 600)
ggsave("C:/Users/icipe/Desktop/active_manuscript/soil/dada2_16s/finale
_results/alpha/Location_Alpha_Diversity.png", width
            = 12, height = 12, dpi = 600)
ggsave("C:/Users/icipe/Desktop/active_manuscript/soil/dada2_16s/finale
_results/alpha/Location_Alpha_Diversity.svg", width
            = 12, height = 12, dpi = 600)
ggsave("C:/Users/icipe/Desktop/active_manuscript/soil/dada2_16s/finale
_results/alpha/Location_Alpha_Diversity.tiff", width =
            12, height = 12, dpi = 600)
```

Permutational Multivariate Analysis of Variance

SAMPLE TYPE

Permutational Multivariate Analysis of Variance

```
distme <- get_dist(ps3, distmethod ="bray", method="hellinger") sampleda
<- data.frame(sample_data(ps3), check.names=FALSE) sampleda <-
sampleda[match(colnames(as.matrix(distme)),rownames(sampleda)),,drop=F ALSE]
sampleda$Sample_type <- factor(sampleda$Sample_type) set.seed(1024)
adores <- adonis(distme ~ Sample_type, data=sampleda, permutation=9999)
perm<-data.frame(adores$aov.tab)
write.csv(perm,
"C:/Users/icipe/Desktop/active_manuscript/soil/dada2_16s/finale_result
s/pub/16s_Permutational_Multivariate_Analysis_Sampling_type.csv")
```

FARMING TYPE

Permutational Multivariate Analysis of Variance

```
distme <- get_dist(ps3, distmethod ="bray", method="hellinger") sampleda
<- data.frame(sample_data(ps3), check.names=FALSE) sampleda <-
sampleda[match(colnames(as.matrix(distme)),rownames(sampleda)),,drop=F ALSE]
sampleda$Farming_type <- factor(sampleda$Farming_type) set.seed(1024)
adores <- adonis(distme ~ Farming_type, data=sampleda, permutation=9999)
perm<-data.frame(adores$aov.tab)
write.csv(perm,
"C:/Users/icipe/Desktop/active_manuscript/soil/dada2_16s/finale_result
s/pub/16s_Permutational_Multivariate_Analysis_Farming_type.csv")
```

LOCATION

Permutational Multivariate Analysis of Variance

```
distme <- get_dist(ps3, distmethod ="bray", method="hellinger") sampleda
<- data.frame(sample_data(ps3), check.names=FALSE) sampleda <-
sampleda[match(colnames(as.matrix(distme)),rownames(sampleda)),,drop=F ALSE]
sampleda$Location <- factor(sampleda$Location) set.seed(1024)
adores <- adonis(distme ~ Location, data=sampleda, permutation=9999)
perm<-data.frame(adores$aov.tab)
write.csv(perm,
```

```
"C:/Users/icipe/Desktop/active_manuscript/soil/dada2_16s/finale_result
s/pub/16s_Permutational_Multivariate_Analysis_Location.csv")
```

Biomarker Discovery

Sample Type

```
deres <- diff_analysis(obj = ps3, classgroup = "Sample_type", mlfun = "lda",
                                 filtermod = "pvalue", firstcomfun
                                 = "kruskal_test", firstalpha =
                                 0.05,
                                 strictmod = TRUE, secondcomfun
                                 = "wilcox_test", subclmin = 3,
                                 subclwilc = TRUE,
                                 secondalpha = 0.01,
                                 lda=3)
deres

library(MicrobiotaProcess)
library(ggplot2) library(TDbook)

diffclade_p<-ggdiffclade(obj=deres, #nodedf=dt,
               # factorName="Sample_type",
                layout="radial", skpointsize=0.6,
                cladetext=2,
                linewd=0.2, taxlevel=6,
                # This argument is to remove the branch of unknown
taxonomy.
                reduce=TRUE) +
       scale_fill_manual(values=c("#00AED7", "#009E73"))+ guides(color =
       guide_legend(keywidth = 0.1, keyheight = 0.6,
                                        order = 3,ncol=1)) +
       theme(panel.background=element_rect(fill=NA),
              legend.position="right", plot.margin=margin(0,0,0,0),
              legend.spacing.y=unit(0.02, "cm"),
              legend.title=element_text(size=7.5),
              legend.text=element_text(size=5.5),
              legend.box.spacing=unit(0.02,"cm")
          )

diffclade_p

ggsave("C:/Users/icipe/Desktop/active_manuscript/soil/dada2_16s/
finale_results/biomarker/16s_Biosignature_Sample_Type.jpeg",
```

```
width = 18, height = 18, dpi = 600)

ggsave("C:/Users/icipe/Desktop/active_manuscript/soil/dada2_16s/finale
_results/biomarker/16s_Biosignature_Sample_Type.png", width =
    18, height = 18, dpi = 600)

ggsave("C:/Users/icipe/Desktop/active_manuscript/soil/dada2_16s/finale
_results/biomarker/16s_Biosignature_Sample_Type.svg", width =
    18, height = 18, dpi = 600)

ggsave("C:/Users/icipe/Desktop/active_manuscript/soil/dada2_16s/finale
_results/biomarker/16s_Biosignature_Sample_Type.tiff", width =
    18, height = 18, dpi = 600)
```

SAVE WORKSPACE

```
save.image("C:/Users/icipe/Desktop/active_manuscript/soil/dada2_16s/
finale_results/pub/SOIL_16s.Analysis.Workspace.RData")

library(devtools)
install_github("zdk123/SpiecEasi")
library(SpiecEasi) spiec.out=spiec.easi(ps1,
method="mb",icov.select.params=list(rep.num=20))
```
