# Supplementary material for: Long-term push–pull cropping system shifts soil and maize-root microbiome diversity paving way to resilient farming system
Source: BMC Microbiol. 2024 Mar 18;24:92. doi: 10.1186/s12866-024-03238-z (PMC10946131; doi:10.1186/s12866-024-03238-z)
Supplement: Supplementary file 2 — Supplementary Material 2. [file 12866_2024_3238_MOESM2_ESM.zip › Jalloh A.A et al. Push-pull_ITS_Additional file_2A_stat_analysis-doc.pdf]

R Notebook

```
#Load Packages
suppressPackageStartupMessages({
require(csv)
require(dplyr)
require(tidyr)
library(dada2)
library(Cairo)
library(ggtree)
library(VennDiagram)
library(UpSetR)
library("phyloseq"); packageVersion("phyloseq") # Handling and analysis of high-
throughput microbiome census data. library("vegan");packageVersion("vegan") #
Community Ecology Package. library("ggplot2");packageVersion("ggplot2") #
Create Elegant Data Visualisations Using the Grammar of Graphics.
library("dendextend");packageVersion("dendextend")
library("tidyr");packageVersion("tidyr") library("viridis");packageVersion("viridis")
library("reshape");packageVersion("reshape") #install.packages("remotes")
#remotes::install_github("vmikk/metagMisc")
library(metagMisc) library(janitor)
library(coin) # Conditional Inference Procedures in a Permutation Test Framework.
library(reshape2) # Flexibly Reshape Data: A Reboot of the Reshape Package.
library(ggnewscale) # Multiple Fill and Colour Scales in 'ggplot2'.
library(MicrobiotaProcess) # an R package for analysis, visualization and biomarker
discovery of Microbiome.
library(patchwork)
})

#rm(list=ls())
#loading the ASV taxonomic table
getwd()
tax<-read.table("C:/Users/icipe/Desktop/active_manuscript/soil/dada2_I
TS/ASV_tax_species.tsv", header = T, row.names = 1,sep = "\t") head(tax)
names(tax)
tax<-tax[,-c(9:10)]
#head(tax)
tax<-t(tax)
tax<-as.matrix(tax) #converting tax dataframe to a matrix
#head(tax)
tax<-(t(tax))
```

```
tax<-tax_table(tax) #converting the tax matrix to phyloseq object #class(tax)
#head(tax)

# loading the ASv_counts tables
otu<-read.table("C:/Users/icipe/Desktop/active_manuscript/soil/dada2_I
TS/ASV_table.tsv", header = T, row.names=1,sep="\t")
#otu<-otu[,-c(21)]
head(otu) names(otu)
otu<-otu_table(otu,taxa_are_rows = TRUE) #converting otu matrix to phyloseq
object.
head(otu)

#combine the two physeq object i.e tax,otu physeq
<-phyloseq(otu, tax)
physeq
plot_bar(physeq, fill = "Class")

#importing the metadata
meta<-
read.table("C:/Users/icipe/Desktop/active_manuscript/soil/dada2_ITS/
soil_metadata.tsv",sep = "\t", header = TRUE, row.names = 1) head(meta)
#names(meta) = c("Sample_ID","Animal_Type", "Microbiome.Domain","Location")
names(meta)
head(meta)
meta<-sample_data(meta) #converting the metadata to a phyloseq object

# we now create a third object called random for mergingwith the other three object
library("ape")
random_tree <- rtree(ntaxa(physeq), rooted = TRUE, tip.label =
taxa_names(physeq))
plot(random_tree)

#merging the preceeding 3 objects.
physeq1 <- merge_phyloseq(physeq, meta, random_tree) physeq1
saveRDS(physeq1,
file="C:/Users/icipe/Desktop/active_manuscript/soil/dada2_ITS/soil_fun gi.rds")
#readRDS(file ="/path/to/physeq_rumenbacteria.rds" ) #reading a .rds image

#Read in Data
ps1<-readRDS("C:/Users/icipe/Desktop/active_manuscript/soil/dada2_ITS/
soil_fungi.rds")

#filtering the unwanted sequences
ps2 <- subset_taxa(ps1, (Order!="Chloroplast") | is.na(Order))
```

```
ntaxa(ps2)
ps2 <- subset_taxa(ps2, (Phylum!="Chloroflexi") | is.na(Phylum)) ntaxa(ps2)
ps2<- subset_taxa(ps2, (Family!="Mitochondria") | is.na(Family)) ntaxa(ps2)
ps2<- subset_taxa(ps2, (Kingdom!="Archaea") | is.na(Kingdom)) ntaxa(ps2)
ps2<- subset_taxa(ps2, (Kingdom!="Eukaryota") | is.na(Kingdom)) ntaxa(ps2)

ps2<-subset_taxa(ps2, !is.na(Phylum) & !Phylum %in% c("", "uncharacterized"))
ntaxa(ps2)

ps3 <- prune_taxa(taxa_sums(ps2) > 20, ps2) ps3

#Extracting the filtered taxonomy and feature tables for barplot plotting
tax_table <- phyloseq_to_df(ps3, addtax = T, addtot = F, addmaxrank = F)
cumulation <- tax_table %>% adorn_totals(c("col"))
cumulation <- cumulation[order(cumulation$Total, decreasing = TRUE),]

#merging the blast taxonomic classification to blast abundance table merged_data
<- tax_table
write.csv(merged_data,
file="C:/Users/icipe/Desktop/active_manuscript/soil/dada2_ITS/Supp_Tax
onomic_Classification.csv")

#grouping the data (entire dataset): Genus, Species and sample names
Featured_table <- merged_data[,c(4,10:42)]
group <- Featured_table %>%
   group_by(Phylum)%>%
   summarise_if(is.numeric, sum)
#Groups the data in the defined order which eases downstream analysis group <-
Featured_table %>% group_by(Phylum)%>%
   summarise_each(funs(sum),
"R1","R10","R12","R13","R14","R15","R16","R17","R18","R19","R2","R20",
"R3","R4","R5","R6","R7","R8","R9","S1","S10","S13","S14","S15","S16",
"S2","S3","S4","S5","S6","S7","S8","S9")
#group<-group[-c(1),]
head(group)
```

TREATMENTS

```
#creating multiple dataframes for the different treatments

ps<-group[,c(1,20,23,25,28,30,32,34)] # Push pull soil
ms<-group[,c(1,21,22,24,26,27,29,31,33)] # Monoculture soil pr<-
group[,c(1,2,5,7,9,11,13,15,17,19)] # Pushpull root
mr<-group[,c(1,3,4,6,8,10,12,14,16,18)] # Monoculture root
```

```
soil<-group[,c(1,20:34)]
root<-group[,c(1:19)]
all<-group

dim(ps)
ps_total <- ps %>% adorn_totals(c("col"))
ps_total <- mutate(ps_total, ps=rowSums(ps_total[9])/8) ps_total
<- ps_total[,c(1,10)]

dim(ms)
ms_total <- ms %>% adorn_totals(c("col"))
ms_total <- mutate(ms_total, ms=rowSums(ms_total[10])/9)
ms_total <- ms_total[,c(1,11)]

dim(pr)
pr_total <- pr %>% adorn_totals(c("col"))
pr_total <- mutate(pr_total, pr=rowSums(pr_total[11])/10)
pr_total <- pr_total[,c(1,12)]

dim(mr)
mr_total <- mr %>% adorn_totals(c("col"))
mr_total <- mutate(mr_total, mr=rowSums(mr_total[11])/10)
mr_total <- mr_total[,c(1,12)]

dim(soil)
soil_total <- soil%>% adorn_totals(c("col"))
soil_total <- mutate(soil_total, soil=rowSums(soil_total[17])/16) soil_total <-
soil_total[,c(1,18)]

dim(root)
root_total <- root %>% adorn_totals(c("col"))
root_total <- mutate(root_total, root=rowSums(root_total[20])/19) root_total <-
root_total[,c(1,21)]

ps_total ms_total pr_total mr_total

# Merging
##merged <- Reduce(function(x,y) merge(x,y,by="Phylum",all=TRUE), ##
                              list(ps_total,ms_total,pr_total, mr_total ))

##names(merged)<-c('Phylum','Push Soil','Mono Soil','Push Root', 'Mono Root')

merged <- Reduce(function(x,y) merge(x,y,by="Phylum",all=TRUE),
                           list(root_total,soil_total))

names(merged)<-c('Phylum','Root','Soil')

#calculating the total abundance per genus and ordering from the most
```

```
abundant to the lowest
cumulation <- merged %>% adorn_totals(c("col"))
cumulation <- cumulation[order(cumulation$Total, decreasing = TRUE),]
cumulation$perc = cumulation$Total / sum(cumulation$Total) * 100

tired<-head(cumulation$Phylum, n=10) #
Using R base append()
#install.packages('rlist') library('rlist')
li2 <- append(tired,"Others") print(li2)
genus_Rep <- li2

group <- aggregate(merged[-1], list(Phylum = replace(merged$Phylum,!
(merged$Phylum %in% genus_Rep), "Others")), sum)
#View(group)


#PS<-group[,c(1:2)]
#MS<- group[,c(1:3)]
#PR<- group[,c(1:4)]
#MR<- group[,c(1:5)]
All<- group[,c(1:3)]
```

Viewing Sample Diversity

```
#install.packages("janitor")
library(janitor)
#converting the abudances into percentage
bar_all <- adorn_percentages(All, denominator = "col", na.rm = T) bar_all %>%
   adorn_totals("row") %>%
   adorn_pct_formatting()
dist_all<-bar_all %>%
   adorn_totals("row") %>%
   adorn_pct_formatting()
dist_all write.csv(dist_all,
"C:/Users/icipe/Desktop/active_manuscript/soil/dada2_ITS/Fungi_10_Phyl
um_Abundance_Sample_Type.csv")


#gathering the data
bar_all <- bar_all %>%
   gather(value = "abundance", key = "Sample_type", -Phylum) bar_all <-
as.data.frame(gsub("\\(", " (", as.matrix(bar_all)))

# coerce the dataframe columns into respective data type bar_all$Phylum <-
as.factor(bar_all$Phylum)
```

```
bar_all$Farming_type <- as.character(bar_all$Sample_type) bar_all$abundance
<- as.numeric(bar_all$abundance)

#ordering the data for plotting
bar_all$Phylum <- reorder(bar_all$Phylum, bar_all$abundance) bar_all$Phylum
<- factor(bar_all$Phylum, levels=rev(levels(bar_all$Phylum)))
bar_all$Phylum <- factor(bar_all$Phylum,
                         levels=genus_Rep)

# Defining the color pallete
myPalette <- c("#1B9E77", "#D95F02", "#7570B3", "#E7298A", "#99000D",
"#E6AB02", "#A6761D", "#666666","#FDCDAC", "#1F78B4", "#B2DF8A",
"#33A02C", "#CBD5E8", "#E31A1C", "#FDBF6F",
"#FF7F00","#4A1486","#C0C0C0","#B3E2CD","#FFFF33",  "#5172b2","#F4CAE4",
"#E6F5C9", "#FCFBFD","#139BF1","#09FF00","#065535", "#1D91C0",
"#C0FFEE","#B35806","#0C2C84","#D0ED0E","#092617","#499976","#4D5D53",
"#E48400","#6082B6","#316689","#CEFB02","#738678","#645452","#EEA47FFF
", "#00539CFF", "#FC766AFF", "#42EADDFF", "#00A4CCFF", "#69B3BB",
"#B589D6","#D1DFB7","#97BC62FF","#D198C5FF","#000000", "#CBCE91FF",
"#616247FF", "#D64161FF","#435E55FF", "#DD4132FF","#CE4A7EFF",
"#BD7F37FF","#FFA351FF","#185E57", "#FCE3E3", "#EF6C6C", "#EF6C9A",
"#93103E", "#F7E3FC", "#D56CEF", "#BD19E6", "#8D6CEF", "#DBD1FA",
"#BFC8F8", "#1531BC", "#9FA5F3", "#95C7F3", "#6CEFC8", "#6CEF84",
"#91E619", "#B7CEEC", "#9AFEFF", "#57FEFF", "#78C7C7", "#46C7C7",
"#00A36C", "#728C00", "#4E9268", "#6CC417", "#64E986", "#F5E216",
"#FFCE44", "#8B8000", "#660000", "#610541", "#E56E94", "#F660AB",
"#E3319D", "#FF77FF", "#C45AEC", "#6960EC", "#736AFF", "#F9B7FF",
"#FCDFFF", "#D291BC", "#614051", "#FEA3AA", "#7D0541")

length(myPalette)

# Definig the names in italics
guide_italics <- guides(fill = guide_legend(label.theme = element_text(size = 15,
face = "italic", colour = "Black", angle = 0)))
```

Plotting barplot

```
library(Cairo)
library(forcats)

#plotting the barplot
p_all <- ggplot(bar_all,aes(x = fct_inorder(Sample_type), y = abundance),
labs(fill= Phylum), group=row.names(bar_all))+ xlab("Sample Type")+
ylab("abundance") + geom_col(aes(fill = Phylum),position =
position_stack(reverse = FALSE))+
  theme(axis.text.x = element_text(angle = 72,size = 15, hjust = 1,
```

```
face = "italic", family = "Arial"))+ scale_fill_manual(values =
     myPalette)+
    #guides(fill = guide_legend(reverse = FALSE))+ guide_italics+
   theme(legend.text = element_text(size = 8, colour = "black", face = "italic",
family = "Arial"), legend.text.align = 0)+
   theme(axis.text.y = element_text(angle = 0, vjust = 0.5, size = 8, family =
"Arial"))+
   theme(axis.text = element_text(colour = "black", size = 8, family = "Arial"))+
   theme(axis.line = element_line())+
   theme(panel.background = element_rect(fill = "white"),plot.margin =
margin(0.1, 0.1, 0.1, 0.1, "cm"), plot.background = element_rect(colour = NULL,
size = 1))+
   theme(axis.ticks.length.y = unit(.15, "cm"), axis.ticks.length.x = unit(.25,
"cm"), axis.text.x = element_text(margin = margin(t = .3, unit = "cm")))+
   theme(legend.position = "right", legend.justification = "top", legend.direction =
"vertical", legend.text = element_text(size = 10))+
   theme(legend.key = element_rect(fill = "white"))+ theme(legend.title =
   element_text(face = NULL, size = 8, family =
"Arial"))+
   theme(panel.background = element_blank(), axis.text = element_blank())+
   theme(axis.text = element_text(colour = "black", size = 8, family = "Arial"))

#install.packages('extrafont')
library(extrafont) #font_import
#BiocManager::install("ggpubr")

#plot4_60<-p_all + scale_y_continuous(labels =
scales::percent_format(accuracy = 1)) + xlab("Site")
plot4_10<-p_all
plot4_10

ggsave("C:/Users/icipe/Desktop/active_manuscript/soil/dada2_ITS/
Fungi_10_Phylum_Abundance_Sample_Type.jpeg",
          width = 12, height = 12, dpi = 600)

ggsave("C:/Users/icipe/Desktop/active_manuscript/soil/dada2_ITS/Fungi_
10_Phylum_Abundance_Sample_Type.png",
          width = 12, height = 12, dpi = 600)

ggsave("C:/Users/icipe/Desktop/active_manuscript/soil/dada2_ITS/Fungi_
10_Phylum_Abundance_Sample_Type.svg",
          width = 12, height = 12, dpi = 600)

ggsave("C:/Users/icipe/Desktop/active_manuscript/soil/dada2_ITS/Fungi_
```

```
10_Phylum_Abundance_Sample_Type.tiff", width
          = 12, height = 12, dpi = 600)
```

ALPHA DIVERSITY

```
ps3 set.seed(1024)
rareres <- get_rarecurve(obj=ps3, chunks=400)

p_rare <- ggrarecurve(obj=rareres,
                              indexNames=c("Observe","Chao1","ACE","Shannon"),
                              ) +
              theme(legend.spacing.y=unit(0.01,"cm"),
                       legend.text=element_text(size=4))

prare1 <- ggrarecurve(obj=rareres, factorNames="Sample_type",
                              indexNames=c("Observe","Chao1","ACE","Shannon")
                              ) +
              scale_fill_manual(values=c("#89C5DA", "#DA5724", "#74D944",
"#CE50CA"))+
              scale_color_manual(values=c("#89C5DA", "#DA5724", "#74D944",
"#CE50CA"))+
              theme_bw()+ theme(axis.text=element_text(size=8),
panel.grid=element_blank(),
                       strip.background = element_rect(colour=NA,fill="grey"),
                       strip.text.x = element_text(face="bold"))

prare2 <- ggrarecurve(obj=rareres,
                              factorNames="Sample_type",
                              shadow=FALSE,
                              indexNames=c("Observe", "Chao1",
"ACE","Shannon")
                              ) +
              scale_color_manual(values=c("#89C5DA", "#DA5724", "#74D944",
"#CE50CA"))+
              theme_bw()+ theme(axis.text=element_text(size=8),
panel.grid=element_blank(),
                       strip.background = element_rect(colour=NA,fill="grey"),
                       strip.text.x = element_text(face="bold")) p_rare
/ prare1 / prare2

alphaobj <- get_alphaindex(ps3)
head(as.data.frame(alphaobj))

p_alpha_ty <- ggbox(alphaobj, geom="violin", factorNames="Sample_type") +
```

```
                scale_fill_manual(values=c("#89C5DA", "#DA5724", "#74D944",
"#CE50CA"))+
                theme(strip.background = element_rect(colour=NA, fill="grey"))
p_alpha_ty

ggsave("C:/Users/icipe/Desktop/active_manuscript/soil/dada2_ITS/
Alpha_Diversity_Sample_Type.jpeg",
          width = 18, height = 12, dpi = 600)

ggsave("C:/Users/icipe/Desktop/active_manuscript/soil/dada2_ITS/Alpha_
Diversity_Sample_Type.png",
          width = 18, height = 12, dpi = 600)

ggsave("C:/Users/icipe/Desktop/active_manuscript/soil/dada2_ITS/Alpha_
Diversity_Sample_Type.svg",
          width = 18, height = 12, dpi = 600)

ggsave("C:/Users/icipe/Desktop/active_manuscript/soil/dada2_ITS/Alpha_
Diversity_Sample_Type.tiff",
          width = 18, height = 12, dpi = 600)

alphaobj <- get_alphaindex(ps3)
head(as.data.frame(alphaobj))

p_alpha_lc <- ggbox(alphaobj, geom="violin", factorNames="Location") +
                scale_fill_manual(values=c("#89C5DA", "#DA5724", "#74D944",
"#CE50CA"))+
                theme(strip.background = element_rect(colour=NA, fill="grey"))
p_alpha_lc

alphaobj <- get_alphaindex(ps3)
head(as.data.frame(alphaobj))

p_alpha_ft <- ggbox(alphaobj, geom="violin", factorNames="Farming_type") +
                scale_fill_manual(values=c("#89C5DA", "#DA5724", "#74D944",
"#CE50CA"))+
                theme(strip.background = element_rect(colour=NA, fill="grey"))
p_alpha_ft


ggarrange(p_alpha_ty, p_alpha_lc, p_alpha_ft, ncol = 1,
              nrow = 3) + geom_point()


ggsave("C:/Users/icipe/Desktop/active_manuscript/soil/dada2_ITS/Combin
ed_Alpha_Diversity_Sample_Type.jpeg",
```

```
          width = 20, height = 20, dpi = 600)

ggsave("C:/Users/icipe/Desktop/active_manuscript/soil/dada2_ITS/Combin
ed_Alpha_Diversity_Sample_Type.png",
          width = 20, height = 10, dpi = 600)

ggsave("C:/Users/icipe/Desktop/active_manuscript/soil/dada2_ITS/Combin
ed_Alpha_Diversity_Sample_Type.svg",
          width = 20, height = 20, dpi = 600)

ggsave("C:/Users/icipe/Desktop/active_manuscript/soil/dada2_ITS/Combin
ed_Alpha_Diversity_Sample_Type.tiff",
          width = 20, height = 20, dpi = 600)
```

Venn or Upset plot

```
vennlist <- get_vennlist(obj=ps3, factorNames="Sample_type") upsetda <-
get_upset(obj=ps3, factorNames="Sample_type")

vennp <- venn.diagram(vennlist,
                              height=5,
                              width=5,
                              filename=NULL,
                              fill=c("#89C5DA", "#DA5724"), cat.col=c("#89C5DA",
                             "#DA5724"),
                              alpha = 0.85, fontfamily
                              = "serif", fontface =
                              "bold", cex = 1.2,
                              cat.cex = 1.3, cat.default.pos =
                              "outer", cat.dist=0.1,
                              margin = 0.1,
                              lwd = 3,
                              lty ='dotted', imagetype =
                              "svg")
grid::grid.draw(vennp)

upset(upsetda, sets=unique(as.vector(sample_data(ps3)$Sampling_type)),

        sets.bar.color = "#56B4E9",
        order.by = "freq",
        empty.intersections = "on")
```

Beta Analysis

PCA analysis

```
# If the input was normalized, the method parameter should be setted NULL.
pcares <- get_pca(obj=ps3, method="hellinger") #
Visulizing the result
```

```
pcaplot1 <- ggordpoint(obj=pcares, biplot=TRUE,
                              factorNames=c("Sample_type"), ellipse=TRUE) +
                scale_color_manual(values=c("#89C5DA", "#DA5724",
"#74D944", "#CE50CA")) +
                scale_fill_manual(values=c("#89C5DA", "#DA5724", "#74D944",
"#CE50CA"))
pcaplot1
# pc = c(1, 3) to show the first and third principal components. pcaplot2 <-
ggordpoint(obj=pcares, pc=c(1, 3), biplot=TRUE,
                              factorNames=c("Sample_type"), ellipse=TRUE) +
                scale_color_manual(values=c("#89C5DA", "#DA5724",
"#74D944", "#CE50CA")) +
                scale_fill_manual(values=c("#89C5DA", "#DA5724", "#74D944",
"#CE50CA"))

pcaplot2
#pcaplot1 | pcaplot2



ggarrange(pcaplot1, pcaplot2,
            ncol = 1, nrow = 2) + geom_point()


ggsave("C:/Users/icipe/Desktop/active_manuscript/soil/dada2_ITS/Combin
ed_Beta_Diversity_PCA.jpeg",
        width = 18, height = 18, dpi = 600)

ggsave("C:/Users/icipe/Desktop/active_manuscript/soil/dada2_ITS/Combin
ed_Beta_Diversity_PCA.png",
        width = 18, height = 18, dpi = 600)

ggsave("C:/Users/icipe/Desktop/active_manuscript/soil/dada2_ITS/Combin
ed_Beta_Diversity_PCA.svg",
        width = 18, height = 18, dpi = 600)

ggsave("C:/Users/icipe/Desktop/active_manuscript/soil/dada2_ITS/Combin
ed_Beta_Diversity_PCA.tiff",
        width = 18, height = 18, dpi = 600)
```

PCoA analysis

```
# distmethod
# "unifrac",  "wunifrac", "manhattan", "euclidean", "canberra", "bray",
"kulczynski" ...(vegdist, dist)
pcoares <- get_pcoa(obj=ps3, distmethod="bray", method="hellinger") #
Visualizing the result
pcoaplot1 <- ggordpoint(obj=pcoares, biplot=TRUE,
speciesannot=FALSE,showsample=TRUE,
                              factorNames=c("Sample_type","Location"),
```

```
ellipse=TRUE) +
                scale_color_manual(values=c("#89C5DA", "#DA5724", "#74D944",
"#CE50CA")) +
                scale_fill_manual(values=c("#89C5DA", "#DA5724", "#74D944",
"#CE50CA"))
pcoaplot1
# first and third principal co-ordinates
pcoaplot2 <- ggordpoint(obj=pcoares, pc=c(1, 3), biplot=TRUE,
speciesannot=TRUE,
                                factorNames=c("Sample_type"), ellipse=TRUE) +
                 scale_color_manual(values=c("#89C5DA", "#DA5724",
"#74D944", "#CE50CA")) +
                 scale_fill_manual(values=c("#89C5DA", "#DA5724", "#74D944",
"#CE50CA"))


#pcoaplot1 | pcoaplot2

ggarrange(pcoaplot1, pcoaplot2,
             ncol = 1, nrow = 2) + geom_point()



ggsave("C:/Users/icipe/Desktop/active_manuscript/soil/dada2_ITS/Combin
ed_Beta_Diversity_PCoA.jpeg",
          width = 18, height = 18, dpi = 600)

ggsave("C:/Users/icipe/Desktop/active_manuscript/soil/dada2_ITS/Combin
ed_Beta_Diversity_PCoA.png",
          width = 18, height = 18, dpi = 600)

ggsave("C:/Users/icipe/Desktop/active_manuscript/soil/dada2_ITS/Combin
ed_Beta_Diversity_PCoA.svg",
          width = 18, height = 18, dpi = 600)

ggsave("C:/Users/icipe/Desktop/active_manuscript/soil/dada2_ITS/Combin
ed_Beta_Diversity_PCoA.tiff",
          width = 18, height = 18, dpi = 600)
```

Permutational Multivariate Analysis of Variance

```
distme <- get_dist(ps3, distmethod ="bray", method="hellinger") sampleda
<- data.frame(sample_data(ps3), check.names=FALSE) sampleda <-
sampleda[match(colnames(as.matrix(distme)),rownames(sampleda)),,drop=F
ALSE]
sampleda$Sample_type <- factor(sampleda$Sample_type)
set.seed(1024)
adores <- adonis(distme ~ Sample_type, data=sampleda, permutation=9999)
perm<-data.frame(adores$aov.tab)
```

```
write.csv(perm,
"C:/Users/icipe/Desktop/active_manuscript/soil/dada2_ITS/ITS_Permutati
onal_Multivariate_Analysis.csv")
```

hierarchical cluster analysis of samples

```
hcsample <- get_clust(obj=ps3, distmethod="bray",
                      method="hellinger", hclustmethod="average")
# rectangular layout
cplot1 <- ggclust(obj=hcsample,
                  layout = "rectangular",
                  pointsize=1, fontsize=0,
                  factorNames=c("Sample_type")
          ) +
          scale_color_manual(values=c("#89C5DA", "#DA5724",
"#74D944", "#CE50CA")) +
          theme_tree2(legend.position="right", plot.title =
          element_text(face="bold",
lineheight=25,hjust=0.5)) #
circular layout
cplot2 <- ggclust(obj=hcsample,
                  layout = "circular",
                  pointsize=1, fontsize=2,
                  factorNames=c("Sample_type")
      ) +
      scale_color_manual(values=c("#89C5DA", "#DA5724", "#74D944",
"#CE50CA")) +
      theme(legend.position="right")
```

biomarker discovery

```
deres <- diff_analysis(obj = ps3, classgroup = "Sample_type", mlfun = "lda",
                       filtermod = "pvalue", firstcomfun
                       = "kruskal_test", firstalpha =
                       0.05,
                       strictmod = TRUE, secondcomfun
                       = "wilcox_test", subclmin = 3,
                       subclwilc = TRUE,
                       secondalpha = 0.01,
                       lda=3)
deres

diffclade_p <- ggdiffclade(
                  obj=deres,
                  alpha=0.3,
                  linewd=0.15,
                  skpointsize=0.6,
                  layout="radial",
```

```
                              taxlevel=3,
                              removeUnkown=TRUE,
                              reduce=TRUE # This argument is to remove the branch of
unknown taxonomy.
                        ) +
                        scale_fill_manual(
                              values=c("#89C5DA", "#DA5724", "#74D944",
"#CE50CA")
                        ) +
                        guides(color = guide_legend(
                                          keywidth = 0.1,
                                          keyheight = 0.6,
                                          order = 3, ncol=1)
                        ) +
                        theme(
                              panel.background=element_rect(fill=NA),
                              legend.position="right", plot.margin=margin(0,0,0,0),
                              legend.spacing.y=unit(0.02, "cm"),
                              legend.title=element_text(size=7),
                              legend.text=element_text(size=6),
                              legend.box.spacing=unit(0.02,"cm")
                        )
diffclade_p


ggsave("C:/Users/icipe/Desktop/active_manuscript/soil/dada2_ITS/ITS_Bi
osignature.jpeg",
           width = 18, height = 18, dpi = 600)

ggsave("C:/Users/icipe/Desktop/active_manuscript/soil/dada2_ITS/ITS_Bi
osignature.png",
           width = 18, height = 18, dpi = 600)

ggsave("C:/Users/icipe/Desktop/active_manuscript/soil/dada2_ITS/ITS_Bi
osignature.svg",
           width = 18, height = 18, dpi = 600)

ggsave("C:/Users/icipe/Desktop/active_manuscript/soil/dada2_ITS/ITS_Bi
osignature.tiff",
           width = 18, height = 18, dpi = 600)

diffbox <- ggdiffbox(obj=deres, box_notch=FALSE, colorlist=c("#89C5DA",
                     "#DA5724", "#74D944", "#CE50CA"),
l_xlabtext="relative abundance")
diffbox

es_p <- ggeffectsize(obj=deres,
                              lineheight=0.1,
```

```
                              linewidth=0.3) +
           scale_color_manual(values=c("#89C5DA", "#DA5724", "#74D944", 
"#CE50CA"))

es_p

save.image("C:/Users/icipe/Desktop/active_manuscript/soil/dada2_ITS/
SOIL_ITS.Analysis.RData")
```
